# Efficient synthesis of fluorinated triphenylenes with enhanced arene–perfluoroarene interactions in columnar mesophases

**DOI:** 10.3762/bjoc.20.270

**Published:** 2024-12-16

**Authors:** Yang Chen, Jiao He, Hang Lin, Hai-Feng Wang, Ping Hu, Bi-Qin Wang, Ke-Qing Zhao, Bertrand Donnio

**Affiliations:** 1 College of Chemistry and Materials Science, Sichuan Normal University, Chengdu 610066, China; 2 Institut de Physique et Chimie des Matériaux de Strasbourg (IPCMS), CNRS-Université de Strasbourg (UMR 7504), F-67034 Strasbourg, France

**Keywords:** arene–perfluoroarene interaction, decafluorobiphenyl, fluorinated triphenylene, fluoroarene nucleophilic substitution, organolithium

## Abstract

The high potential of non-covalent arene–fluoroarene intermolecular interactions in the design of liquid crystals lies in their ability to strongly promote self-assembly, improve the order and stability of the supramolecular mesophases, and enable tuneability of the optical and electronic properties, which can potentially be exploited for advanced applications in display technologies, photonic devices, sensors, and organic electronics. We recently successfully reported the straightforward synthesis of several mesogens containing four lateral aliphatic chains and derived from the classical triphenylene core self-assembling in columnar mesophases based on this paradigm. These mesogenic compounds were simply obtained in good yields by the nucleophilic substitution (S_N_FAr) of various types of commercially available fluoroarenes with the electrophilic organolithium derivatives 2,2'-dilithio-4,4',5,5'-tetraalkoxy-1,1'-biphenyl (2Li-**BP***n*). In a continuation of this study, aiming at testing the limits of the reaction and providing a large diversity of structures, a structurally related series of compounds is reported here, namely 1,2,4-trifluoro-6,7,10,11-tetraalkoxy-3-(perfluorophenyl)triphenylenes (**F***n*). They were obtained by reacting the above mentioned 2,2’-dilithiobiphenyl derivatives with decafluorobiphenyl, C_6_F_5_–C_6_F_5_. These compounds differ from the previously reported series, 1,2,4-trifluoro-6,7,10,11-tetraalkoxy-3-aryltriphenylenes (**PH***n*), solely by the substitution of the terminal phenyl ring with a pentafluorophenyl ring. Thus, as expected, they display a Col_hex_ mesophase over large temperature ranges, with only small differences in the mesophase stability and transition temperatures. Furthermore, the presence of the terminal fluorophenyl group enables a subsequent second annulation, yielding a new series of extended polyaromatic mesomorphic compounds, i.e., 1,1',3,3',4,4'-hexafluoro-6,6',7,7',10,10',11,11'-octaalkoxy-2,2'-bitriphenylene (**G***nm*) which were found to display a Col_rec_ mesophase. The specific nucleophilic substitution patterns of the **F***n* derivatives and the antiparallel stacking mode into columnar structures stabilized by arene–perfluoroarene intermolecular interactions were confirmed by the single-crystal structure of the alkoxy-free side chain analog, i.e., 1,2,4-trifluoro-3-(perfluorophenyl)triphenylene (**F**). UV–vis absorption and fluorescence emission spectroscopies reveal green photoluminescence with fluorescence quantum yields of up to 33% for the **F***n* derivatives. The *J*-aggregation for the inner fluorine-substituted dimers **G***nm* is energetically and stereoelectronically more favorable and **G**66 exhibits thin-film fluorescence with a large red-shift of the emission peak.

## Introduction

Non-covalent arene–fluoroarene intermolecular interactions [1–2] are drawing increasing attention due to their critical role in the engineering of functional and complex supramolecular assemblies [3–11], ranging from rigid crystalline architectures [3–8] to soft liquid crystalline materials [9–11]. Their unique properties originate from the high electronegativity of the fluorine atoms, inserted in the aromatic rings, which considerably modifies the dipole moment of the corresponding fluorinated aromatic rings with respect to their hydrogenated homologs, thus influencing their behavior, binding affinities, and optoelectronic properties. These interactions already represent an effective tool for the design of liquid crystalline materials [3–8]. Rod-like liquid crystalline molecules with fluorine-substituted arenes are ubiquitous in the displays industry [12]. They are also gaining importance in the design of π-conjugated polycyclic aromatic discotic liquid crystals (*F*-DLCs) [13–17] of interest for organic electronics and optical advanced materials, as they tend to promote more efficient molecular stacking into columns than their purely hydrogenated counterparts [18–19], thereby improving one-dimensional charge transport properties [20–22] in combination with tunable absorption and emission of visible light. Polar nematic phase [23] and chiral columnar phase materials [24] based on polar fluorobenzene rings have also recently emerged as interesting new classes of fluorous materials, revealing their enormous potential in the high-tech fields.

Although, F-DLCs seem to show unique and advantageous physical properties, their numbers and structural variation types are very limited due partially to several synthetic challenges [25–34]. Their syntheses usually are based on the direct transformation of commercial perfluoroarene chemical blocks and reagents, involving catalyzed C–F-bond activation and cross-coupling reactions, usually requiring precious transition-metal catalysts and tedious synthetic routes [28–34]. Therefore, low-cost and facile synthetic strategies are desired to increase their structural and functional diversity. In the modern organic synthetic tool-box, the fluoroarene nucleophilc substitution (S_N_FAr) reaction possesses many outstanding advantages in the synthesis of π-conjugated functional molecules: the electrophiles are plentiful and include cheaply available perfluorobenzene, perfluoropyridine, perfluoronaphthalene, decafluorobiphenyl, and many other synthesized perfluoroarenes, and the nucleophiles are also abundant and contain aryllithium, conjugated organic dilithium reagents, phenols and benzenethiols, etc. [35–43].

**Figure 1:**
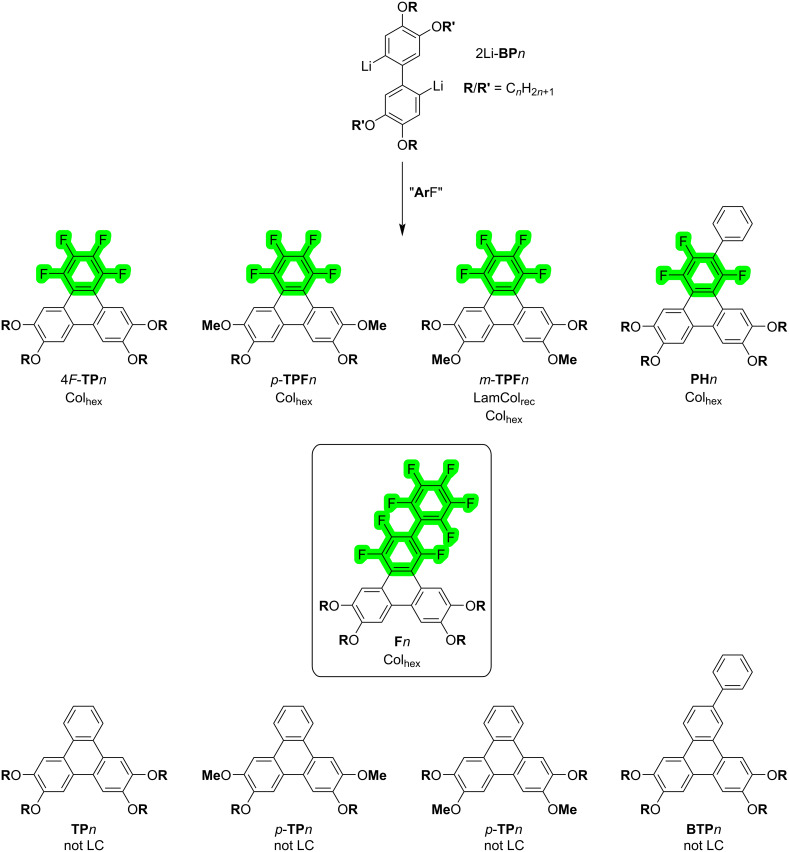
Fluorotriphenylene derivatives and their nonfluorinated homologs obtained by S_N_FAr from 2,2'-dilithio-4,4',5,5'-tetraalkoxy-1,1'-biphenyl (2Br-**BP***n* → 2Li-**BP***n*) e.g., 4*F*-**TP***n* [44], *p*-**TPF***n* [46], *m*-**TPF***n* [46], **PH***n* [45], and **F***n* (this work); **BTP***n* was synthesized by a Suzuki–Scholl reaction sequence (Scheme S3, Supporting Information File 1).

We recently reported the high versatility of these intermolecular interactions in the design of several Janus-like discotic mesogens (Figure 1) [44–47]. A first study dealt with the synthesis of two sets of compounds, namely 1,2,3,4-tetrafluoro-6,7,10,11-tetraalkxoytriphenylenes (4*F*-**TP***n*) and 9,10,11,12,13,14-hexafluoro-2,3,6,7-tetraalkoxybenzo[*f*]tetraphenes (6*F*-**BTP***n*) [44], obtained by the straightforward nucleophilic substitution of fluoroarenes (S_N_FAr) between 2,2'-dilithio-4,4',5,5'-tetraalkoxy-1,1'-biphenyl (2Li-**BP***n*) derivatives and hexafluorobenzene, C_6_F_6_ (4*F*-**TP***n*), on the one hand, and octafluoronaphthalene, C_10_F_8_ (6*F*-**BTP***n*), on the other. With only four alkoxy chains, these polar “Janus” mesogens [33,44] display a columnar hexagonal mesophase over broader temperature ranges and higher mesophase stability than the archetypical 2,3,6,7,10,11-hexa(alkoxy)triphenylene counterparts [48], whereas the corresponding hydrogenated 2,3,6,7-tetraalkoxytriphenylene counterparts (**TP***n*) were not mesomorphic. Testing further this approach to evaluate the persistence of mesomorphism in this family of compounds, another set of related compounds but with inhomogeneous chain substitution patterns, namely 7,10-dialkoxy-1,2,3,4-tetrafluoro-6,11-dimethoxytriphenylene (*p*-**TPF***n*) and 6,11-dialkoxy-1,2,3,4-tetrafluoro-7,10-dimethoxytriphenylene (*m*-**TPF***n*), were synthesized by this method [46]. Both isomers also displayed liquid crystalline properties, despite an even larger deficit of alkyl chains, although the inhomogeneous chain distribution had a net impact on both stability and nature of the mesophases. The versatility of this synthetic approach allows us to synthesize another set of mesomorphic compounds, based on a triphenylene core, 1,2,4-trifluoro-6,7,10,11-tetra(alkyloxy)-3-phenyltriphenylenes (**PH***n*, and extended to other aryl derivatives) by reacting lipophilic 2,2’-dilithiobiphenyl derivatives with the bulkier pentafluorobiphenyl, C_6_F_5_–C_6_H_5_. All these compounds display large mesomorphic ranges again, with the final phenyl ring being immersed with both the aliphatic continuum and the columns of stacked aromatic cores [45]. All these structural investigations revealed the great resilience of such a molecular system to important structural changes, and the essential role of the fluorinated phenyl moieties in the induction and stability of liquid crystalline mesophases.

**Scheme 1:**
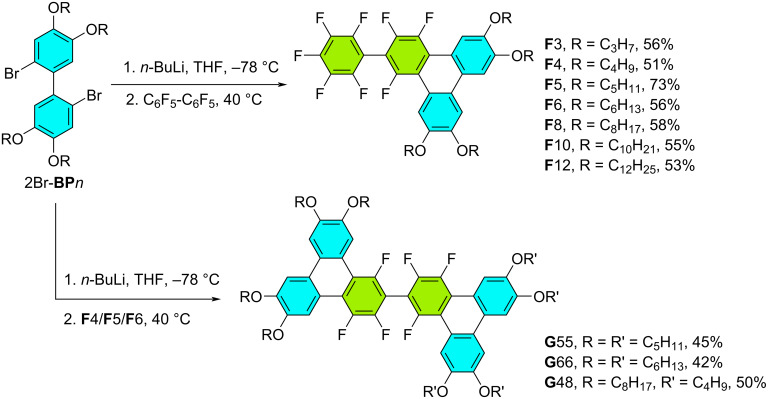
Synthesis, yields, and nomenclature of 1,2,4-trifluoro-6,7,10,11-tetraalkoxy-3-(perfluorophenyl)triphenylene (**F***n*, *n* = 3–12) and corresponding 1,1',3,3',4,4'-hexafluoro-6,6',7,7',10,10',11,11'-octakisalkoxy-2,2'-bitriphenylene dimers (**G**55, **G**66 and **G**48).

The present study focuses on synthesizing new, structurally-related series of π-conjugated aromatic compounds (Figure 1) based on a simple triphenylene core and evaluating the mesomorphic and optical properties. Specifically 1,2,4-trifluoro-6,7,10,11-tetraalkoxy-3-(perfluorophenyl)triphenylenes (**F***n*, Figure 1), bearing a terminal fluoroarene ring was obtained using the same reaction as previously reported for **PH***n* (Figure 1) [45], between 2,2’-dilithiobiphenyl derivatives but this time with the electrophile decafluorobiphenyl C_6_F_5_–C_6_F_5_ instead of C_6_H_5_–C_6_F_5_ (**F***n*, *n* = 3–12, Scheme 1). The presence of the terminal fluoroarene group in **F***n* enables to exploit further the S_N_FAr procedure, as demonstrated for 4*F*-**TP***n* and 6*F*-**BTP***n* [44], through a subsequent second annulation reaction. This results in a second series of extended π-conjugated aromatic mesomorphic compounds, 1,1',3,3',4,4'-hexafluoro-6,6',7,7',10,10',11,11'-octaalkoxy-2,2'-bitriphenylene (**G***nm*, *n,m* = 4, 5, 6, Scheme 1), with the possibility to synthesize molecules with dissymmetrical chain substitution patterns. The investigation has two main objectives. First, it seeks to explore and understand the impact of the fluorination of the pendant ring on both the self-organization and optical properties of these compounds by comparing the properties of **F***n* with those of partially fluorinated **PH***n* and non-fluorinated **BTP***n*. Such comparison is critical for optimizing materials for specific applications. Second, the presence of this terminal fluoroarene group provides a basic platform to expand this chemistry, enabling access to new π-extended molecular systems that might be challenging to synthesize through conventional synthetic routes. This dual focus highlights the study’s potential to advance both the design of functional materials and the development of innovative synthetic methodologies.

The mesomorphous properties of these two sets of compounds were investigated by polarized optical microscopy (POM), differential scanning calorimetry (DSC), and small-and wide-angle X-ray scattering (S/WAXS) and compared to related fluorinated and non-fluorinated homologs. The results showed that the Janus **F***n* derivatives exhibit a hexagonal columnar liquid crystal phase (Col_hex_), with clearing temperatures decreasing gradually with the elongation of the alkoxy side-chains, from nearly 200 °C for the shortest homologs down to ca. 100 °C for the dodecyloxy derivative. The larger lath-like compounds, **G***nm*, exhibit a rectangular columnar phase (Col_rec_) also over large temperature ranges from ambient up to 183 and 164 °C, respectively. The unsymmetrically alkoxy-substituted derivative, **G**48, also displays a Col_rec_ over a similar temperature range. The compounds’ photophysical properties have also been studied in various solutions and thin film: **G**66 emitted green light in solution with an absolute photoluminescent quantum yield of ca. 33%.

## Results and Discussion

### Synthesis and characterization

We have recently generalized a very efficient “palladium-free” synthesis for the preparation of a variety of triphenylene derivatives (Figure 1) [44–47] based on the nucleophilic substitution of various electrophilic perfluoroarenes, including C_6_F_2_H_4_, C_6_F_6_, C_10_F_8_, and C_6_F_5_-Ph, by nucleophilic organolithium reagents. e.g., 2,2’-dilithio-4,4’,5,5’-tetrakis(alkoxy)-1,1’-biphenyls, 2Li-**BP***n*, prepared in situ from the reaction between 3,3’,4,4’-tetra(alkoxy)-2,2’-dibromo-1,1’-biphenyl, 2Br-**BP***n*, and *n*-BuLi at low temperature. All new **F***n* compounds were prepared as previously described by reaction of 2Li-**BP***n* with decafluorobiphenyl, C_6_F_5_-C_6_F_5_ (Scheme 1). The starting materials 2Br-**BP***n* were prepared in high yield via FeCl_3_-oxidative coupling of 1,2-dialkoxy-4-bromobenzene (Scholl reaction). The new triphenylene derivatives, **F**3–**F**12, were prepared in moderate to high yields (51–73%). In addition, three bitriphenylene derivatives were synthesized in a subsequent annulation step from **F***n* derivatives: the in situ prepared 2Li-**BP**5/6 was reacted with **F**5/6 to yield the symmetrical discotic dimers, **G**55/**G**66 respectively, in an average yield of 42–45% (Scheme 1). The reaction of 2Li-**BP**8 with **F**4 was also successfully tested, and allowed the preparation of the unsymmetrical discotic dimer **G**48, obtained in 50% yield, opening great possibilities in structural variations. The facile synthesis of **G**55, **G**66, and **G**48 demonstrates again the versatility of this synthetic method. The synthesis and detailed characterization of compounds **F**6 and **G**66 have been the subject of a preliminary patent description. This documentation describes the methodologies employed for their preparation, the analytical techniques used to confirm their structures, and the data obtained confirming their identities [49].

Two additional compounds were synthesized to complete this study: the derivative with no alkoxy chains (**F**) was prepared to grow single crystals suitable for X-ray analysis (Scheme S2 in Supporting Information File 1) in order to confirm the annulation reaction pattern, and 2,3,6,7-tetrakishexyloxy-10-phenyltriphenylene (**BTP**6, Scheme S3), the non-fluorinated isomer of **F**6 and **PH**6 (Figure 1), for probing the effect of fluorinated rings and the decisive role of arene–fluoroarene interactions in both mesomorphism induction and stabilization. Thus, 2Li-**BP** was reacted with C_6_F_5_–C_6_F_5_ to produce 2-perfluorophenyl-1,3,4-trifluorotriphenylene (**F**), in 37% yield and 2,3,6,7-tetrakishexyloxy-10-phenyltriphenylene (**BTP**6) was synthesized via consecutive Suzuki coupling and Scholl reaction in a total yield of 77%.

The synthesis, molecular structures, nomenclature, and synthetic yields of all compounds are shown in general Scheme 1. All prepared molecules were fully characterized by NMR (^1^H, ^19^F and ^13^C), HRMS, and CHN analysis (Figures S1–S32, Supporting Information File 1), and all the results agree with the proposed molecular structures.

### Single-crystal structure of F

**Figure 2:**
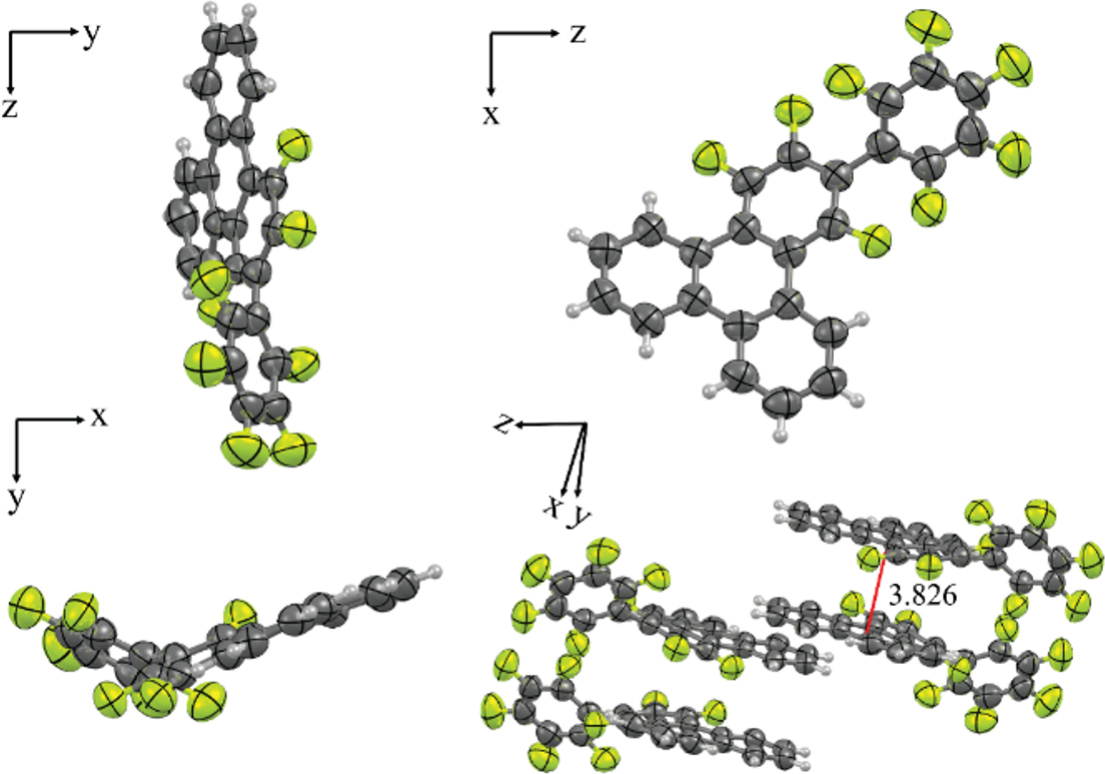
Single crystal structure of 1,2,4-trifluoro-3-(perfluorophenyl)triphenylene (**F**) viewed along the main axes: ORTEP diagram showing 50% thermal ellipsoid probability: carbon (gray), fluorine (green), and hydrogen (white).

Suitable single crystals of compound **F** for X-ray analysis were obtained by slow evaporation of an ethyl acetate/ethanol solution (Figure 2, and Supporting Information File 1, Figures S33, S34 and Tables S1–S3). The crystal structure unequivocally confirms that the reaction pattern between 2Li-**BP** and perfluorobiphenyl, effectively yielded the desired 1,2,4-trifluoro-3-(perfluorophenyl)triphenylene and that the annulation occurred at the expected positions. The similarity of the ^19^F NMR spectra of **F** and alkoxy-substituted derivatives, **F***n*, showing 6 single peaks, corresponding to the 8 different fluorine atoms at almost identical positions (Figures S8–S14 and S21 in Supporting Information File 1), confirms that the pattern of the annulation reaction is the same for all triphenylene derivatives. Thus, compound **F** crystallizes in an orthorhombic crystallographic system (*Pccn* space group, no. 56) [50] with unit cell dimensions *a* = 13.2645(2) Å, *b* = 5.5284(1) Å, and *c* = 22.7571(3) Å; the unit cell contains 8 molecules, which gives a calculated molecular density of 1.688 g cm^−3^.

In more details, the crystal structure reveals two short intramolecular F···H hydrogen contacts in the triphenylene core part with lengths of 2.047 Å and 2.114 Å, respectively (see Figure S33 in Supporting Information File 1). The triphenylene part of the molecule is rather flat, with, however, a substantial planar deviation of the pending perfluorophenyl group, forming a large tilt of ca. 45°. From the view along the *b*-axis, it can be seen that the flat triphenylene cores stack perfectly on top of each other, but with the pending fluoroarene group being alternately distributed from one side to the other, likely for steric hindrance, thus maximizing the fluoro–arene interactions by superimposing hydrogenated phenyl segments with fluoroarene ones (Figure S34 in Supporting Information File 1). Consequently, the triphenylene core–core distance is 3.83 Å and almost identical to the stacking distance in the columnar mesophase as measured by wide-angle X-ray scattering. Due to the efficient space filling and fluoroarene polar π-interaction, neighboring **F** molecules stack in an antiparallel way with a slippering distance of 1.697 Å from each other. Of course, with the presence of the lateral aliphatic chains, the cores rotate in order to maximize aliphatic–aromatic segregation whilst preserving fluoro–arene interactions.

### Liquid crystal properties

Prior to investigating the mesomorphism of **F***n* and **G***nm* compounds, their thermal stability was first examined by thermal gravimetric analysis (TGA) under a N_2_ atmosphere in the dynamic mode. The TGA curves (Figure S35 and Table S4 in Supporting Information File 1) show that all compounds undergo two thermal decomposition processes; an initial thermal event with a decomposition temperature (*T*_dec_, at weight loss 5%) between 283–332 °C for **F***n* and above 340 °C for **G***nm*, probing their excellent thermal stability.

**Figure 3:**
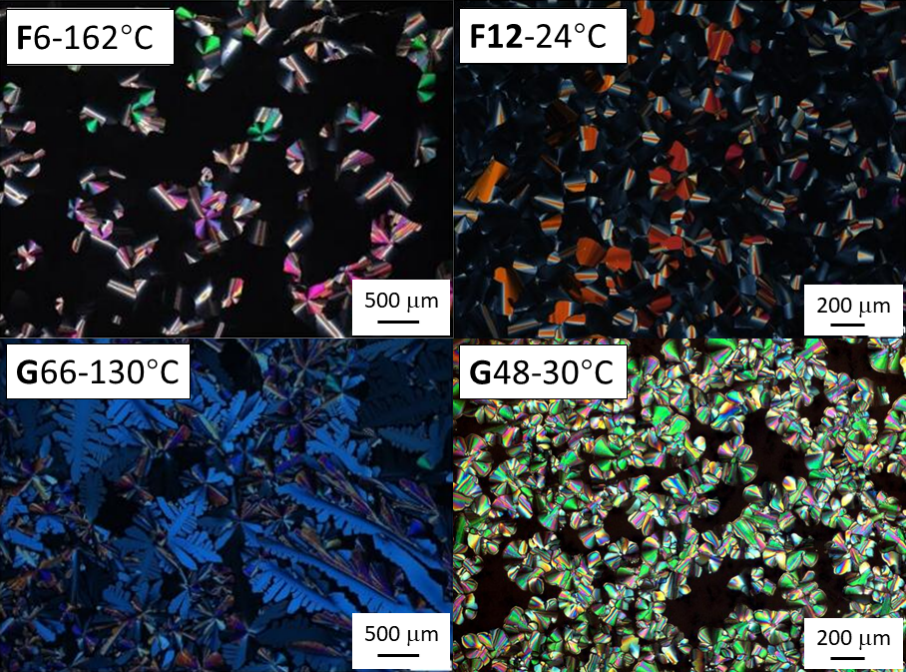
POM textures, observed between crossed polarizers of Janus and dimer, **F**6, **F**12, **G**66, and **G**48, respectively, as representative examples. More images can be seen in Supporting Information File 1 (Figure S36).

All **F***n* and **G***nm* compounds display mesophases at room temperature. Their optical textures and phase-transition behaviors were observed via hot stage polarizing optical microscopy (POM). POM images were systematically captured during the cooling process at a cooling rate of 1 °C/min after the compounds were heated into the isotropic liquid (Figure 3 and Figure S36 in Supporting Information File 1). The liquid crystalline phases of the Janus triphenylenes, **F**3 to **F**12, all showed optical textures of a hexagonal columnar mesophase. Slowly cooled from its isotropic liquid state, **F**6 grew up into broken, fan-shaped color plates among a large dark area, with straight line defects, characteristic of the hexagonal columnar mesophase. The discotic dimers **G***nm* displayed a dense optical texture with dendritic- and flower-like features, with small fraction of dark area, which could be possibly attributed to a reduction of the mesophase symmetry.

**Figure 4:**
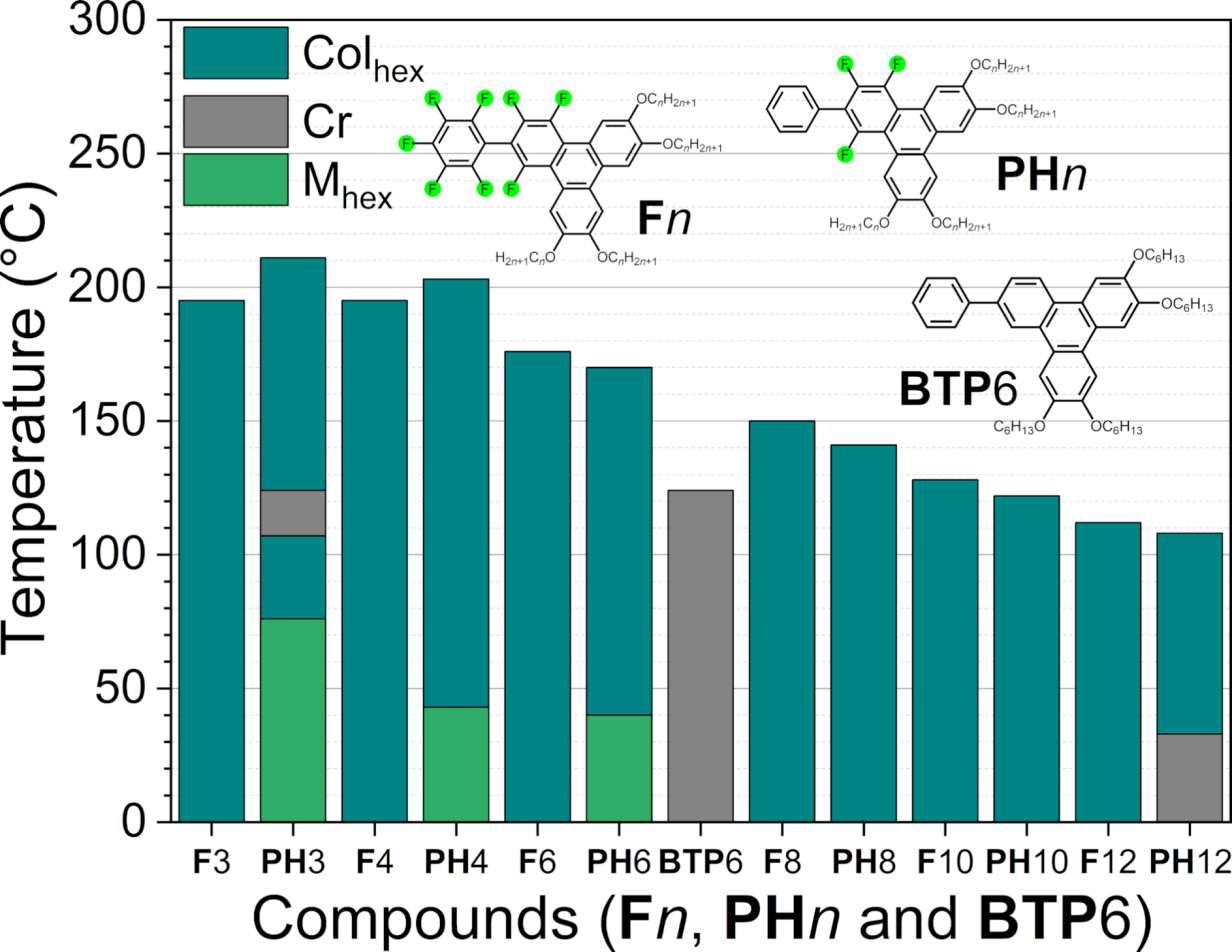
Comparative bar graph summarizing the thermal behavior of **F***n*, **BTP**6, and **PH***n* derivatives (2nd heating DSC data).

The results of the differential scanning calorimetry (DSC) are summarized in Figure 4 (see also Supporting Information File 1, Figures S37, S38 and Table S5 for details), confirming the POM observations and their room temperature behaviors (no crystallization is observed even at low temperature, except for **F**12). Compounds with shorter alkyl chains, **F**3, **F**4, and **F**5, possess almost the same clearing temperatures near 190 °C, whereas from **F**6 to **F**12, the clearing temperature gradually lowers from 176 down to 112 °C. When comparing **F***n* and **PH***n* (Figure 4) [45], both types of compounds show a high-range columnar mesophase at high temperature with almost identical clearing temperatures, which decrease gradually with the elongation of the alkoxy side-chains. The only difference is the emergence of a more ordered, 3D phase for some **PH***n* derivatives observed on cooling at lower temperature. As expected, neither **F** or **BTP**6 are mesomorphic.

As for the larger fluorine derivatives **G***nm* (**G**55, **G**66, and **G**48), they all possess enantiotropic columnar mesophases right from room temperature up to 183 (**G**55), 164 (**G**66), and ca. 120 °C (**G**48). Compared to the previously synthesized non-fluorous pentyloxy homolog, showing a monotropic Col_rec_ phase [51–52], core fluorination surely plays a positive role in the induction of mesomorphism.

**Figure 5:**
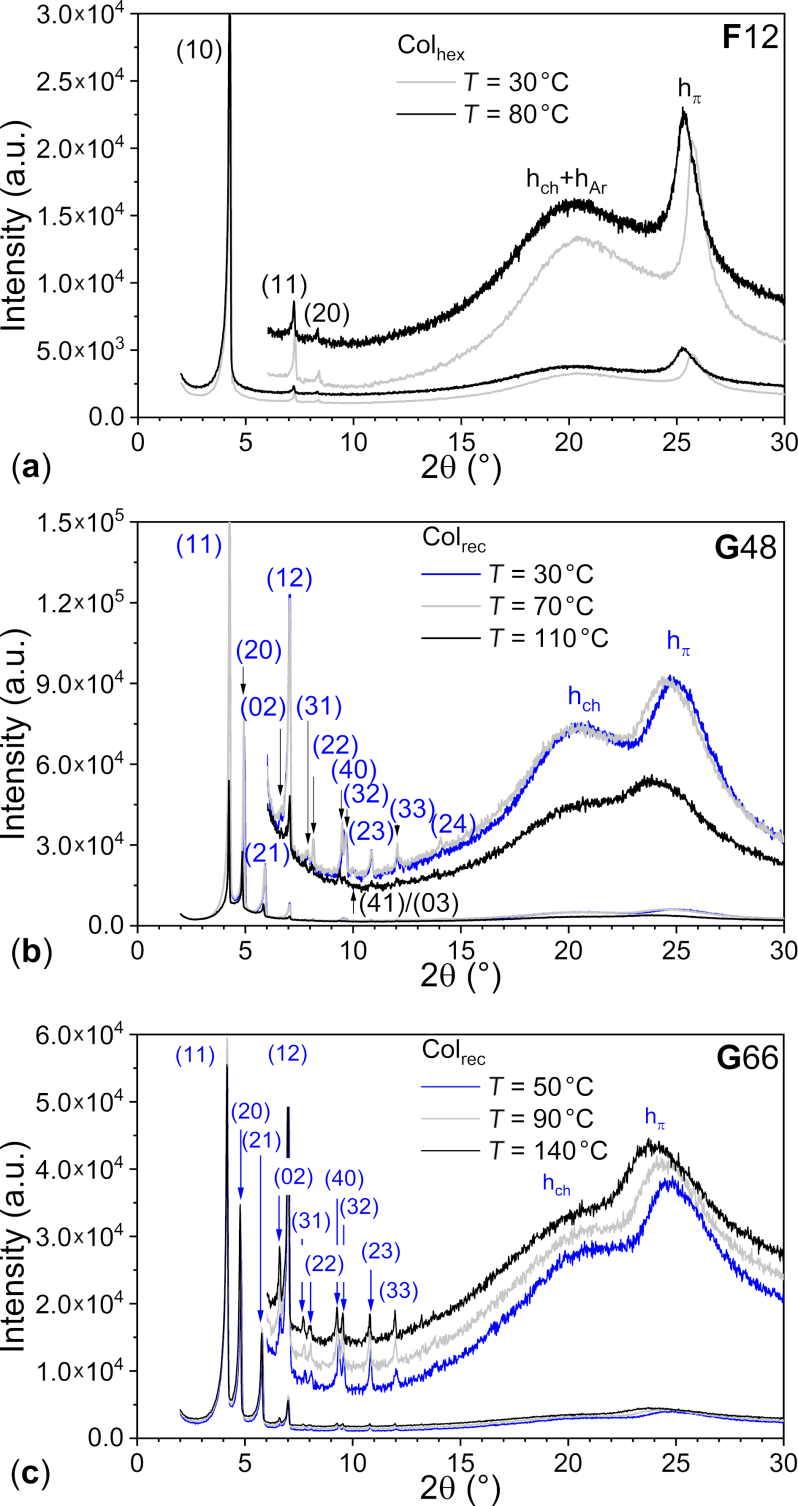
Representative S/WAXS patterns of **F***n* and **G***nm* compounds.

The mesophases of **F***n* and **G***nm* compounds were fully characterized by small- and wide-angle X-ray scattering (S/WAXS) at several temperatures (Figure 5, Figures S39–S41 and Table S6 in Supporting Information File 1). The X-ray patterns of the **F***n* compounds exhibit one main, sharp, and intense reflection in the small-angle range, and an additional small peak indexed as (20) for **F**3 and **F**4, or two peaks indexed as (11) and (20), respectively, for **F**10 and **F**12, confirming unambiguously the hexagonal symmetry (**F**5, **F**6, and **F**8 only show the intense small-angle reflection). In addition, they all show a broad diffuse scattering and a sharp and intense diffraction peak in the wide-angle region, assigned respectively to as h_ch_, for the disordered chains, and h_π_, for the long-range core–core stacking resulting from strong polar–π interactions. The correlation length of the stacking was calculated by the Debye–Scherrer formula, which correspond to ca. 15–25 stacked molecules (Table 1). This coincides with the high clearing temperatures and high stability of the columnar mesophase of **F***n*. Overall, the behaviors of **F***n* and **PH***n* compounds are very similar, with only minor deviations of the isotropic temperatures.

**Table 1:** Mesophases’ parameters.

Cpds	Phase	Temp.^a^	*a*^b^/*b*^b^	*A* ^b^	*h*_π_(ξ*)/h*_ch_^c^	*V*_mol_^d^/ρ^d^	*h*_mol_^e^

**F**3	Col_hex_	120	16.73	242.44	3.53 (67)/4.41	895/1.26	3.69
**F**3	Col_hex_	180	16.71	241.77	3.64 (56)/4.40	938/1.20	3.88
**F**4	Col_hex_	50	17.57	267.38	3.45 (64)/4.42	968/1.26	3.62
**F**4	Col_hex_	120	17.74	272.60	3.55 (65)/4.31	1010/1.21	3.71
**F**5	Col_hex_	40	18.46	295.14	3.44 (79)/4.44	1074/1.22	3.64
**F**5	Col_hex_	100	18.52	297.08	3.52 (83)/4.38	1111/1.18	3.74
**F**5	Col_hex_	160	18.57	298.57	3.62 (59)/4.37	1161/1.13	3.89
**F**6	Col_hex_	70	19.31	322.78	3.48 (72)/4.47	1203/1.17	3.73
**F**6	Col_hex_	150	19.56	331.25	3.60 (47)/4.58	1270/1.11	3.83
**F**8	Col_hex_	50	21.43	397.59	3.46 (79)/4.30	1410/1.13	3.55
**F**8	Col_hex_	130	21.46	398.76	3.58 (42)/4.40	1485/1.07	3.72
**F**10	Col_hex_	30	22.79	450.03 Å^2^	3.45 (78)/4.37	1612/1.10	3.58
**F**10	Col_hex_	100	23.09	461.88 Å^2^	3.54 (42)/4.36	1629/1.09	3.64
**F**12	Col_hex_	30	24.30	511.40 Å^2^	3.45 (75)/4.38	1829/1.08	3.58
**F**12	Col_hex_	80	24.47	518.53 Å^2^	3.51 (47)/4.40	1886/1.04	3.64

**G**55	Col_rec_	40	35.20/25.46	896.19 Å^2^	3.57 (–)/4.28	1778/1.17	4.04
**G**55	Col_rec_	100	35.26/25.55	900.89 Å^2^	3.68 (–)/4.45	1853/1.12	4.11
**G**55	Col_rec_	160	35.32/25.74	909.00 Å^2^	3.72 (–)/4.51	1952/1.06	4.31
**G**66	Col_rec_	50	37.18/26.80	996.42 Å^2^	3.56 (–)/4.36	2009/1.13	4.03
**G**66	Col_rec_	90	37.32/26.65	994.58 Å^2^	3.66 (–)/4.48	2066/1.10	4.15
**G**66	Col_rec_	140	37.46/26.53	993.81 Å^2^	3.75 (–)/4.50	2152/1.05	4.33
**G**48	Col_rec_	30	36.78/26.61	978.71 Å^2^	3.54 (67)/4.39	1985/1.14	4.06
**G**48	Col_rec_	70	36.98/26.54	981.45 Å^2^	3.59 (–)/4.45	2036/1.11	4.15
**G**48	Col_rec_	110	37.14/26.31	977.15 Å^2^	3.66 (–)/4.39	2098/1.08	4.29

^a^Temperature of experiment (°C); ^b^lattice parameter, *a*/*b* (Å) and area, *A* (Å^2^), *A* = *a*^2^√3/2 (for Col_hex_) = *a* × *b* ( for Col_rec_), *N*_col_: number of columns per lattice: *N*_col_ = 1 for Col_hex_, *N*_col_ = 2 for Col_rec_; ^c^average face-to-face stacking distance (Å) between consecutive mesogens, h_π_, determined from scattering maximum from SWAXS pattern, and ξ, correlation length (Å) calculated by Debye–Scherrer formula; *h*_ch_, average distance (Å) between molten chains; ^d^molecular volume (Å^3^) and density (g·cm^−3^) calculated from partial volumes of reference substances: *V*_mol_ = *V*_ar_ + *V*_ch_, the sum of the volume of the aromatic part, *V*_ar_ (from reference compounds) and the volume of the chains, *V*_ch_ [53], ρ = MW/(N_A_·*V*_mol_); ^e^columnar slice thickness (Å), *h*_mol_ = N_col_*V*_mol_/A (Å).

The single structure shows that the flat triphenylene cores almost stack perfectly on top of each other, with an alternation of the pending fluorinated phenyl groups by superimposition of one of the hydrogenated rings of the triphenylene segment above the trifluoroarene one, in order to maximize fluoro–arene intermolecular interactions between the molecules (Figure S34 in Supporting Information File 1). It also shows that the pending fluorophenyl makes an angle of ca. 45° with the triphenylene plane (see also DFT below). Despite this conformational distortion, the molecular thickness, *h*_mol_, obtained by dividing the molecular volume with the columnar cross section, is not drastically increased (Table 1); *h*_mol_ ranges between 3.62 and 3.73 Å, very close to the stacking distance between consecutive cores measured by S/WAXS (3.42 Å ≤ *h*_π_ ≤ 3.62 Å), confirming that the triphenylene mesogens pile up in the columns with no or little tilt, reminiscent of the stacking observed in the crystal structure. It would thus consist of the piling of the triphenylene cores with the protruding fluorophenyl segments partly mixed with the aliphatic medium, with specific orientations of the triphenylenes (multiple of 120° orientations which contribute to the average circular cross-section of the columns) in order to maximize both the intermolecular interactions through the superimposition of fluoroarene segments and (alkylated) arene ones, partly similar to the crystal structure, and the homogeneous distribution of the chains around the columns, in agreement with the hexagonal symmetry. Both sets **F***n* and **PH***n* show rather similar variation of the cross-sectional area, increasing homogeneously with *n*, as well as similar molecular thickness throughout (*A* and *h*_mol_, Figure S41 in Supporting Information File 1). Consequently, both the packing of **F***n* and of **PH***n* in the Col_hex_ phase must be very similar, with no effect of the pending group nature (C_6_H_5_ vs C_6_F_5_) on the mesophase stability. A proposed model for the supramolecular organization of **F***n* in the Col_hex_ phase is shown in Figure S42 of Supporting Information File 1.

The bitriphenylene **G***nm* materials exhibit different X-ray patterns, with a multitude of sharp peaks, that could be indexed according to a rectangular lattice (*p*2*gg* symmetry) [50], confirming the reduction of the phase symmetry and well-defined interfaces between aliphatic continuum, hydrogenated aromatics, and fluorinated arenes. The S/WAXS patterns of the three compounds are also identical, independently of the chain length or the chain distribution, and the lattice expansion with temperature is not significative. However, the stacking appears to be less effective than for the Janus derivatives as the signal corresponding to core–core stacking is not as sharp and intense, corresponding to a decrease of fluoroarene–arene interactions. This agrees with an increase of the molecular thickness (see *h*_mol_ values, Table 1), likely due to some electrostatic and steric repulsion between fluorine atoms within the inner core. DFT shows that both triphenylene segments do not lie in the same plane and that the overall molecule is slightly twisted. Nevertheless, the molecules still stack on top of each other in columns, maintaining the segregation between the various regions as in polyphilic molecules [9–10,54] and, since the molecules have a more anisotropic shape, the columnar cross-section cannot adopt a circular shape but rather an elliptical one, hence their arrangement into a rectangular lattice (Figure S43 in Supporting Information File 1). For **G***nm*, the mesomorphism is thus essentially driven by microsegregation between the various molecular constituents.

### Photophysical properties: UV–vis absorption and photoluminescence

**Figure 6:**
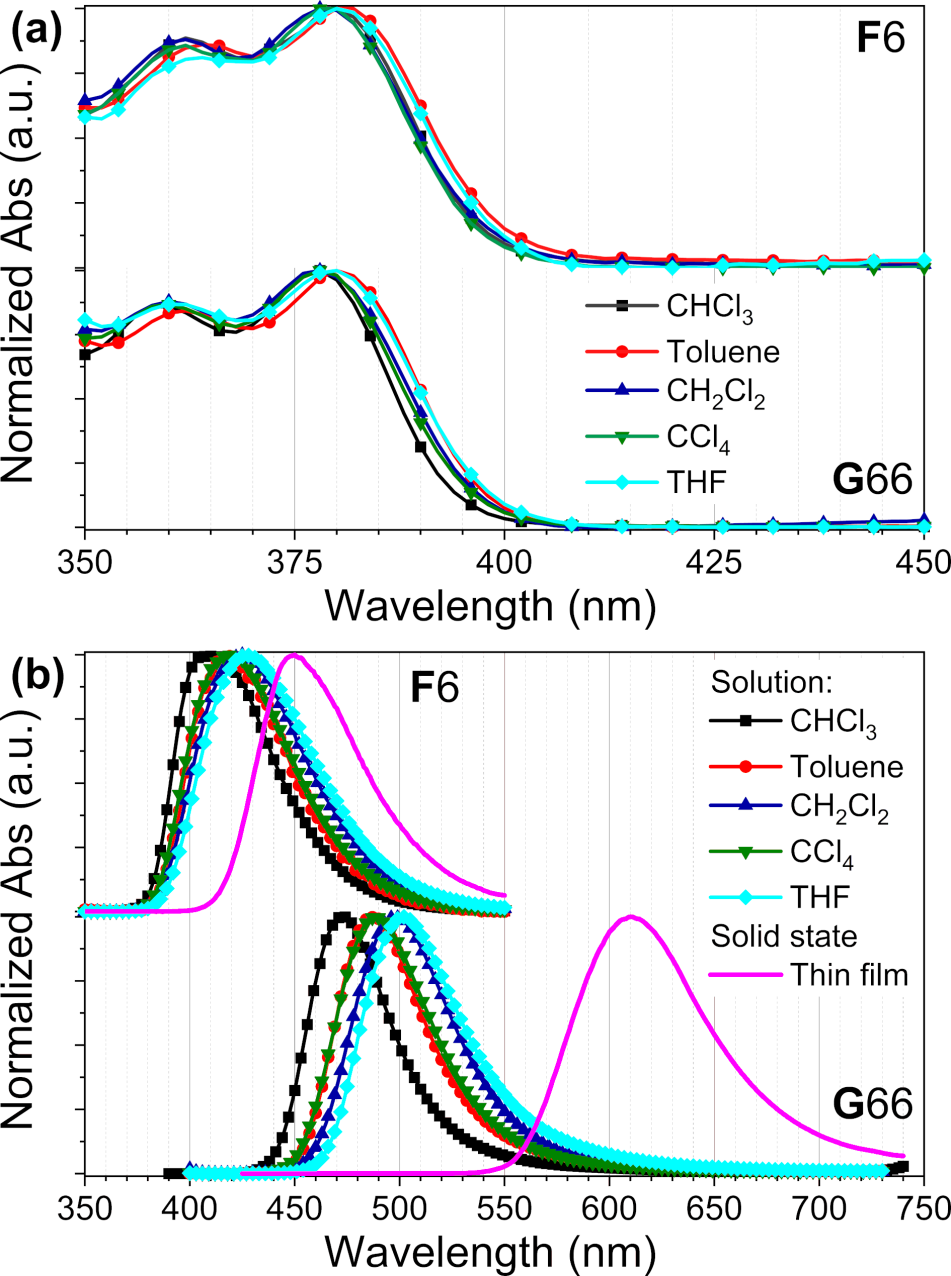
Absorption (a) and emission (b) spectra of **F**6 and **G**66, measured in different solvents at a concentration of 1 × 10^−5^ mol/L and in solid-state thin film.

UV–vis absorption and fluorescence spectra of the synthesized fluorine polycyclic aromatic hydrocarbons **F***n* and **G***nm* were measured in solution (in various solvents) and thin film and the results are summarized in Figure 6 and Table S7 in Supporting Information File 1; **F**6 and **G**66 were chosen as representative examples.

**F**6 and **G**66 show almost identical UV–vis absorption spectra, both of them possessing a very broad absorption band below 400 nm with two maxima at ca. 380 nm for the strongest peak, and at ca. 360 nm for the smaller one (λ_abs_ = 284 nm for **PH**8), with no or little influence of the solvent polarity (Figure 6a). This may suggest that the sigma-bonded **F**6 and **G**66 have no conjugation in their ground states, the electron density being located on one triphenylene moiety (see DFT). **G**66 shows a stronger absorption band than **F**6, with expected ε values in the range of 10^4^ and 10^5^ L·mol^−1^·cm^−1^, respectively, as **G**66 possesses two triphenylene units whereas **F**6 only one.

Both compounds, however, display substantially different photoluminescent behavior. Both **F**6 and **G**66 show a single, broad emission band with a peak maximum around 410–430 nm for **F**6 (λ_em_ = 402 nm for **PH**8) and 460–500 nm for **G**66, with absolute quantum yields of 30% and 32%, respectively. Further, their fluorescence spectra in solutions show some solvent polarity influence, with a substantial red shift as the solvent polarity is increased. In thin film, the single luminescence maximum is shifted to 450 nm (518 nm) and 610 nm for **F**6 (**PH**8) and **G**66, respectively. The thin film emission of **G**66 is red-shifted by about 120 nm compared with its solution, while the one of **F**6 is red-shifted only by 30 nm. This huge difference in thin film emission can be explained via their intermolecular slipped *J*-type aggregation, supported by S/WAXS and proposed the model of Col_rec_ mesophase [55–57].

### DFT computation

**Figure 7:**
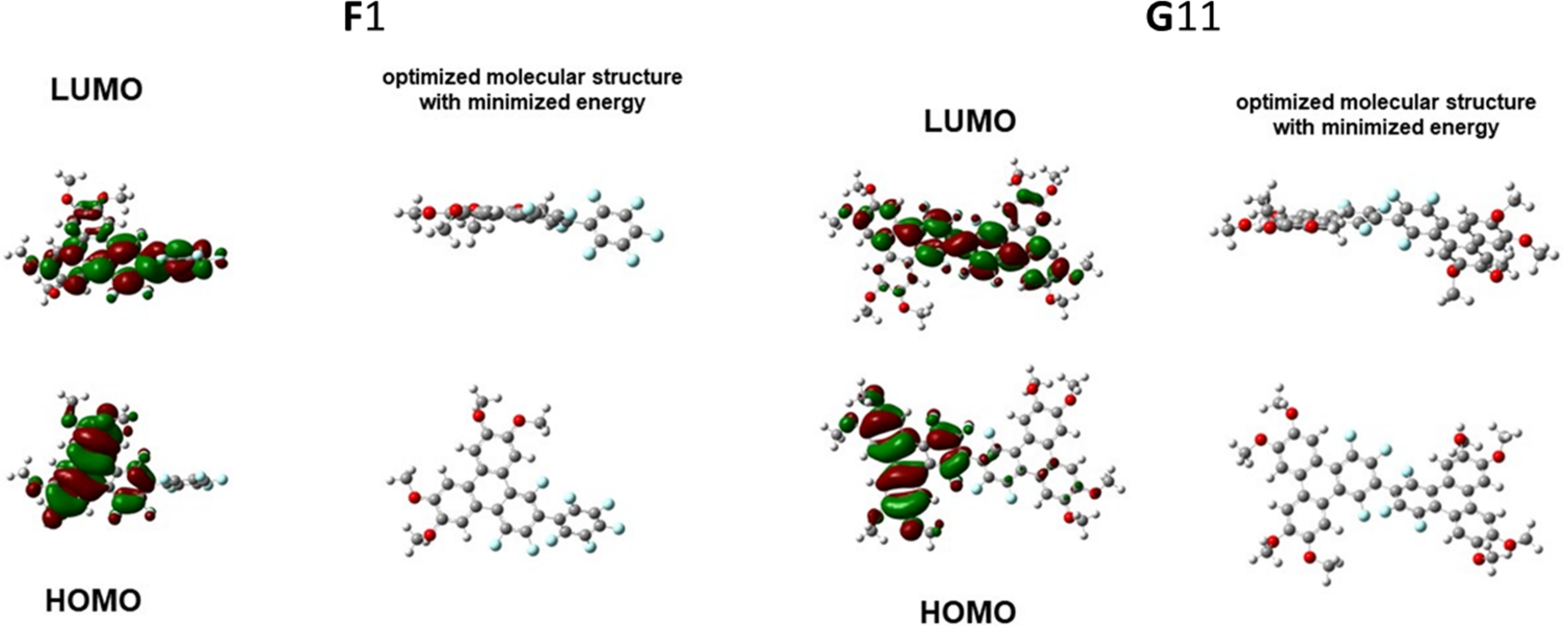
DFT calculated frontier molecular orbitals and optimized molecular structures for **F**1 and **G**11.

For a deeper understanding of the electronic properties of these fluorine triphenylenes, we performed some DFT computation of **F**1 and **G**11 with the shortest methyl chain, and the results are summarized in Figure 7 and Figure S44 in Supporting Information File 1. First, the theoretically optimized molecular structure of **F**1 agrees well with the single crystal structure of **F**: the triphenylene core is planar and the side arm perfluorophenyl group is rotated by a few degrees due to the F···F repulsion on different rings. **G**11 exhibits a similar twist between the two triphenylene cores. Further, the calculated HOMO electron cloud of **F**1 (−5.8 eV; −5.89 eV for **PH**1) and **G**11 (−5.60 eV) both are located on a triphenylene core, which explains the similarity of their UV–vis absorption spectra. Their LUMO electron density maps distribute across to the side arm for **F**1 (−1.88 eV; −1.78 eV for **PH**1) and to the other triphenylene core for the dimer **G**11 (−1.78 eV). The π-conjugation of excited states results in a difference of their HOMO and LUMO energy levels: the fluorine dimer **G**11 possesses higher HOMO and LUMO energy levels than that of the monomer **F**1, with a smaller HOMO–LUMO energy gap (3.92 eV for **F**1 versus 4.11 eV for **PH**1). The DFT results thus agree pretty well with the fluorescence spectra in solution: **G**66 shows a fluorescence peak at 470–500 nm, while **F**6 has peaks located between 410–430 nm. It is noted that **G**66 shows deeper red-shifted fluorescence than **F**6, with peaks of 610 nm and 450 nm, respectively. The *J*-type aggregation for **G**66 in thin film and liquid crystalline state is energetically favorable for the arene–perfluoroarene overlap stacking and related stereoelectronic effects, which results in more than 100 nm red-shift of the fluorescence peak.

## Conclusion

We have successfully prepared seven fluorine triphenylenes (**F**3 to **F**12) with various alkyl chain lengths and three bitriphenylene dimers (**G**55, **G**66, and **G**48) with different molecular symmetry by the S_N_Ar reaction. This “palladium-free” reaction between 2Li-**BP***n* and C_6_F_5_–C_6_F_5_ possesses several advantages: easily available starting chemicals, low cost, efficient, and versatile, displaying the potential to synthesize more complicated fluoroarene molecules and polymers. These fluorine-containing triphenylenes **F***n* and dimers **G***nm* display Col_hex_ and Col_rec_ mesophases, respectively, with high stability of the columnar mesophases due to strong arene–perfluoroarene polar π-interactions and related stereoelectronic effects. These Janus-type **F***n* compounds exhibit high clearing temperatures, and no crystalline phase, thus a very broad columnar mesophase range. Further, the sigma-bonded triphenylene dimers **G***nm* display an enantiotropic Col_rec_ mesophase including room temperature. These π-conjugated materials are advantageous for further investigation in device applications. The aromatic core fluorination changes the electronic structures of the triphenylenes, and their supramolecular arene–perfluoroarene slipped stacks (*J*-aggregate) result in **G**66 with orange-yellow color fluorescence in the solid state.

## Supporting Information

Synthesis (Schemes S1–S3) and characterization, ^1^H, ^13^C, and ^19^F NMR (Figures S1–S21), HRMS (Figures S22–S32), EA, single crystal X-ray structures (Figures S33, S34, Tables S1–S3), TGA (Figure S35, Table S4), POM (Figure S36), DSC (Figures S37, S38, Table S5), S/WAXS (Figures S39–S43, Table S6), optical properties (Table S7), and DFT (Figure S44, Table S8).

File 1 Experimental part.

## Data Availability

All data that supports the findings of this study is available in the published article and/or the supporting information of this article.
