# Supplementary material for: Efficient synthesis of fluorinated triphenylenes with enhanced arene–perfluoroarene interactions in columnar mesophases
Source: Beilstein J Org Chem. 2024 Dec 16;20:3263–73. doi: 10.3762/bjoc.20.270 (PMC11665444; doi:10.3762/bjoc.20.270)
Supplement: File 1 — Experimental part. [file Beilstein_J_Org_Chem-20-3263-s001.pdf]

BEILSTEIN JOURNAL OF ORGANIC CHEMISTRY

Supporting Information

for

Efficient synthesis of fluorinated triphenylenes with enhanced arene–perfluoroarene interactions in columnar mesophases

Yang Chen, Jiao He, Hang Lin, Hai-Feng Wang, Ping Hu, Bi-Qin Wang, Ke-Qing Zhao and Bertrand Donnio

Beilstein J. Org. Chem. **2024,** *20,* 3263–3273. doi:10.3762/bjoc.20.270

Experimental part

License and Terms: This is a supporting information file under the terms of the Creative Commons Attribution License (https://creativecommons.org/licenses/by/4.0). Please note that the reuse, redistribution and reproduction in particular requires that the author(s) and source are credited and that individual graphics may be subject to special legal provisions.
The license is subject to the *Beilstein Journal of Organic Chemistry* terms and conditions: (https://www.beilstein-journals.org/bjoc/terms)

Entry	Table of contents	Page No.
1	Materials and methods	S2
2	Synthesis and characterization	S3–S10
3	^{1}H NMR, ^{19}F NMR and ^{13}C NMR spectra	S10–S32
4	HRMS spectra	S33–S43
5	Single-crystal structural analysis	S43–S47
6	TGA	S47
7	POM	S48–S49
8	DSC	S50–S52
9	S/WAXS	S53–S61
10	Photophysical properties	S61
11	DFT	S62

1. Materials and Methods

Chemicals. All commercially available starting materials were used directly without further purification. The solvents of air- and moisture-sensitive reactions were carefully distilled from appropriate drying agents before use.

Experimental. Air- and moisture-sensitive reactions were assembled on a Schlenk vacuum line or in a glovebox using oven-dried glassware with a Teflon screw cap under Ar atmosphere. Air- and moisture-sensitive liquids and solutions were transferred by syringe. Reactions were stirred using Teflon-coated magnetic stirring bars. Elevated temperatures were maintained using Thermostat-controlled air baths. Organic solutions were concentrated using a rotary evaporator with a diaphragm vacuum pump.

Analytical. ^{1}H NMR/^{19}F NMR/^{13}C NMR spectra were recorded using a Varian UNITY INOVA 400/100 MHz or Bruker 600 MHz spectrometer in $CDCl_3$, and TMS as the internal standard. High-resolution mass spectra (HRMS) were recorded with a Bruker Fourier transform high resolution mass spectrometer (solariX XR) with MALDI as the ion source. Elemental analyses (EA) were performed on a Vario Micro Select (Elementar company, German). The thermal gravimetrical analysis (TGA) was measured on a TA-TGA Q500 instrument with heating rate of 20 °C/min in N_2 atmosphere. The phase-transition temperatures and enthalpy changes were investigated using a TA-DSC Q100 differential scanning calorimeter (DSC) under N_2 atmosphere with heating or cooling rate of 10 °C/min. Liquid crystalline optical textures were observed and recorded on an Olympus BH2 Polarized Optical Microscope (POM) equipped with a Mettler FP82HT hot-stage by which temperatures were controlled by a XPR-201 and Mettler FP90. Temperature-variation SAXS (small-angle X-ray scattering) and WAXS (wide-angle X-ray scattering) experiments on Rigaku Smartlab X-Ray Diffractometer with TCU 110 temperature controller, while the sample temperature was controlled within ±1 K and the X-ray sources (Cu Kα, λ =0.154 nm) were provided by 40 kW ceramic tubes.

UV/Vis. Absorption spectra were recorded on a Perkin Elmer Lambda 950 spectrophotometer at room temperature. Fluorescence was measured on a HORIBA Fluoromax-4p, and the quantum yields were measured by a HORIB-F-3029 Integrating Sphere, Horiba, Kyoto, Japan. A suitable crystal was selected on a XtaLab Synergy R, DW system, HyPix diffractometer. The crystal was kept at 300.2(7) K during data collection. Using Olex2, the structure was solved with the ShelXT structure solution program using Intrinsic Phasing and refined with the ShelXL refinement package using Least Squares minimisation.

For DFT computation, the B3LYP-D3 method was used, with selected basis set of 6-311g(d,p). The energetically favorable molecular conformation in gas phase obtained via optimizing geometrical molecular structures and frequency calculation.

2. Synthesis and characterization

The synthesis of 4-bromobenzene-1,2-diol, [1] 4-bromo-1,2-bis(alkoxy)benzene (Br-**P***n*),[1] 2-bromo-3',4,4',5-tetrakis(hexyloxy)-1,1'-biphenyl (Br-**BP**6) [2] and 2,2'-dibromo-4,4',5,5'-tetra(alkoxy)-1,1'-biphenyl (2Br-**BP***n*)[3] were performed according to reported methods. All other materials were used as purchased without further purification.

2.1 Synthesis of the 2,2'-dibromo-4,4',5,5'-tetra(alkoxy)-1,1'-biphenyl (2Br-BP*n*).

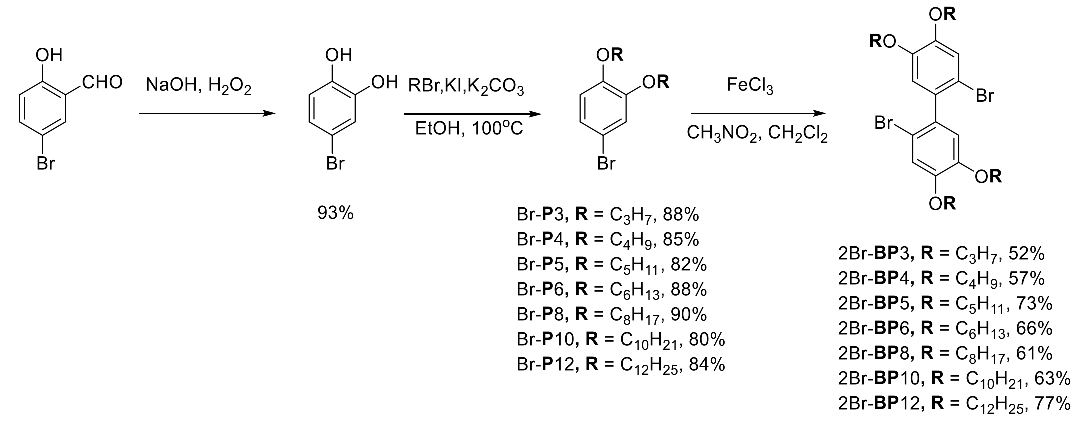

Scheme S1. Synthesis of the 2,2'-dibromo-4,4',5,5'-tetra(alkoxy)-1,1'-biphenyl (2Br-**BP***n*).

4-Bromobenzene-1,2-diol: Into a solution of NaOH (17.30 g, 0.43 mol) in H_2O (220 mL) was added 5-bromosalicylaldehyde (80.00 g, 0.40 mol), and the mixture heated at 60 °C until all reactants dissolved. The mixture was cooled by an ice–water bath, and 30% of H_2O_2 (51 mL) was added slowly with a constant-pressure dropping funnel. The resulting solution was stirred at room temperature for 2 h. A saturated solution of NaCl was added to the reaction mixture, and the product was extracted with Et_2O. The combined organic layers were dried over anhydrous $MgSO_4$ and concentrated in vacuum to give 4-bromobenzene-1,2-diol as yellow oil (9.80 g, 93%).

General procedure for the synthesis of 4-bromo-1,2-bis(alkoxy)benzene (Br-**P***n*): 4-bromocatechol (18.52 mmol, 1.0 equiv), potassium carbonate (55.56 mmol, 3.0 equiv), and a pinch of KI were weighed into a round-bottomed flask. Subsequently, EtOH (70 mL) and 1-bromoalkane (40.74 mmol, 2.2 equiv) were added and the resulting solution was stirred at 100 °C for 24 h. Then, the mixture was cooled and extracted with dichloromethane. The organic phase was dried over anhydrous $MgSO_4$, filtered and spin-dried. Purification by silica gel column chromatography (dichloromethane/petroleum ether 1:3, v/v) afforded Br-**P***n* as yellow liquid in yields of 80–90%.

General procedure for the synthesis of 2,2'-dibromo-4,4',5,5'-tetra(alkoxy)-1,1'-biphenyl (2Br-**BP***n*): A solution of 4-bromo-1,2-bis(alkoxy)benzene (Br-**P***n*, 1 equiv) in CH_2Cl_2 (40 mL) was placed in a 100 mL round-bottomed flask and a solution of $FeCl_3$ (2 equiv) in CH_3NO_2 (5 mL) was added. The resulting

[1] K. Q. Zhao, Y. Gao, W. H. Yu, P. Hu, B. Q. Wang, B. Heinrich and B. Donnio, Discogens possessing aryl side groups synthesized by Suzuki coupling of triphenylene triflates and their self-organization behavior, *Eur. J. Org. Chem.*, **2016**, 2802-2814.
[2] J. F. Hang, H. Lin, K. Q. Zhao, P. Hu, B. Q. Wang, H. Monobe, C. H. Zhu and B. Donnio, Butterfly mesogens based on carbazole, fluorene or fluorenone: mesomorphous, gelling, photophysical, and photoconductive properties, *Eur. J. Org. Chem.*, **2021**, 2021, 1989-2002.
[3] a) H.-M. Pan, J. He, W.-H. Yu, P. Hu, B.-Q. Wang, K.-Q Zhao, B. Donnio, 2-Aryl-1,3,4-trifluoro-6,7,10,11-tetrakis(alkoxy)triphenylene: a remarkable and highly inclusive mesomorphic platform, *J. Mater. Chem.*, **2023**, 11, 14695-14704; b) M.-M. Zhou, J. He, H.-M. Pan, Q. Zeng, H. Lin, K.-Q. Zhao, P. Hu, B.-Q. Wang, B. Donnio, Induction and stabilization of columnar mesophases in fluorinated polycyclic aromatic hydrocarbons by arene-perfluoroarene interactions, *Chem. Eur. J.*, **2023**, *29*, e202301829.

solution was stirred at room temperature until completion of the reaction. The reaction was quenched with methanol and extracted with dichloromethane. The organic phase was dried over anhydrous $MgSO_4$, filtered, and spin-dried. Purification by silica gel column chromatography (dichloromethane/petroleum ether 1:3, v/v) and recrystallization from ethanol and methanol/ethyl acetate gave 2Br-**BP***n* as a white solid in yields of 52–77%.

2,2'-Dibromo-4,4',5,5'-tetrapropoxy-1,1'-biphenyl (2Br-**BP**3): Br-**P**3 (3.1 g, 11.3 mmol), CH_2Cl_2 (40 mL), $FeCl_3$ (3.7 g, 22.6 mmol), CH_3NO_2 (5 mL). Purification by silica gel column chromatography (dichloromethane/petroleum ether = 1:3 v/v), recrystallized in methanol and ethanol to give a white solid 2Br-**BP**3 (1.6 g, 52%). **^{1}H NMR** ($CDCl_3$, TMS, 400 MHz) δ: 7.10 (s, 2H, ArH), 6.76 (s, 2H, ArH), 4.00-3.92 (m, 8H, OCH_2), 1.91-1.78 (m, 8H, CH_2), 1.08-1.00 (m, 12H, CH_3).

2,2'-Dibromo-4,4',5,5'-tetrabutoxy-1,1'-biphenyl (2Br-**BP**4): Br-**P**4 (4.8 g, 15.9 mmol), CH_2Cl_2 (40 mL), $FeCl_3$ (5.2 g, 31.8 mmol), CH_3NO_2 (5 mL). Purification by silica gel column chromatography (dichloromethane/petroleum ether = 1:3 v/v), recrystallized in methanol and ethanol to give a white solid 2Br-**BP**4 (2.7 g, 57%). **^{1}H NMR** ($CDCl_3$, TMS, 400 MHz) δ: 7.10 (s, 2H, ArH), 6.76 (s, 2H, ArH), 4.03-3.95 (m, 8H, OCH_2), 1.86-1.75 (m, 8H, CH_2), 1.55-1.45 (m, 8H, CH_2), 1.01-0.94 (m, 12H, CH_3).

2,2'-Dibromo-4,4',5,5'-tetrapentyloxy-1,1'-biphenyl (2Br-**BP**5): Br-**P**5 (5.2 g, 15.8 mmol), CH_2Cl_2 (40 mL), $FeCl_3$ (5.1 g, 31.6 mmol), CH_3NO_2 (5 mL). Purification by silica gel column chromatography (dichloromethane/petroleum ether = 1:3 v/v), recrystallized in ethyl acetate and ethanol to give a white solid 2Br-**BP**5 (3.8 g, 73%). **^{1}H NMR** ($CDCl_3$, TMS, 400 MHz) δ: 7.09 (s, 2H, ArH), 6.76 (s, 2H, ArH), 4.02-3.94 (m, 8H, OCH_2), 1.88-1.77 (m, 8H, CH_2), 1.48-1.34 (m, 16H, CH_2), 0.96-0.90 (m, 12H, CH_3).

2,2'-Dibromo-4,4',5,5'-tetrahexyloxy-1,1'-biphenyl (2Br-**BP**6): Br-**P**6 (8.3 g, 23.2 mmol), CH_2Cl_2 (40 mL), $FeCl_3$ (7.5 g, 46.4 mmol), CH_3NO_2 (5 mL). Purification by silica gel column chromatography (dichloromethane/petroleum ether = 1:4 v/v), recrystallized in ethyl acetate and ethanol to give a white solid 2Br-**BP**6(5.5 g, 66%). **^{1}H NMR** ($CDCl_3$, TMS, 400 MHz) δ: 7.09 (s, 2H, ArH), 6.75 (s, 2H, ArH), 4.02-3.94 (m, 8H, OCH_2), 1.87-1.76 (m, 8H, CH_2), 1.50-1.31 (m, 24H, CH_2), 0.93-0.87 (m, 12H, CH_3).

2,2'-Dibromo-4,4',5,5'-tetraoctyloxy-1,1'-biphenyl (2Br-**BP**8): Br-**P**8 (6.9 g, 16.7 mmol), CH_2Cl_2 (40 mL), $FeCl_3$ (5.4 g, 33.4 mmol), CH_3NO_2 (5 mL). Purification by silica gel column chromatography (dichloromethane/petroleum ether = 1:4 v/v), recrystallized in ethyl acetate and ethanol to give a white solid 2Br-**BP**8 (4.2 g, 61%). **^{1}H NMR** ($CDCl_3$, TMS, 400 MHz) δ: 7.09 (s, 2H, ArH), 6.76 (s, 2H, ArH), 4.02-3.94 (m, 8H, OCH_2), 1.87-1.76 (m, 8H, CH_2), 1.52-1.27 (m, 40H, CH_2), 0.91-0.86 (m, 12H, CH_3).

2,2'-Dibromo-4,4',5,5'-tetradecyloxy-1,1'-biphenyl (2Br-**BP**10): Br-**P**10 (9.9 g, 21.1 mmol), CH_2Cl_2 (60 mL), $FeCl_3$ (6.8 g, 42.2 mmol), CH_3NO_2 (5 mL). Purification by silica gel column chromatography (dichloromethane/petroleum ether = 1:3 v/v), recrystallized in ethyl acetate and ethanol to give a white solid 2Br-**BP**10 (6.2 g, 63%).**^{1}H NMR** ($CDCl_3$, TMS, 400 MHz) δ: 7.08 (s, 2H, ArH), 6.75 (s, 2H, ArH), 4.02-3.93 (m, 8H, OCH_2), 1.87-1.75 (m, 8H, CH_2), 1.51-1.26 (m, 56H, CH_2), 0.90-0.85 (m, 12H, CH_3).

2,2'-Dibromo-4,4',5,5'-tetradodecyloxy-1,1'-biphenyl (2Br-**BP**12): Br-**P**12 (7.0 g, 13.3 mmol), CH_2Cl_2 (40 mL), $FeCl_3$ (4.3 g, 26.6 mmol), CH_3NO_2 (5 mL). Purification by silica gel column chromatography (dichloromethane/petroleum ether = 1:3 v/v), recrystallized in ethyl acetate and ethanol to give a white solid 2Br-**BP**12 (5.4 g, 77%). **^{1}H NMR** ($CDCl_3$, TMS, 400 MHz) δ: 7.09 (s, 2H, ArH), 6.75 (s, 2H, ArH), 4.02-3.94 (m, 8H, OCH_2), 1.87-1.76 (m, 8H, CH_2), 1.51-1.25 (m, 72H, CH_2), 0.89-0.86 (m, 12H, CH_3).

2.2 Synthesis of 1,2,4-trifluoro-6,7,10,11-tetra(alkoxy)-3-(perfluorophenyl)triphenylene (F*n*) and 1,1',3,3',4,4'-hexafluoro-6,6',7,7',10,10',11,11'-octakis(alkoxy)-2,2'-bitriphenylene (G*nm*).

General procedure for the synthesis of 1,2,4-trifluoro-6,7,10,11-tetra(alkoxy)-3-(perfluorophenyl)triphenylene (**F***n*):[3] Under an argon atmosphere, 2,2'-dibromo-4,4',5,5'-tetra(alkoxy)-1,1'-biphenyl (2Br-**BP***n*, 1 equiv) was added to a 50 mL reaction tube followed by injection of THF (10 mL) for complete dissolution. The stirred solution was cooled down to −78 °C for 20 min, then *n*-BuLi (2.5 M in hexane, 4 equiv) was slowly added via a syringe. After allowing the solution to slowly warm to room temperature over 3 hours, perfluoro-1,1'-biphenyl (4 equiv) was added rapidly and stirred at 40 °C for 12 h. Then, the mixture was cooled and extracted with CH_2Cl_2. The organic phase was dried over anhydrous $MgSO_4$, filtered and spin-dried. Purification by silica gel column chromatography (dichloromethane/petroleum ether 1:4, v/v) and recrystallization from ethyl acetate and ethanol gave **F***n* as yellow solid in yields of 51–73%.

1,2,4-Trifluoro-3-(perfluorophenyl)-6,7,10,11-tetrapropoxytriphenylene (**F**3): 2Br-**BP**3 (300 mg, 0.55 mmol), THF (10 mL), *n*-BuLi (2.5 mol/L, 0.88 mL), perfluoro-1,1'-biphenyl (735 mg, 2.20 mmol). Purification by silica gel column chromatography (dichloromethane/petroleum ether = 1:4, v/v), recrystallized in ethyl acetate and ethanol to give a yellow solid **F**3 (210 mg, 56%). **^{1}H NMR** ($CDCl_3$, TMS, 400 MHz) δ: 8.51 (d, *J* = 5.7 Hz, 1H, ArH), 8.39 (d, *J* = 5.6 Hz, 1H, ArH), 7.84 (d, *J* = 1.7 Hz, 2H, ArH), 4.27-4.21 (m, 4H, OCH_2), 4.18 (t, *J* = 6.6 Hz, 2H, OCH_2), 4.13 (t, *J* = 6.5 Hz, 2H, OCH_2), 2.03-1.91 (m, 8H, CH_2), 1.18-1.08 (m, 12H, CH_3). **^{19}F NMR** ($CDCl_3$, TMS, 376 MHz) δ: -111.50 - -111.60 (m, 1F), -137.35 - -137.43 (m, 2F), -138.51 - -138.59 (m, 1F), -141.38 - -141.48 (m, 1F), -151.90 - -152.01 (m, 1F), -161.30 - -161.43 (m, 2F). **^{13}C NMR** ($CDCl_3$, TMS, 101 MHz) δ:150.47, 149.68, 148.89, 148.61, 126.08, 125.10, 122.33, 122.25, 112.23, 119.81, 115.91, 115.81, 111.45, 111.27, 111.16, 110.96, 106.47, 106.04, 70.86, 70.70, 70.52, 70.44, 22.69, 22.66, 22.58, 22.55, 10.59, 10.55. **HRMS** (ESI) Calcd for $C_{36}H_{32}F_8O_4$ $[M]^{+\bullet}$ m/z: 680.2173 (100%), 681.2206 (38.9%), 682.2240 (7.4%); found: 680.2176 (100%), 681.2209 (49%), 682.2242 (12%). **Elemental Analysis** ($C_{36}H_{32}F_8O_4$, MW 680.63): calcd (%) C 63.53, H 4.74; found (%) C 63.33, H 4.63.

6,7,10,11-Tetrabutoxy-1,2,4-trifluoro-3-(perfluorophenyl)triphenylene (**F**4): 2Br-**BP**4 (400 mg, 0.67 mmol), THF (10 mL), *n*-BuLi (2.5 mol/L, 1.07 mL), perfluoro-1,1'-biphenyl (895 mg, 2.68 mmol). Purification by silica gel column chromatography (dichloromethane/petroleum ether = 1:4, v/v), recrystallized in ethyl acetate and ethanol to give a yellow solid **F**4 (253 mg, 51%). **^{1}H NMR** ($CDCl_3$, TMS, 400 MHz) δ: 8.50 (d, *J* = 5.7 Hz, 1H, ArH), 8.39 (d, *J* = 5.5 Hz, 1H, ArH), 7.84 (s, 2H, ArH), 4.30-4.25 (m, 4H, OCH_2), 4.22 (t, *J* = 6.6 Hz, 2H, OCH_2), 4.16 (t, *J* = 6.5 Hz, 2H, OCH_2), 1.99-1.86 (m, 8H, CH_2), 1.65-1.54 (m, 8H, CH_2), 1.08-0.99 (m, 12H, CH_3). **^{19}F NMR** ($CDCl_3$, TMS, 376 MHz) δ: -111.50 - -111.60 (m, 1F), -137.35 - -137.43 (m, 2F), -138.53 - -138.62 (m, 1F), -141.38 - -141.48 (m, 1F), -151.92 - -152.03 (m, 1F), -161.31 - -161.46 (m, 2F). **^{13}C NMR** ($CDCl_3$, TMS, 101 MHz) δ: 150.51, 149.72, 148.93, 148.65, 126.09, 125.11, 122.35, 122.30, 122.24, 119.81, 115.92, 115.80, 111.47, 111.30, 111.17, 110.99, 106.46, 106.03, 69.11, 68.97, 68.81, 68.71, 31.32, 31.28, 31.25, 31.23, 19.33, 19.32, 19.31, 13.95, 13.92, 13.90. **HRMS** (ESI) Calcd for $C_{40}H_{40}F_8O_4$ $[M]^{+\bullet}$ m/z: 736.2799 (100.0%), 737.2832 (43.3%), 738.2866 (9.1%), 739.2899 (1.3%); found: 736.2796 (100%), 737.2829 (50%), 738.2862 (14%), 739.2895 (2%). **Elemental Analysis** ($C_{40}H_{40}F_8O_4$, MW 736.74): calcd (%) C 65.21, H 5.47; found (%) C 64.74, H 5.07.

1,2,4-Trifluoro-6,7,10,11-tetrakis(pentyloxy)-3-(perfluorophenyl)triphenylene (**F**5): 2Br-**BP**5 (400 mg, 0.61 mmol), THF (10 mL), *n*-BuLi (2.5 mol/L, 0.98 mL), perfluoro-1,1'-biphenyl (815 mg, 2.44 mmol). Purification by silica gel column chromatography (dichloromethane/petroleum ether = 1:4, v/v), recrystallized in ethyl acetate and ethanol to give a yellow solid **F**5 (353 mg, 73%). **^{1}H NMR** ($CDCl_3$, TMS,

400 MHz) δ: 8.50 (d, *J* = 5.1 Hz, 1H, ArH), 8.38 (d, *J* = 4.9 Hz, 1H, ArH), 7.83 (s, 2H, ArH), 4.28-4.16 (m, 8H, OCH_2), 2.01-1.88 (m, 8H, CH_2), 1.60-1.43 (m, 16H, CH_2), 1.00-0.93 (m, 12H, CH_3). **^{19}F NMR** ($CDCl_3$, TMS, 565 MHz) δ: -111.51 (s, 1F), -137.34 - -137.39 (m, 2F), -138.55 (s, 1F), -141.40 (s, 1F), -151.94 - -152.01 (m, 1F), -161.35 - -161.43 (m, 2F). **^{13}C NMR** ($CDCl_3$, TMS, 101 MHz) δ: 150.48, 149.69, 148.91, 148.66, 126.10, 125.12, 122.36, 112.31, 122.27, 119.82, 115.92, 115.83, 111.44, 111.26, 111.14, 110.95, 106.46, 106.03, 69.42, 69.27, 69.09, 68.99, 28.99, 28.95, 28.88, 28.32, 28.28, 22.55, 22.52, 22.49, 14.09, 14.08, 14.05. **HRMS** (ESI) Calcd for $C_{44}H_{48}F_8O_4$ $[M]^{+\bullet}$ m/z: 792.3425 (100.0%), 793.3458 (47.6%), 794.3492 (11.1%), 795.3526 (1.7%); found: 792.3429 (100%), 793.3462 (46%), 794.3494 (9.5%). **Elemental Analysis** ($C_{44}H_{48}F_8O_4$, MW 792.85): calcd (%) C 66.66, H 6.10; found (%) C 66.40, H 5.80.

1,2,4-Trifluoro-6,7,10,11-tetrakis(hexyloxy)-3-(perfluorophenyl)triphenylene (**F**6): 2Br-**BP**6 (400 mg, 0.56 mmol), THF (10 mL), *n*-BuLi (2.5 mol/L, 0.90 mL), perfluoro-1,1'-biphenyl (748 mg, 2.24 mmol). Purification by silica gel column chromatography (dichloromethane/petroleum ether = 1:4, v/v), recrystallized in ethyl acetate and ethanol to give a yellow solid **F**6 (264 mg, 56%). **^{1}H NMR** ($CDCl_3$, TMS, 400 MHz) δ: 8.50 (d, *J* = 5.7 Hz, 1H, ArH), 8.38 (d, *J* = 5.5 Hz, 1H, ArH), 7.83 (s, 2H, ArH), 4.29-4.24 (m, 4H, OCH_2), 4.21 (t, *J* = 6.6 Hz, 2H, OCH_2), 4.15 (t, *J* = 6.5 Hz, 2H, OCH_2), 1.99-1.88 (m, 8H, CH_2), 1.58-1.50 (m, 8H, CH_2), 1.38 (d, *J* = 10.5 Hz, 16H, CH_2), 0.95-0.89 (m, 12H, CH_3). **^{19}F NMR** ($CDCl_3$, TMS, 376 MHz) δ: -111.50 - -111.59 (m, 1F), -137.35 - -137.43 (m, 2F), -138.55 - -138.64 (m, 1F), -141.36 - -141.47 (m, 1F), -151.94 - -152.05 (m, 1F), -161.34 - -161.47 (m, 2F). **^{13}C NMR** ($CDCl_3$, TMS, 101 MHz) δ: 150.46, 149.67,148.89, 148.63, 126.08, 125.10, 122.33, 122.31, 122.24, 119.82, 115.91, 115.82, 111.37, 111.21, 111.07, 110.90, 106.44, 106.29, 106.01, 69.43, 69.28, 69.14, 69.06, 68.97, 31.66, 31.63, 29.27, 29.23, 29.15, 25.81, 25.77, 22.66, 22.62, 14.06, 14.01. **HRMS** (ESI) Calcd for $C_{48}H_{56}F_8O_4$ $[M]^{+\bullet}$ m/z: 848.4051 (100.0%), 849.4084 (51.9%), 850.4118 (13.2%), 851.4152 (2.2%); found: 848.4046 (100%), 849.4082 (52%), 850.4117 (12%), 851.4151 (1.6%). **Elemental Analysis** ($C_{48}H_{56}F_8O_4$, MW 848.96): calcd (%) C 67.91, H 6.65; found (%) C 67.71, H 6.23.

1,2,4-Trifluoro-6,7,10,11-tetrakis(octyloxy)-3-(perfluorophenyl)triphenylene (**F**8): 2Br-**BP**8 (300 mg, 0.36 mmol), THF (10 mL), *n*-BuLi (2.5 mol/L, 0.58 mL), perfluoro-1,1'-biphenyl (481 mg, 1.44 mmol). Purification by silica gel column chromatography (dichloromethane/petroleum ether = 1:5, v/v), recrystallized in ethyl acetate and ethanol to give a yellow solid **F**8 (200 mg, 58%). **^{1}H NMR** ($CDCl_3$, TMS, 400 MHz) δ: 8.49 (d, *J* = 5.1 Hz, 1H, ArH), 8.38 (d, *J* = 5.1 Hz, 1H, ArH), 7.82 (s, 2H, ArH), 4.29-4.24 (m, 4H, OCH_2), 4.21 (t, *J* = 6.5 Hz, 2H, OCH_2), 4.15 (t, *J* = 6.2 Hz, 2H, OCH_2), 1.97-1.89 (m, 8H, CH_2), 1.57-1.49 (m, 8H, CH_2), 1.36 (m, 32H, CH_2), 0.89 (d, *J* = 5.9 Hz, 12H, CH_3). **^{19}F NMR** ($CDCl_3$, TMS, 376 MHz) δ: -111.50 - -111.59 (m, 1F), -137.34 - -137.42 (m, 2F), -138.54 - -138.63 (m, 1F), -141.35 - -141.45 (m, 1F), -151.94 - -152.05 (m, 1F), -161.33 - -161.46 (m, 2F). **^{13}C NMR** ($CDCl_3$, TMS, 101 MHz) δ:150.50, 149.71,148.92, 148.67, 126.11, 125.12, 122.37, 122.31, 122.26, 119.83, 115.92, 115.82, 111.48, 111.32, 111.18, 111.01, 106.54, 106.10, 69.46, 69.32, 69.11, 69.02, 31.84, 31.81, 29.47, 29.42, 29.41, 29.33, 29.31, 29.29, 29.22, 29.20, 26.16, 26.10, 22.69, 22.67, 14.10, 14.07. **HRMS** (ESI) Calcd for $C_{56}H_{72}F_8O_4$ $[M]^{+\bullet}$ m/z: 960.5303 (100.0%), 961.5336 (60.6%), 962.5370 (18.0%), 963.5404 (3.5%); found: 960.5297 (100%), 961.5342 (64%), 962.5377 (17%), 963.5406 (3.3%). **Elemental Analysis** ($C_{56}H_{72}F_8O_4$, MW 961.17): calcd (%) C 69.98, H 7.55; found (%) C 69.82, H 7.06.

6,7,10,11-Tetrakis(decyloxy)-1,2,4-trifluoro-3-(perfluorophenyl)triphenylene (**F**10): 2Br-**BP**10 (400 mg, 0.43 mmol), THF (10 mL), *n*-BuLi ((2.5 mol/L, 0.69 mL), perfluoro-1,1'-biphenyl (575 mg, 1.72 mmol). Purification by silica gel column chromatography (dichloromethane/petroleum ether = 1:5, v/v), recrystallized in ethyl acetate and ethanol to give a yellow solid **F**10 (252 mg, 55%). **^{1}H NMR** ($CDCl_3$, TMS, 400 MHz) δ: 8.50 (d, *J* = 5.7 Hz, 1H, ArH), 8.38 (d, *J* = 5.6 Hz, 1H, ArH), 7.83 (d, *J* = 2.0 Hz, 2H, ArH), 4.29-4.24 (m, 4H, OCH_2), 4.21 (t, *J* = 6.6 Hz, 2H, OCH_2), 4.15 (t, *J* = 6.5 Hz, 2H, OCH_2), 2.00-1.86 (m, 8H, CH_2),

1.57-1.53 (m, 8H, CH_2), 1.41-1.28 (m, 48H, CH_2), 0.89-0.86 (m, 12H, CH_3). **^{19}F NMR** ($CDCl_3$, TMS, 376 MHz) δ: -111.48 - -111.58 (m, 1F), -137.34 - -137.42 (m, 2F), -138.53 - -138.62 (m, 1F), -141.35 - -141.45 (m, 1F), -151.92 - -152.03 (m, 1F), -161.31 - -161.44 (m, 2F). **^{13}C NMR** ($CDCl_3$, TMS, 101 MHz) δ: 150.46, 149.67, 148.89, 148.61, 126.06, 125.07, 122.32, 119.78, 115.87, 111.39, 111.13, 110.85, 106.51, 106.37, 106.06, 105.96, 69.45, 69.40, 69.30, 69.26, 69.09, 69.06, 69.00, 68.96, 31.95, 29.72, 29.65, 29.63, 29.41, 26.18, 26.12, 22.72, 14,13. **HRMS** (ESI) Calcd for $C_{64}H_{88}F_8O_4$ $[M]^{+\bullet}$ m/z: 1072.6555 (100.0%), 1073.6588 (69.2%), 1074.6622 (23.6%), 1075.6656 (5.3%); found: 1072.6546 (100%), 1073.6625 (74%), 1074.6704 (24%), 1075.6783 (6%). **Elemental Analysis** ($C_{64}H_{88}F_8O_4$, MW 1073.39): calcd (%) C71.61, H 8.26; found (%) C 71.50, H 7.76.

6,7,10,11-Tetrakis(dodecyloxy)-1,2,4-trifluoro-3-(perfluorophenyl)triphenylene (**F**12): 2Br-**BP**12 (400 mg, 0.38 mmol), THF (10 mL), *n*-BuLi (2.5 mol/L, 0.61 mL), perfluoro-1,1'-biphenyl (508 mg,1.52 mmol). Purification by silica gel column chromatography (dichloromethane/petroleum ether = 1:5, v/v), recrystallized in ethyl acetate and ethanol to give a yellow solid **F**12 (241 mg, 53%). **^{1}H NMR** ($CDCl_3$, TMS, 400 MHz) δ: 8.49 (d, *J* = 5.0 Hz, 1H, ArH), 8.38 (d, *J* = 5.1 Hz, 1H, ArH), 7.82 (s, 2H, ArH), 4.29-4.24 (m, 4H, OCH_2), 4.20 (t, *J* = 6.5 Hz, 2H, OCH_2), 4.15 (t, *J* = 6.3 Hz, 2H, OCH_2), 1.98-1.88 (m, 8H, CH_2), 1.54 (d, *J* = 8.7 Hz, 10H, CH_2), 1.41-1.26 (m, 62H, CH_2), 0.87 (d, *J* = 6.6 Hz, 12H, CH_3). **^{19}F NMR** ($CDCl_3$, TMS, 376 MHz) δ: -111.48 - -111.57 (m, 1F), -137.33 - -137.41 (m, 2F), -138.56 - -138.61 (m, 1F), -141.35 - -141.45 (m, 1F), -151.93 - -152.04 (m, 1F), -161.32 - -161.45 (m, 2F). **^{13}C NMR** ($CDCl_3$, TMS, 400 MHz) δ: 150.48, 149.69, 148.91, 148.63, 126.08, 125.09, 122.35, 122.30, 122.24, 120.10, 119.79, 119.73, 115.93, 115.81, 111.39, 111.22, 111.10, 110.90, 106.45, 106.02, 69.42, 69.27, 69.07, 68.97, 31.95, 29.75, 29.69, 29.54, 29.49, 29.41, 29.35, 29.30, 29.24, 29.20, 26.17, 26.11, 22.71, 14.12. **HRMS** (ESI) Calcd for $C_{72}H_{104}F_8O_4$ $[M]^{+\bullet}$ m/z: 1184.7807 (100.0%), 1185.7840 (77.9%), 1186.7874 (29.9%), 1187.7908 (7.5%), 1188.7941 (1.4%), 1185.7870 (1.2%); found: 1184.7797 (100%). **Elemental Analysis** ($C_{72}H_{104}F_8O_4$, MW 1185.61): calcd (%) C 72.94, H 8.84; found (%) C 73.16, H 8.44.

General procedure for the synthesis of 1,1',3,3',4,4'-hexafluoro-6,6',7,7',10,10',11,11'-octakis(alkoxy)-2,2'-bitriphenylene (**G***nm*): Under an argon atmosphere, 2Br-**BP***n* (2 equiv) was added to a 50 mL reaction tube followed by injection of THF (10 mL) for complete dissolution. The stirred solution was cooled to −78 °C for 20 min, then *n*-BuLi (2.5 mol/L, 8 equiv) was slowly added via syringe. After allowing the solution to slowly warm to room temperature over 3 hours, **F***n* (1 equiv) was added rapidly and stirred at 40 °C for 12 h. Then, the mixture was cooled and extracted with CH_2Cl_2. The organic phase was dried over anhydrous $MgSO_4$, filtered and spin-dried. Purification by silica gel column chromatography (dichloromethane/petroleum ether = 1:3, v/v) and recrystallization from ethyl acetate and ethanol gave **G***nm* as yellow solid in yields of 42–45%.

1,1',3,3',4,4'-Hexafluoro-6,6',7,7',10,10',11,11'-octakis(pentyloxy)-2,2'-bitriphenylene (**G**55): 2Br-**BP**5 (249 mg, 0.38 mmol), THF (10 mL), *n*-BuLi (2.5 mol/L, 0.61 mL), **F**5 (150 mg, 0.19 mmol). Purification by silica gel column chromatography (dichloromethane/petroleum ether = 1:3, v/v), recrystallized in ethyl acetate and ethanol to give a yellow solid **G**55 (107 mg, 45%). **^{1}H NMR** ($CDCl_3$, TMS, 400 MHz) δ: 8.53 (d, *J* = 5.9 Hz, 2H, ArH), 8.45 (d, *J* = 4.8 Hz, 2H, ArH), 7.84 (s, 4H, ArH), 4.31-4.21 (m, 12H, OCH_2), 4.14 (t, *J* = 6.5 Hz, 4H, OCH_2), 2.01-1.86 (m, 16H, CH_2), 1.60-1.53 (m, 14H, CH_2), 1.49-1.35 (m, 18H, CH_2), 1.01-0.88 (m, 24H, CH_3). **^{19}F NMR** ($CDCl_3$, TMS, 565 MHz) δ: -111.10 (s, 2F), -137.87 - -137.91 (m, 2F), -142.05 (s, 2F). **^{13}C NMR** ($CDCl_3$, TMS, 101 MHz) δ: 150.23, 149.46, 148.86, 148.58, 125.92, 124.99, 121.57, 120.17, 120.04, 115.77, 115.72, 111.49, 111.32, 111.19, 111.01, 106.52, 106.12, 69.44, 69.30, 69.08, 68.92, 29.02, 29.00, 28.92, 28.89, 28.35, 28.32, 28.31, 28.26, 22.57, 22.56, 22.55, 22.47, 14.12, 14.11, 14.01. **HRMS** (ESI) Calcd for $C_{76}H_{96}F_6O_8$ $[M]^{+\bullet}$ m/z: 1250.7009 (100.0%), 1251.7043 (82.2%), 1252.7076 (33.3%), 1253.7110 (8.9%), 1254.7144 (1.8%); found: 1250.6980 (100%), 1251.7022 (90%), 1252.7061

(41%), 1253.7098 (12%), 1254.7135 (1.5%). **Elemental Analysis** ($C_{76}H_{96}F_6O_8$, MW 1251.59): calcd (%) C 72.93, H 7.73; found (%) C 72.47, H 7.26.

1,1',3,3',4,4'-Hexafluoro-6,6',7,7',10,10',11,11'-octakis(hexyloxy)-2,2'-bitriphenylene (**G**66): 2Br-**BP**6 (257 mg, 0.36 mmol), THF (10 mL), *n*-BuLi (2.5 mol/L, 0.58 mL), **F**6 (150 mg, 0.18 mmol). Purification by silica gel column chromatography (dichloromethane/petroleum ether = 1:3, v/v), recrystallized in ethyl acetate and ethanol to give a yellow solid **G**66 (104 mg, 42%). **^{1}H NMR** ($CDCl_3$, TMS, 600 MHz) δ: 8.54 (d, *J* = 5.9 Hz, 2H, ArH), 8.46 (d, *J* = 5.1 Hz, 2H, ArH), 7.85 (s, 4H, ArH), 4.30-4.22 (m, 12H, OCH_2), 4.14 (t, *J* = 6.5 Hz, 4H, OCH_2), 2.00-1.93 (m, 12H, CH_2), 1.89-1.84 (m, 4H, CH_2), 1.61-1.57 (m, 12H, CH_2), 1.52-1.39 (m, 28H, CH_2), 1.35-1.29 (m, 8H, CH_2), 0.95-0.84 (m, 24H, CH_3). **^{19}F NMR** ($CDCl_3$, TMS, 565 MHz) δ: -111.09(s, 2F), -137.89 - -137.91(m, 2F), -142.04(s, 2F). **^{13}C NMR** ($CDCl_3$, TMS, 101 MHz) δ: 150.20, 149.44, 148.85, 148.57, 125.90, 124.97, 121.57, 120.19, 120.03, 115.78,115.68, 111.45, 111.29, 111.15, 110.97, 106.53, 106.11, 69.45, 69.31, 69.06, 68.91, 31.68, 31.66, 31.59, 29.32, 29.30, 29.19, 29.18, 25.84, 25.82, 25.80, 25.74, 22.66, 22.57, 14.06, 13.97. **HRMS** (ESI) Calcd for $C_{84}H_{112}F_6O_8$ $[M]^{+\bullet}$ m/z: 1362.8261 (100.0%), 1363.8295 (90.9%), 1364.8328 (40.8%), 1365.8362 (12.1%); found: 1362.8263 (100%), 1363.8298 (91%), 1364.8334 (46%), 1365.8366 (14%). **Elemental Analysis** ($C_{84}H_{112}F_6O_8$, MW 1363.80): calcd (%) C 73.98, H 8.28; found (%) C 74.19, H 7.94.

The unsymmetrical fluorinated triphenylene **G**48, the isomer of **G**66, was synthesized and purified according to the general procedure, but reacting 2Li-**BP**8 (from 2Br-**BP**8) and **F**4, and obtained in 50% yields. **^{1}H NMR** (600 MHz, $CDCl_3$) *δ* 8.53 (t, *J* = 5.6 Hz, 2H, ArH), 8.46 (t, *J* = 4.9 Hz, 2H, ArH), 7.86 – 7.81 (m, 4H, ArH), 4.32 – 4.21 (m, 12H, OCH_2), 4.14 (q, *J* = 6.9 Hz, 4H, OCH_2), 2.00 – 1.92 (m, 12H, CH_2), 1.86 (dq, *J* = 12.5, 6.4 Hz, 4H, CH_2), 1.67 – 1.54 (m, 14H, CH_2), 1.54 – 1.46 (m, 4H, CH_2), 1.45 – 1.39 (m, 6H, CH_2), 1.38 – 1.29 (m, 20H, CH_2), 1.26 – 1.21 (m, 4H, CH_2), 1.06 (m, 9H, CH_3), 0.97 (m, 3H, CH_3), 0.90 (m, 9H, CH_3), 0.82 (m, 3H, CH_3). **^{19}F NMR** (565 MHz, $CDCl_3$) *δ* -111.11, -137.89, -142.02, -142.05. **^{13}C NMR** (151 MHz, $CDCl_3$) *δ* 150.28, 149.52, 148.91, 148.63, 125.97, 125.05, 121.63, 120.26, 120.10, 115.80, 111.52, 111.33, 111.19, 106.62, 106.20, 69.52, 69.36, 69.18, 69.12, 69.03, 68.99, 68.83, 68.69, 31.86, 31.77, 31.36, 31.33, 31.27, 31.25, 29.49, 29.45, 29.39, 29.38, 29.35, 29.33, 29.26, 29.23, 26.19, 26.17, 26.13, 26.08, 22.71, 22.64, 19.36, 19.34, 19.29, 14.13, 14.05, 13.99, 13.98, 13.96, 13.90. **HRMS** (ESI) Calcd for $C_{84}H_{112}F_6O_8$ $[M]^{+\bullet}$ m/z: 1362.8261 (100%), 1363.8295 (91%), 1364.8328 (41%), 1365.8362 (12%), 1366.8396 (2.6%) found: 1362.8231 (100%), 1363.8255 (95%), 1364.8281 (45%), 1365.8327 (14%), 1366.8402(2%).

2.3 Synthesis of the 1,2,4-trifluoro-3-(perfluorophenyl)triphenylene (F)

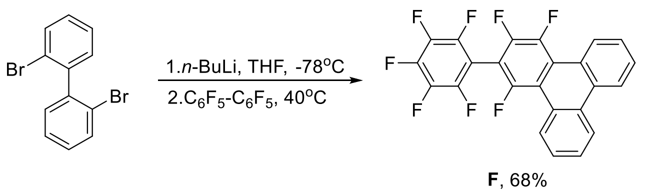

Scheme S2. Synthesis of the 1,2,4-trifluoro-3-(perfluorophenyl)triphenylene (**F**)

1,2,4-Trifluoro-3-(perfluorophenyl)triphenylene (**F**): Under an argon atmosphere, 2,2'-dibromo-1,1'-biphenyl (200 mg, 0.64 mmol) was added to a 50 mL reaction tube followed by injection of THF (10 mL) for complete dissolution. The stirred solution was cooled to −78 °C for 20 min, then *n*-BuLi (2.5 mol/L, 1.02 mL) was slowly added via syringe. After allowing the solution to slowly warm to room temperature over 3 hours, perfluoro-1,1'-biphenyl (855 mg, 2.56 mmol) was added rapidly and stirred at 40 °C for 12 h. Then, the mixture was cooled and extracted with CH_2Cl_2. The organic phase was dried over anhydrous

$MgSO_4$, filtered and spin-dried. Recrystallization from ethanol gave **F** as yellow solid (195 mg, 68%). **^{1}H NMR** ($CDCl_3$, TMS, 400 MHz) δ: 9.04-9.01 (m, 1H, ArH), 8.93-8.90 (m, 1H, ArH), 8.66-8.63 (m, 2H, ArH), 7.78-7.63 (m, 4H, ArH). **^{19}F NMR** ($CDCl_3$, TMS, 565 MHz) δ: -109.84 (s, 1F), -135.76 - -135.79 (m, 1F), -137.33 - -137.37 (m, 2F), -139.45 - -139.50 (m, 1F), -151.35 - -151.43 (m, 1F), -161.09 - -161.17 (m, 2F). **^{13}C NMR** ($CDCl_3$, TMS, 101 MHz) δ: 131.08, 130.35, 129.22, 129.19, 128.40, 128.36, 128.34, 128.11, 127.98, 127.96, 127.88, 127.85, 127.66, 126.05, 125.99, 123.22. **HRMS** (ESI) Calcd for $C_{24}H_8F_8$ $[M]^{+\bullet}$ m/z: 448.0498 (100.0%), 449.0532 (26.0%), 450.0565 (3.2%); found: 448.0496 (100%), 449.0527 (25%), 450.0560 (3.0%).

2.4 Synthesis of 2,3,6,7-tetrakis(hexyloxy)-10-phenyltriphenylene (BTP6)

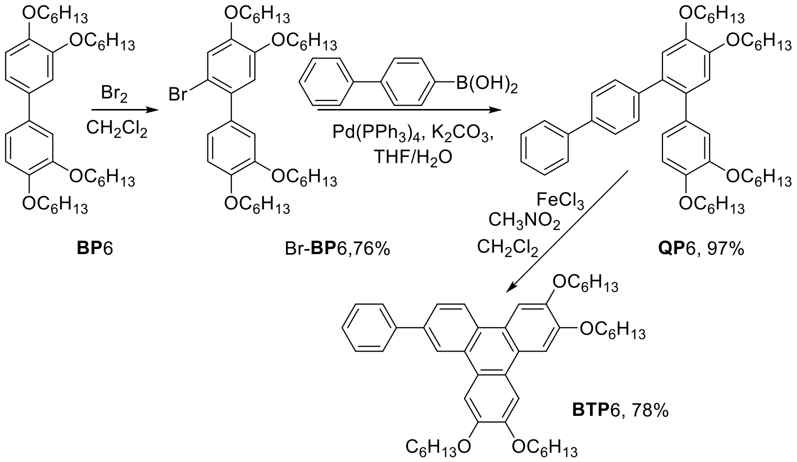

Scheme S3. Synthesis of 3,4,4',5'-tetrakis(hexyloxy)-1,1':2',1'':4'',1'''-quaterphenyl (**QP**6), and 2,3,6,7-tetrakis(hexyloxy)-10-phenyltriphenylene (**BTP**6).

2-Bromo-3',4,4',5-tetrakis(hexyloxy)-1,1'-biphenyl (Br-**BP**6):[3] To a 250 mL round-bottomed flask, containing **BP**6 (2.5 g, 4.6 mmol) in 100 mL dichloromethane, was added slowly by a constant-pressure dropping funnel, bromine (0.77 g, 4.8 mmol) in dichloromethane (50 mL), and the mixture kept at room temperature. After completion of the reaction (TLC), aqueous sodium hydrogen sulfite solution was added, and the mixture extracted with dichloromethane. The organic phase was dried over anhydrous $MgSO_4$, filtered, and the solvent removed by rotary evaporation. Purification by silica gel column chromatography (dichloromethane/petroleum ether = 1:2, v/v) and recrystallization from ethanol and ethyl acetate gave Br-**BP**6 as white solid (2.2 g, 76%). **^{1}H NMR** ($CDCl_3$, TMS, 400 MHz) δ: 7.11 (s, 1H, ArH), 6.95 (s, 1H, ArH), 6.92-6.87 (m, 2H, ArH), 6.84 (s, 1H, ArH), 4.05-3.95 (m, 8H, OCH_2), 1.87-1.76 (m, 8H, CH_2), 1.49-1.42 (m, 8H, CH_2), 1.39-1.28 (m, 16H, CH_2), 0.93-0.88 (m, 12H, CH_3).

3,4,4',5'-Tetrakis(hexyloxy)-1,1':2',1'':4'',1'''-quaterphenyl (**QP**6): In a 50 mL reaction tube, Br-**BP**6 (400 mg, 0.63 mmol), [1,1'-biphenyl]-4-ylboronic acid (188 mg, 0.95 mmol), K_2CO_3 (1738 mg, 12.60 mmol) and $Pd(PPh_3)_4$ (69 mg, 0.06 mmol) were mixed, and H_2O (3 mL) and THF (10 mL) were added. Under argon gas, the reaction was stirred at 70 °C for 48 h, cooled to room temperature, and extracted with dichloromethane. The organic phase was dried over anhydrous $MgSO_4$, filtered and spin-dried. Purified by silica gel column chromatography (dichloromethane/petroleum ether = 1:4, v/v), and recrystallization from ethyl acetate and ethanol gave **QP**6 as white solid (431 mg, 97%). **^{1}H NMR** ($CDCl_3$, TMS, 400 MHz) δ: 7.57 (d, *J* = 7.6 Hz, 2H, ArH), 7.47-7.40 (m, 4H, ArH), 7.32 (t, *J* = 7.3 Hz, 1H, ArH), 7.21 (d, *J* = 8.0 Hz, 2H, ArH), 6.98 (s, 2H, ArH), 6.79 (s, 2H, ArH), 6.55 (s, 1H, ArH), 4.09-4.06 (m, 4H, OCH_2), 3.96 (t, *J* = 6.7 Hz, 2H, OCH_2), 3.61 (t, *J* = 6.7 Hz, 2H, OCH_2), 1.89-1.76 (m, 6H, CH_2), 1.62-1.57 (m, 2H, CH_2), 1.50-1.43 (m, 6H, CH_2), 1.36-1.19 (m, 18H, CH_2), 0.93-0.81 (m, 12H, CH_3). **^{13}C NMR** ($CDCl_3$, TMS,

101 MHz) δ: 148.45, 148.20, 148.17, 147.65, 140.89, 140.75, 138.78, 134.13, 133.01, 132.47, 130.30, 128.70, 127.13, 126.88, 126.54, 121.82, 116.29, 116.09, 115.98, 113.26, 69.50, 69.47, 69.17, 68.95, 31.63, 31.61, 31.48, 29.33, 29.29, 29.00,25.74, 25.71, 25.62, 22.64, 22.61, 22.58, 14.05, 14.03, 13.97.

2,3,6,7-Tetrakis(hexyloxy)-10-phenyltriphenylene (**BTP**6): A solution of **QP**6 in CH_2Cl_2 (20 mL) was placed in a 100 mL round-bottomed flask and a solution of $FeCl_3$ (136 mg, 0.84 mmol) in CH_3NO_2 (2 mL) was added. The resulting solution was stirred at room temperature until completion of the reaction. The reaction was quenched with methanol and extracted with dichloromethane. The organic phase was dried over anhydrous $MgSO_4$, filtered and spin-dried. Purification by silica gel column chromatography (dichloromethane/petroleum ether = 1:2, v/v) and recrystallization from ethyl acetate and ethanol gave **J** as white solid (154 mg, 78%). **^{1}H NMR** ($CDCl_3$, TMS, 400 MHz) δ: 8.63 (s, 1H), 8.54 (d, *J* = 8.54 Hz, 1H, ArH), 8.08 (s, 1H, ArH), 8.03 (s, 1H, ArH), 7.85-7.79 (m, 5H, ArH), 7.54 (t, *J* = 7.4 Hz, 2H, ArH), 7.43 (t, *J* = 7.1 Hz, 1H, ArH), 4.26 (t, *J* = 5.9 Hz, 8H, OCH_2), 1.98-1.94 (m, 8H, CH_2), 1.59 (s, 6H, CH_2), 1.41 (d, *J* = 2.7 Hz, 14H, CH_2), 1.26 (s, 4H, CH_2), 0.96-0.88 (m, 12H, CH_3). **^{13}C NMR** ($CDCl_3$, TMS, 101 MHz) δ: 149.30, 141.60, 138.56, 128.87, 127.50, 127.29,125.20, 124.47, 123.47, 121.39, 107.04, 69.68, 69.62, 69.51, 69.32, 31.70, 29.42, 29.36, 25.86, 22.68, 14.07. **HRMS** (ESI) Calcd for $C_{48}H_{64}O_4$ $[M]^{+\bullet}$ m/z: 704.4805 (100.0%), 705.4838 (51.9%), 706.4872 (13.2%), 707.4905 (2.2%); found: 704.4810 (100%), 705.4884 (50%), 706.4870 (12%), 707.4901 (1.7%).

3. ^{1}H, ^{19}F and ^{13}C NMR

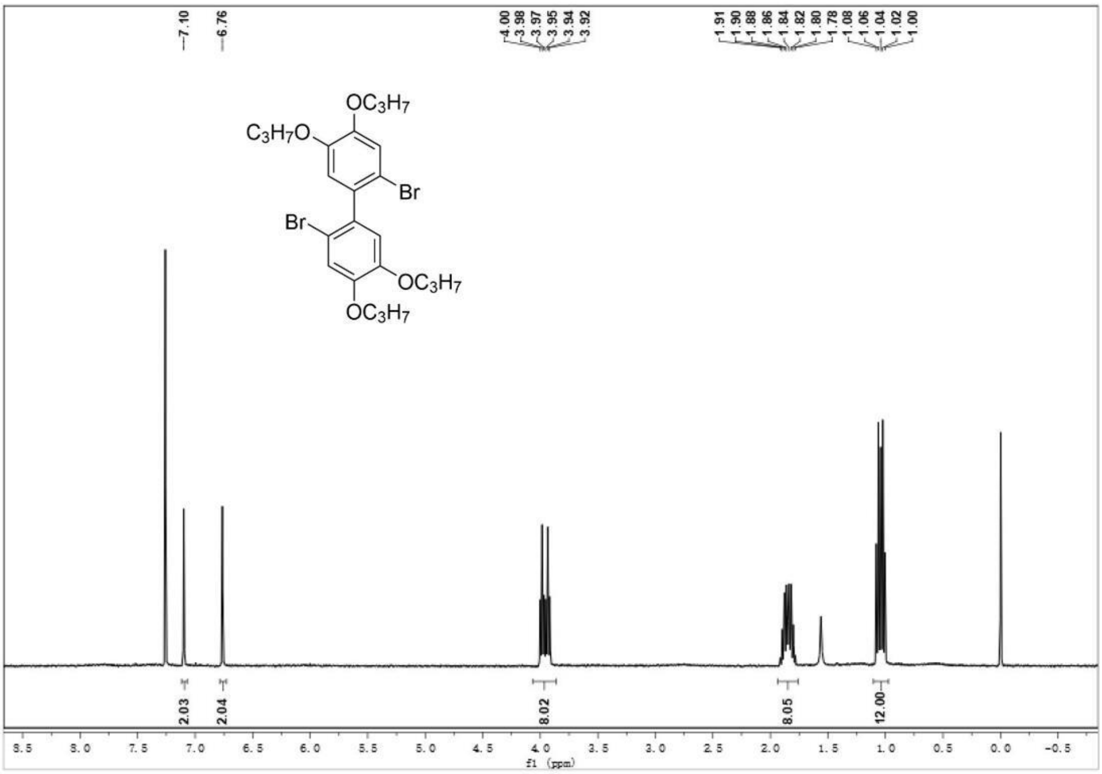

Figure S1. ^{1}H NMR ($CDCl_3$, 400 MHz) spectrum of 2Br-**BP**3.

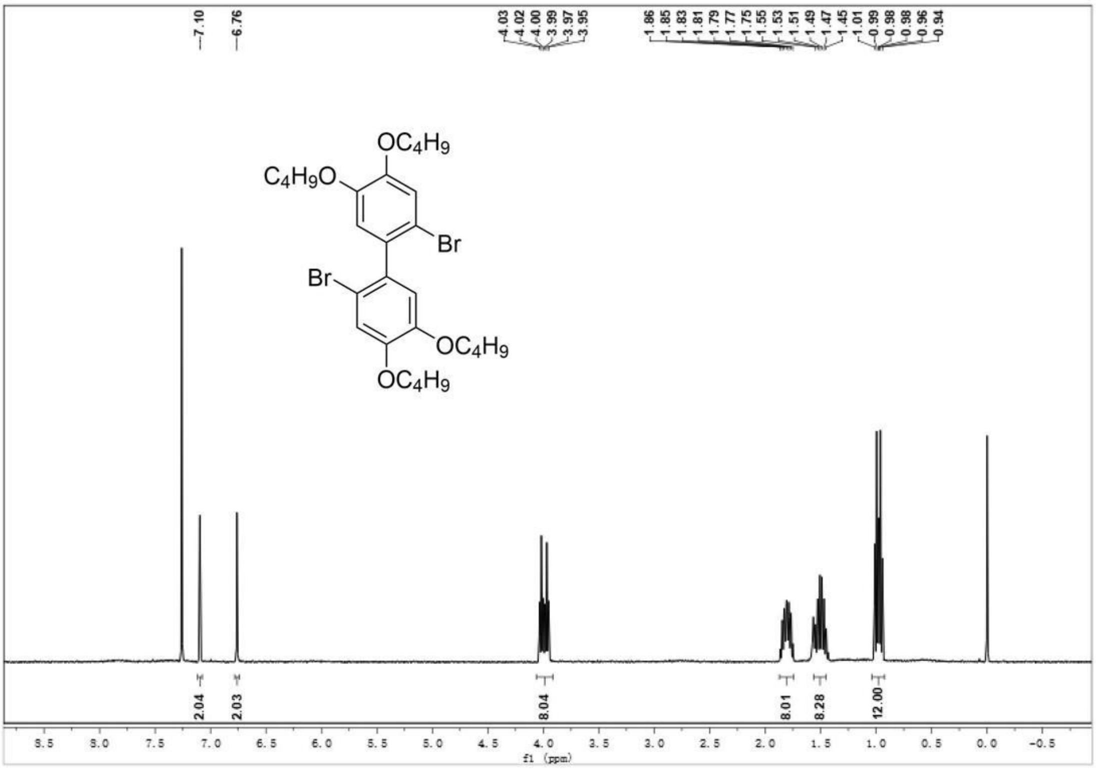

Figure S2. ^{1}H NMR ($CDCl_3$, 400 MHz) spectrum of 2Br-**BP**4.

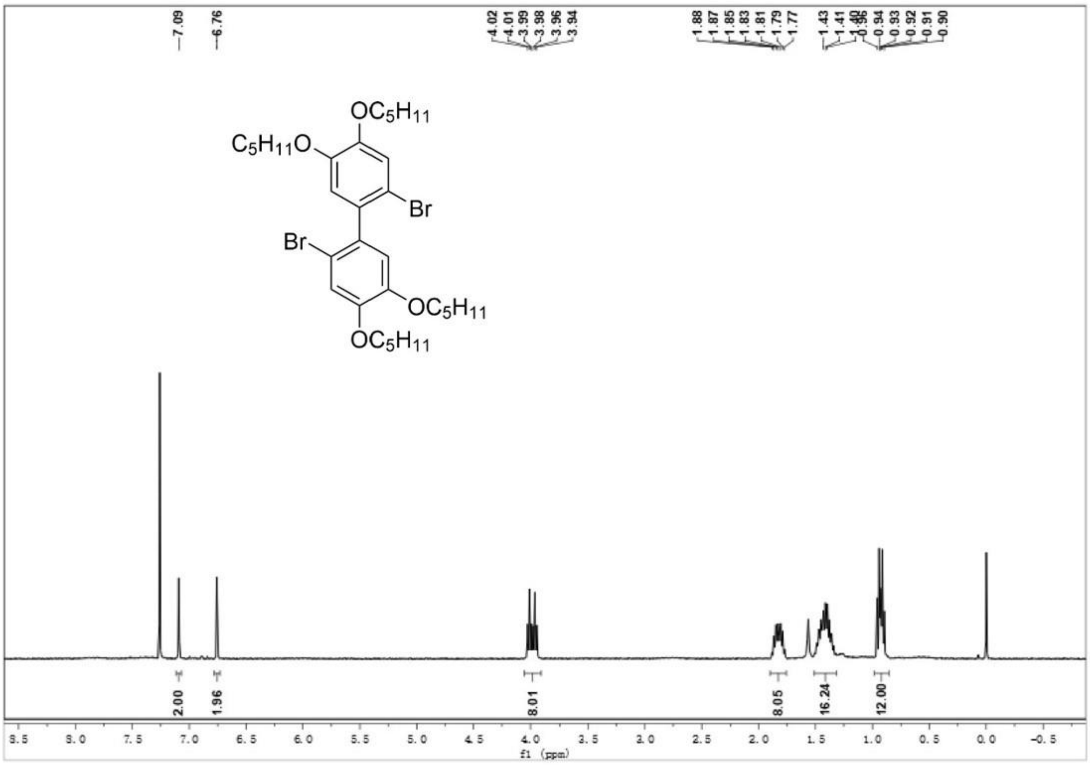

Figure S3. ^{1}H NMR ($CDCl_3$, 400 MHz) spectrum of 2Br-**BP**5.

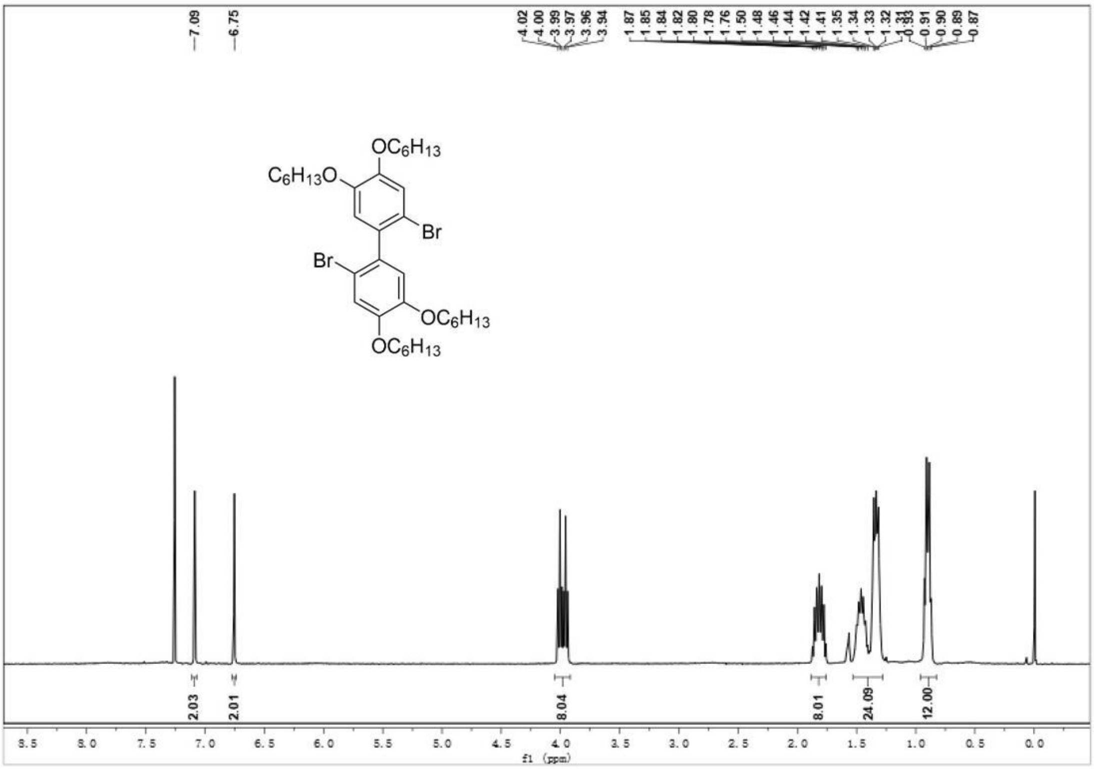

Figure S4. ^{1}H NMR ($CDCl_3$, 400 MHz) spectrum of 2Br-**BP**6.

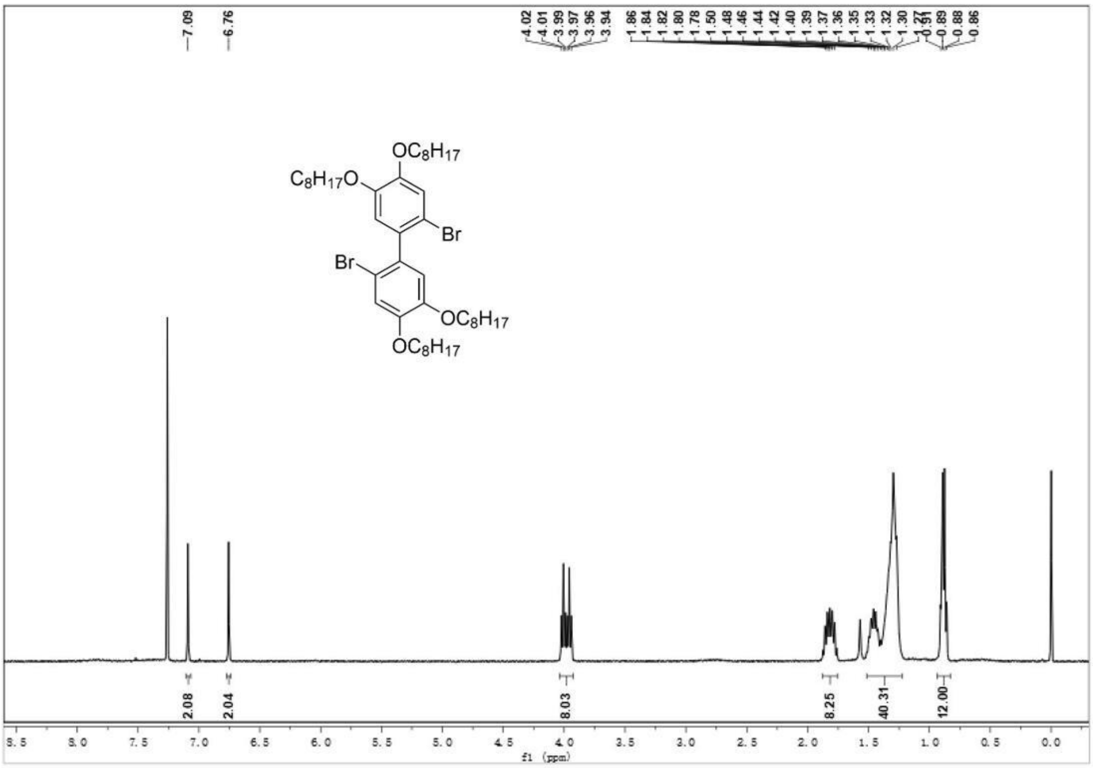

Figure S5. ^{1}H NMR ($CDCl_3$, 400 MHz) spectrum of 2Br-**BP**8.

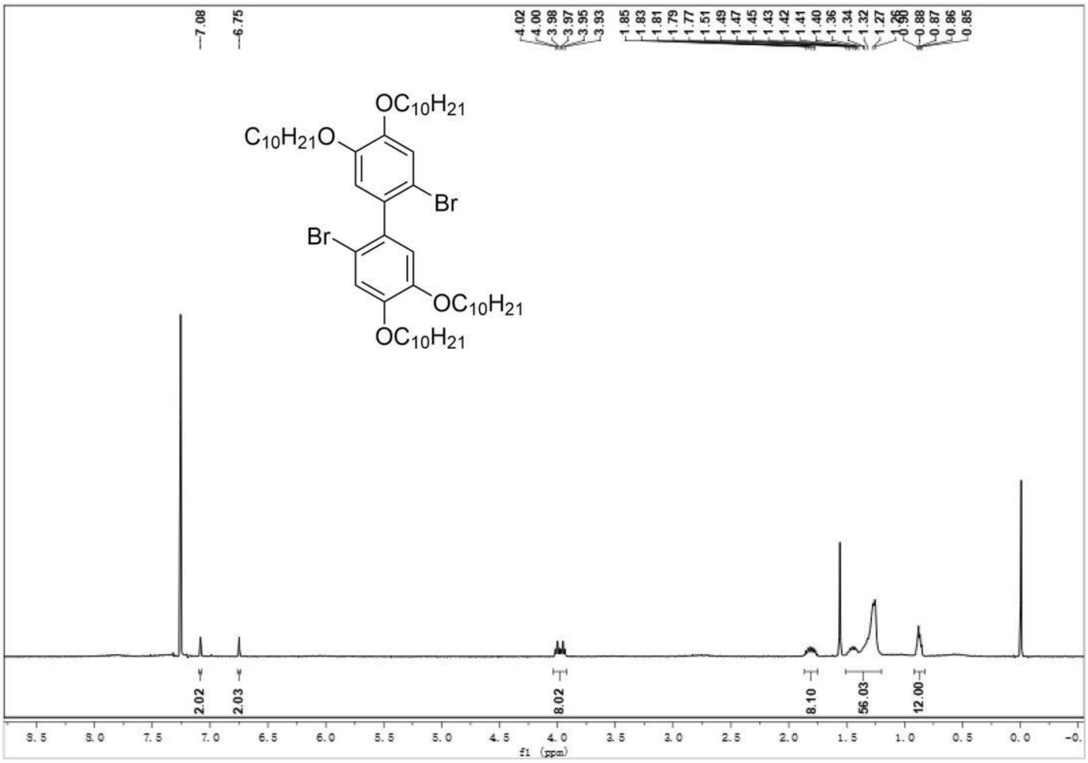

Figure S6. ^{1}H NMR ($CDCl_3$, 400 MHz) spectrum of 2Br-**BP**10.

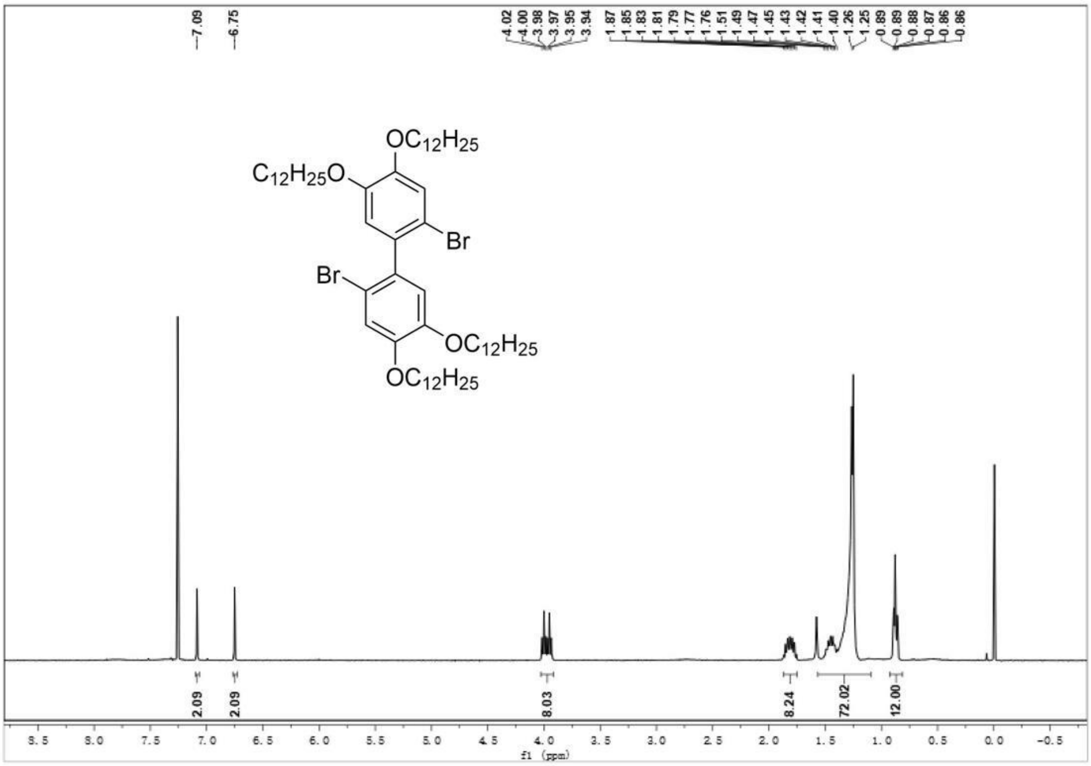

Figure S7. ^{1}H NMR ($CDCl_3$, 400 MHz) spectrum of 2Br-**BP**12.

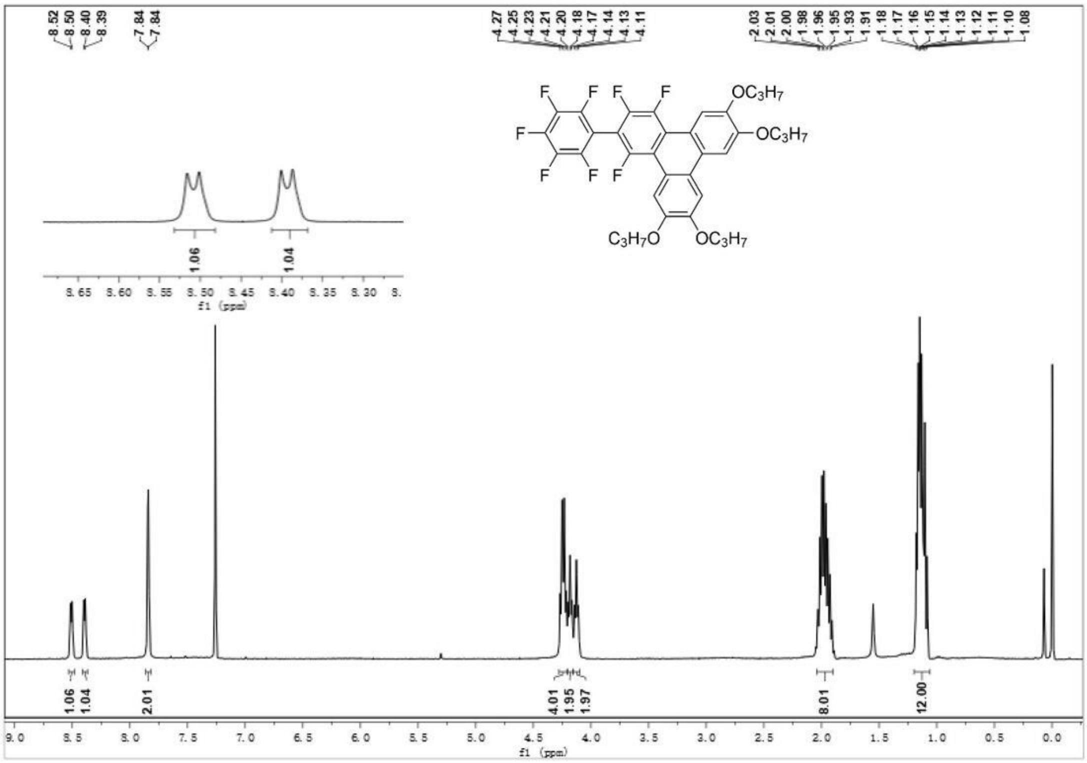

8.52
8.50
8.40
8.39
7.84
7.84
4.27
4.25
4.23
4.21
4.20
4.18
4.17
4.14
4.13
4.11
2.03
2.01
2.00
1.98
1.96
1.95
1.93
1.91
1.18
1.17
1.16
1.15
1.14
1.13
1.12
1.11
1.10
1.08
OC3H7
OC3H7
C3H7O
OC3H7
1.06
1.04
f1 (ppm)
1.06
1.04
2.01
4.01
1.95
1.97
8.01
12.00
f1 (ppm)

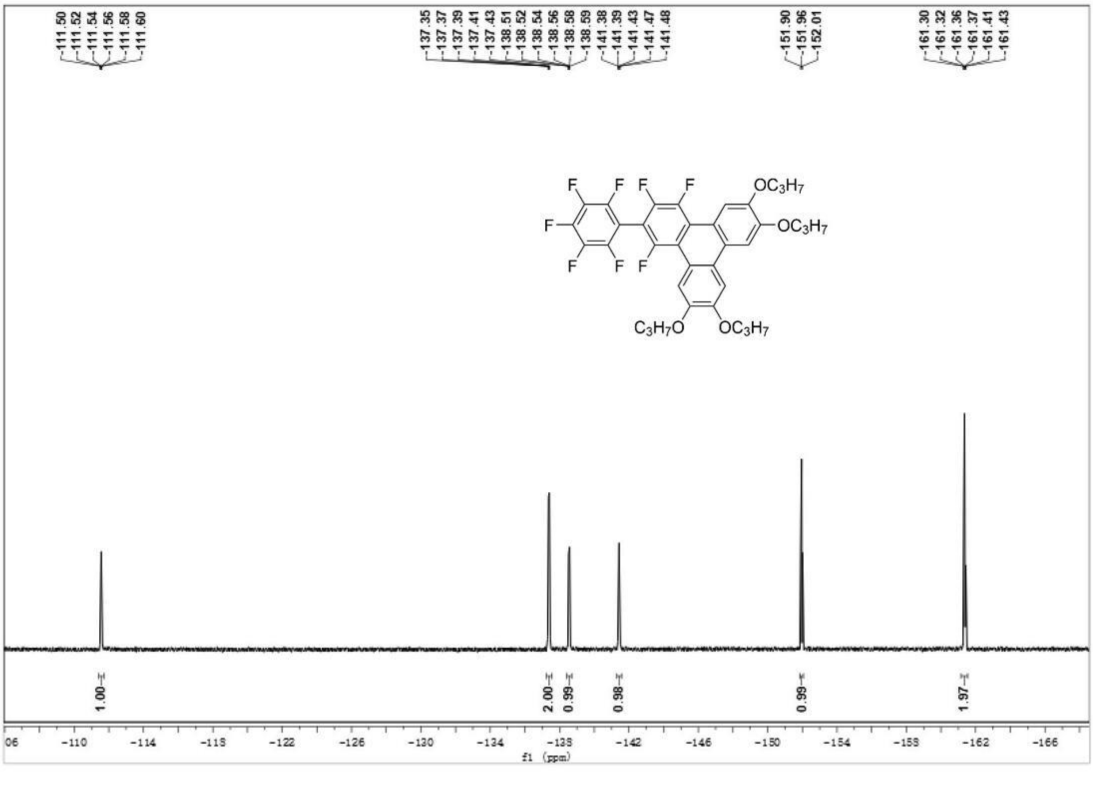

-111.50
-111.52
-111.54
-111.56
-111.58
-111.60
-137.35
-137.37
-137.39
-137.41
-137.43
-138.51
-138.52
-138.54
-138.56
-138.58
-138.59
-141.38
-141.39
-141.43
-141.47
-141.48
-151.90
-151.96
-152.01
-161.30
-161.32
-161.36
-161.37
-161.41
-161.43
OC3H7
OC3H7
C3H7O
OC3H7
1.00
2.00
0.99
0.98
0.99
1.97
f1 (ppm)

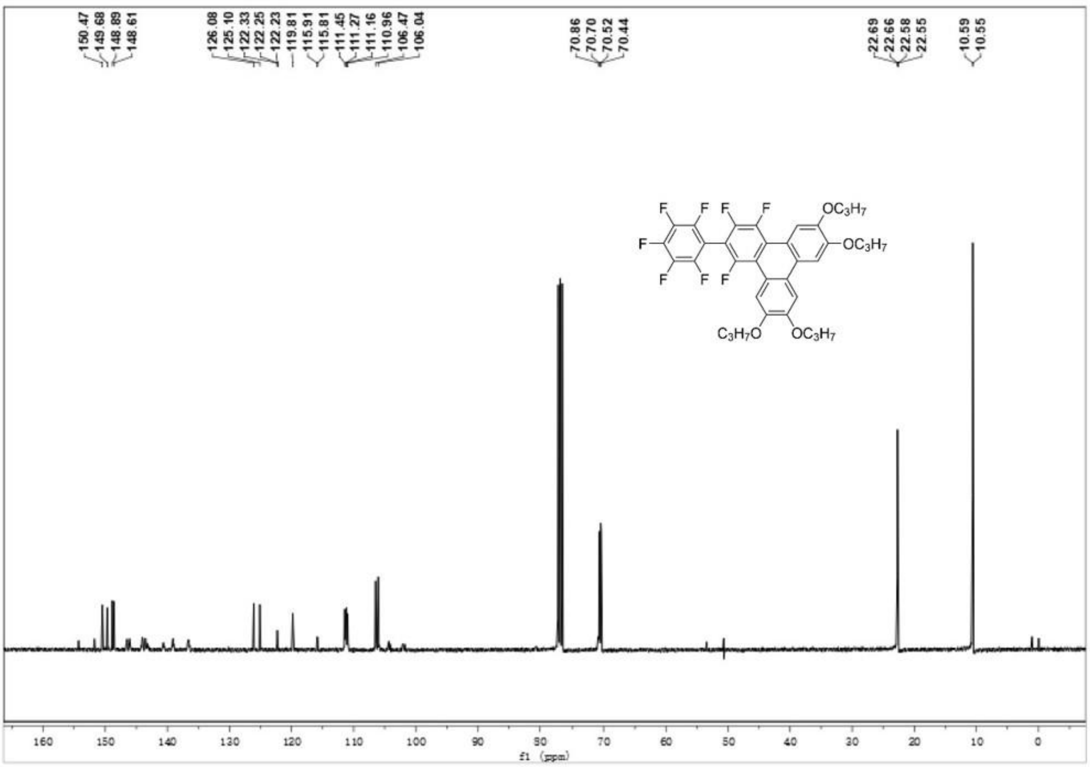

Figure S8. ^{1}H NMR ($CDCl_3$, 400 MHz), ^{19}F NMR ($CDCl_3$, 376 MHz) and ^{13}C NMR ($CDCl_3$, 101 MHz) spectra of **F**3.

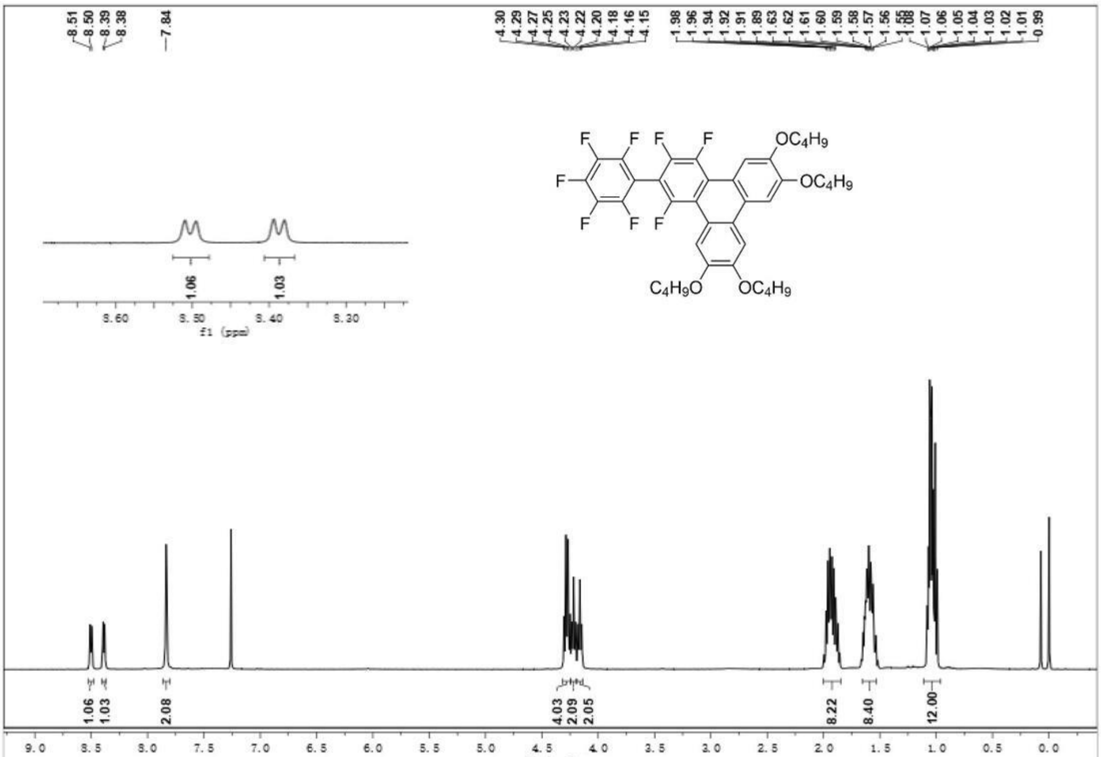

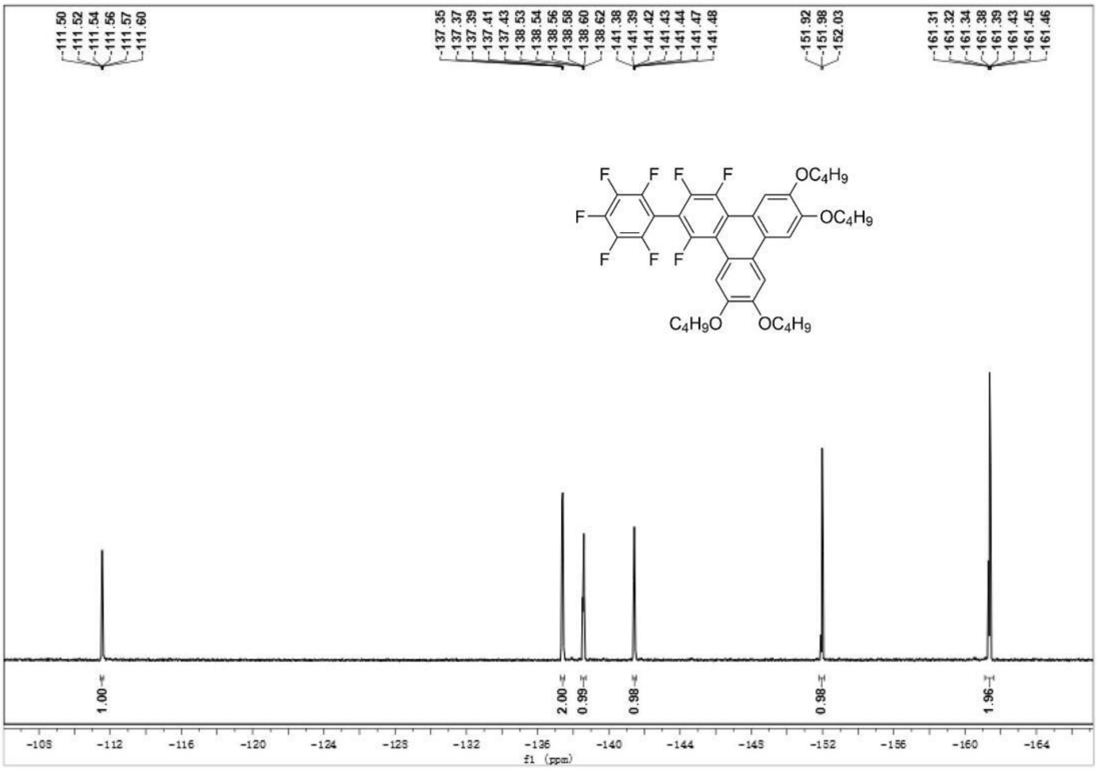

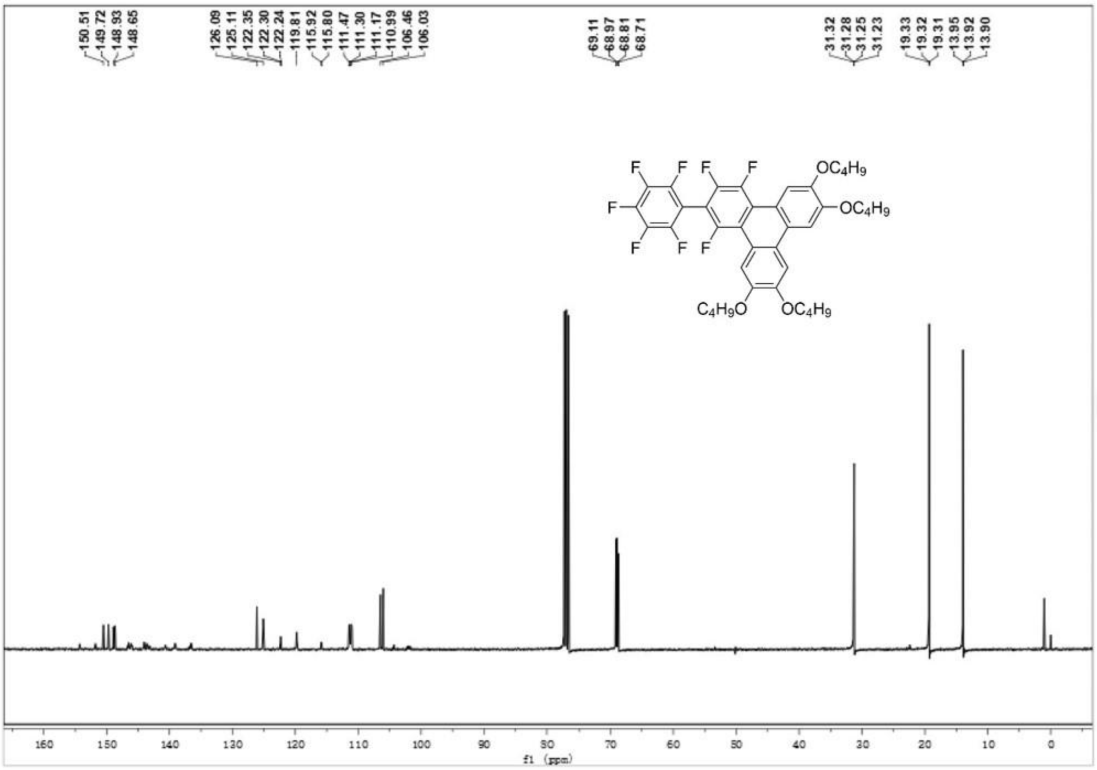

Figure S9. ^{1}H NMR ($CDCl_3$, 400 MHz), ^{19}F NMR ($CDCl_3$, 376 MHz) and ^{13}C NMR ($CDCl_3$, 101 MHz) spectra of **F**4.

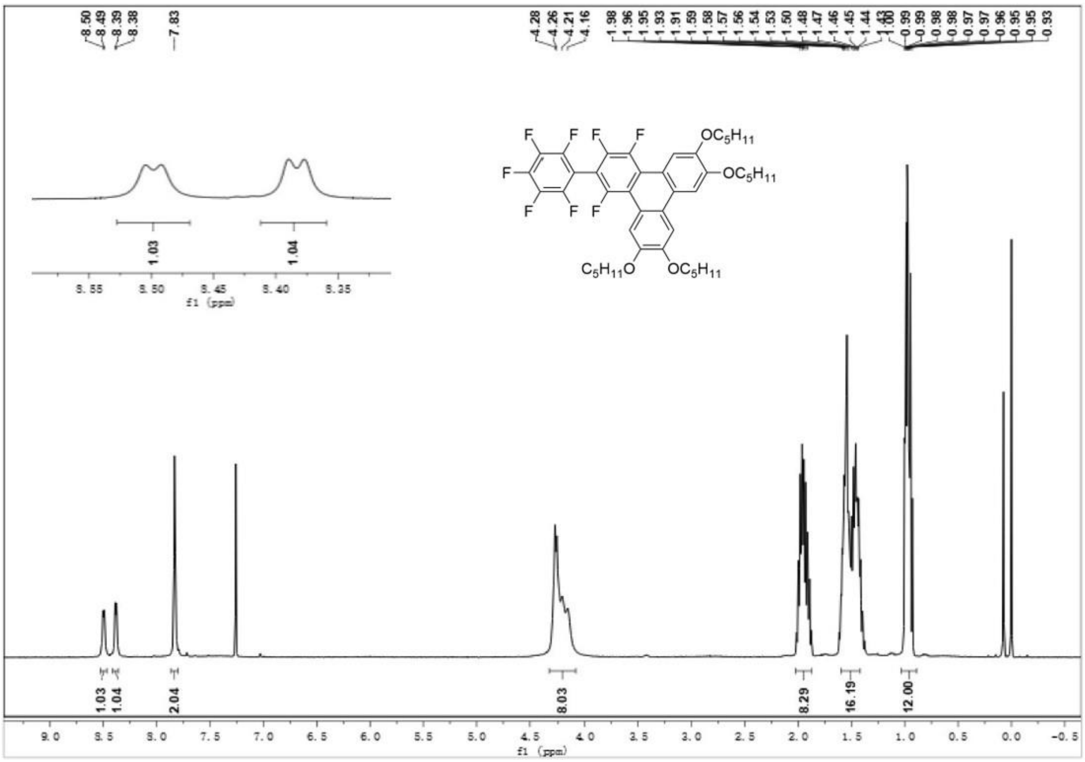

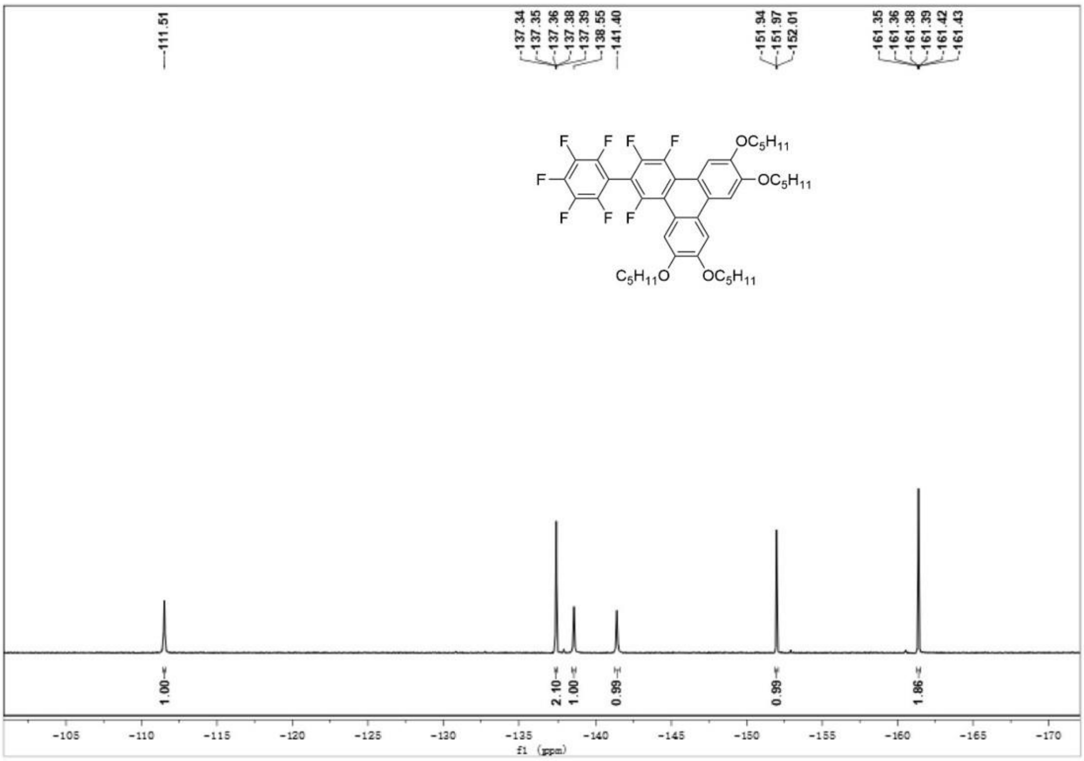

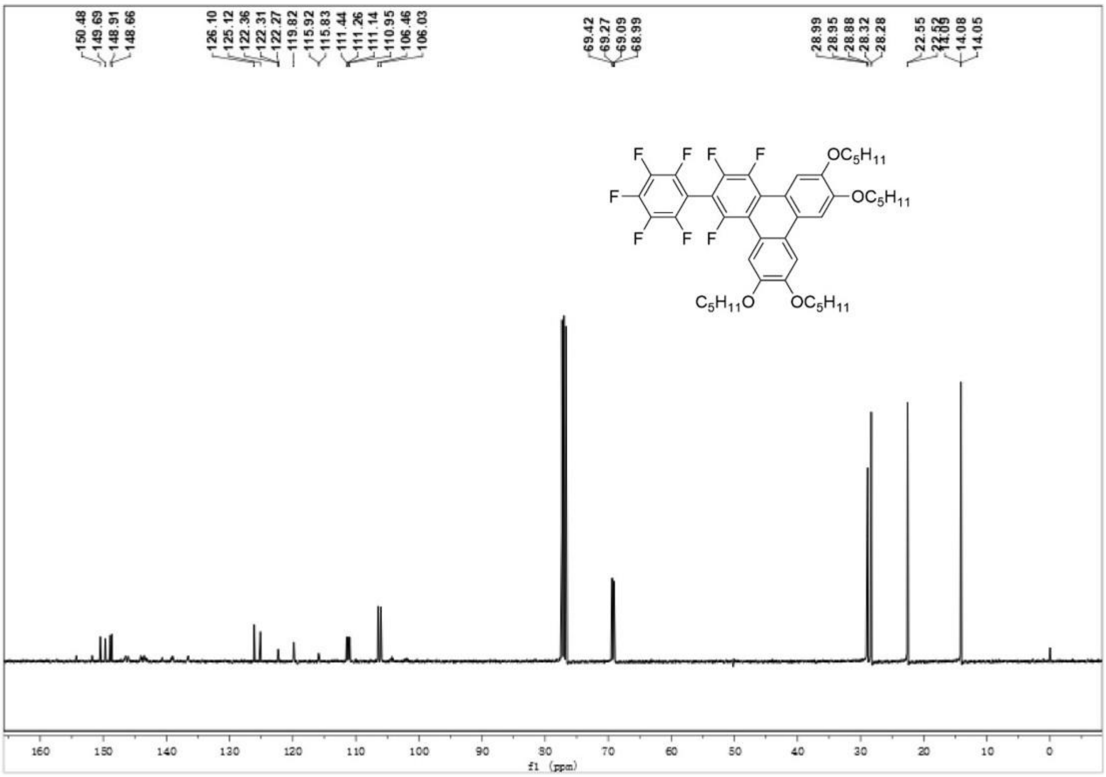

Figure S10. ^{1}H NMR ($CDCl_3$, 400 MHz), ^{19}F NMR ($CDCl_3$, 376 MHz) and ^{13}C NMR ($CDCl_3$, 101 MHz) spectra of **F**5.

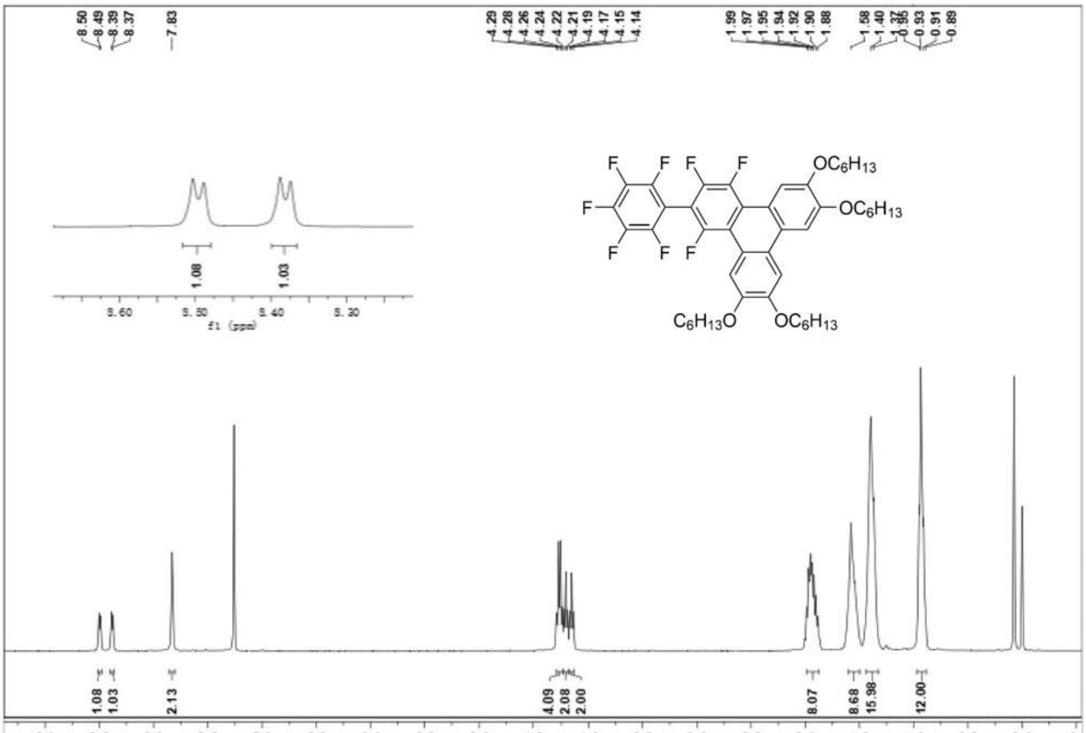

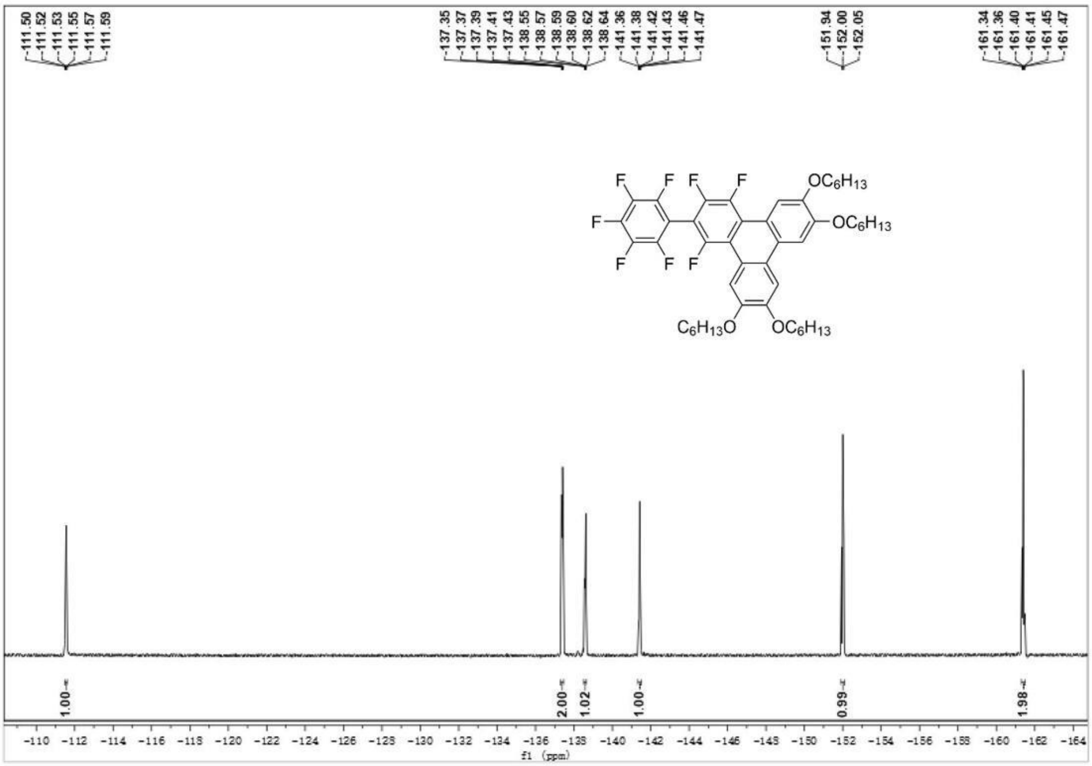

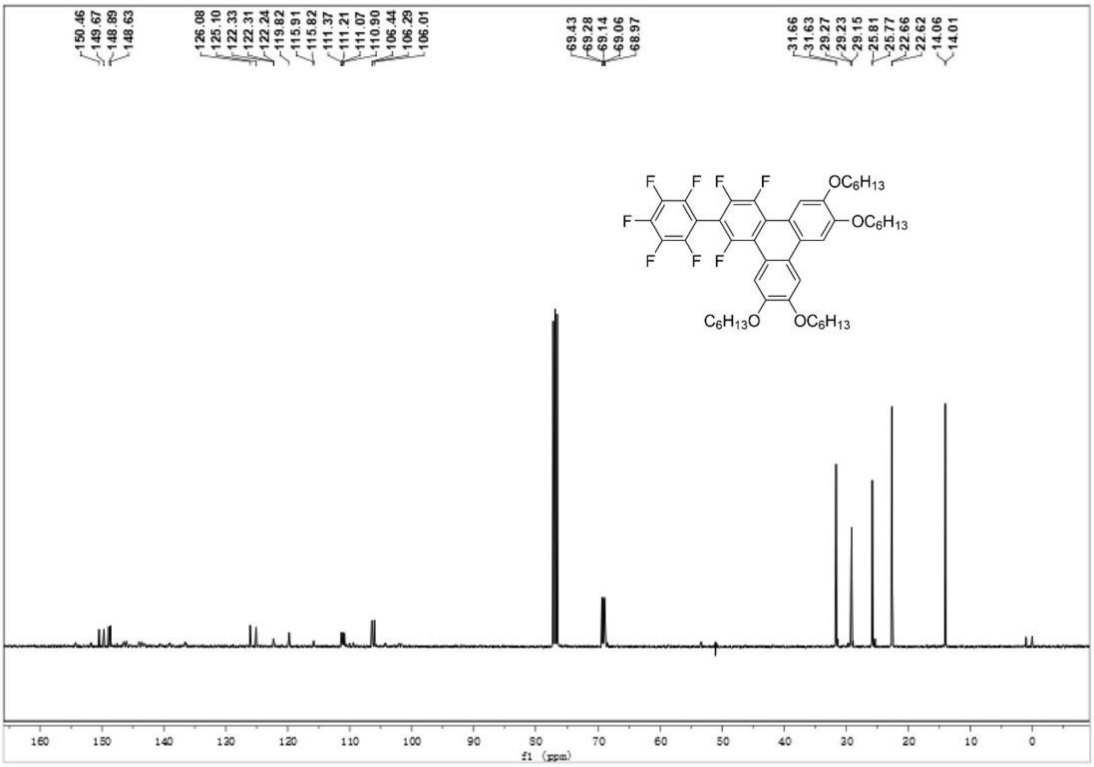

Figure S11. ^{1}H NMR ($CDCl_3$, 400 MHz), ^{19}F NMR ($CDCl_3$, 376 MHz) and ^{13}C NMR ($CDCl_3$, 101 MHz) spectra of **F**6.

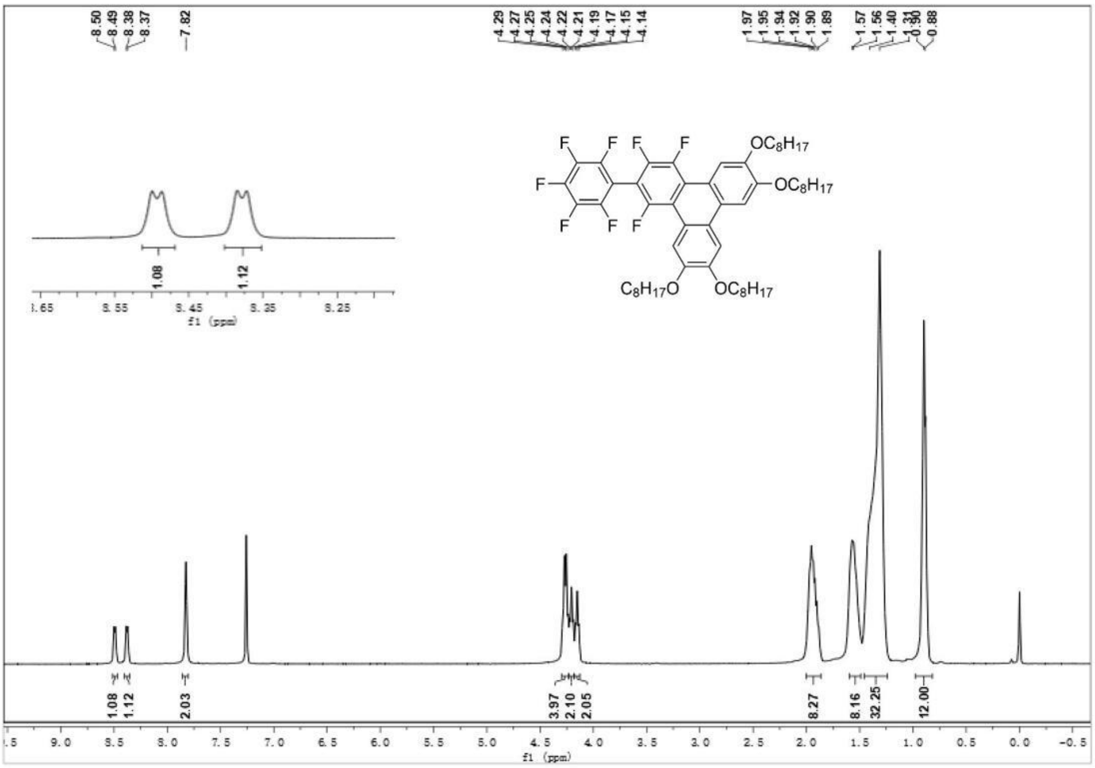
8.50
8.49
8.38
8.37
7.82
4.29
4.27
4.25
4.24
4.22
4.21
4.19
4.17
4.15
4.14
1.97
1.95
1.94
1.92
1.90
1.89
1.57
1.56
1.40
1.31
0.90
0.88
F
OC8H17
C8H17O
1.08
1.12
2.03
3.97
2.10
2.05
8.27
8.16
32.25
12.00
f1 (ppm)

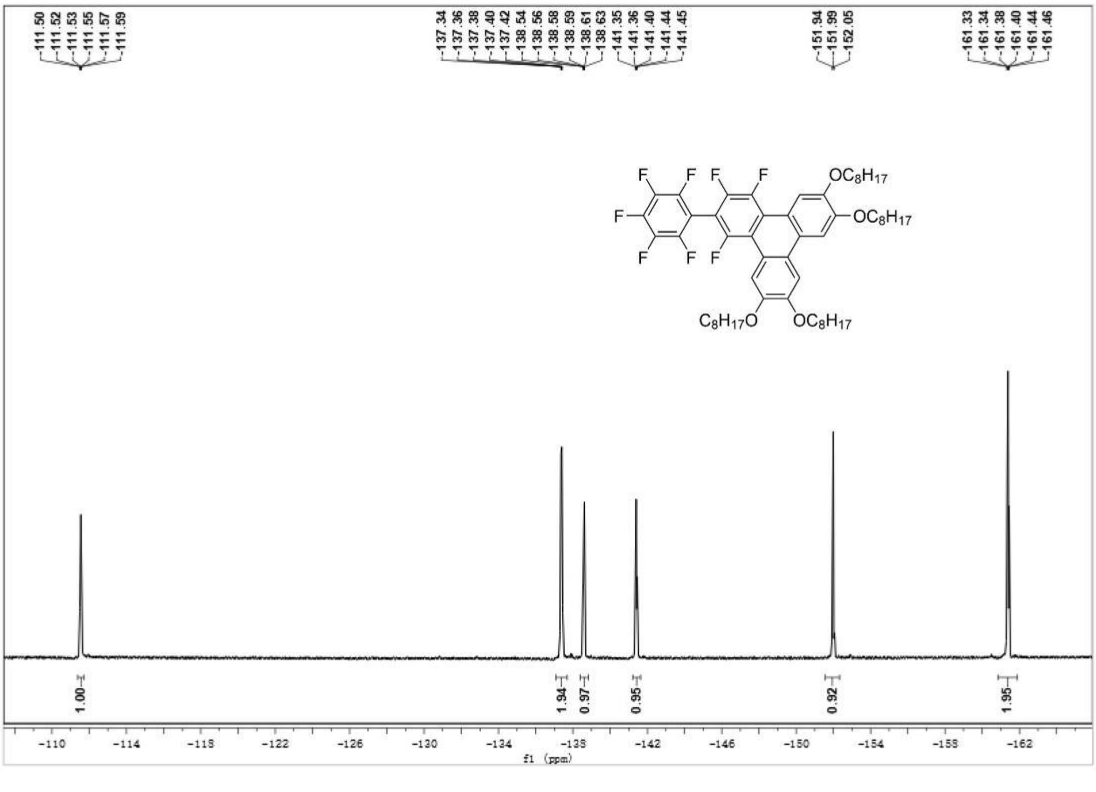
111.50
111.52
111.53
111.55
111.57
111.59
137.34
137.36
137.38
137.40
137.42
138.54
138.56
138.58
138.59
138.61
138.63
141.35
141.36
141.40
141.44
141.45
151.94
151.99
152.05
161.33
161.34
161.38
161.40
161.44
161.46
F
OC8H17
C8H17O
1.00
1.94
0.97
0.95
0.92
1.95
f1 (ppm)

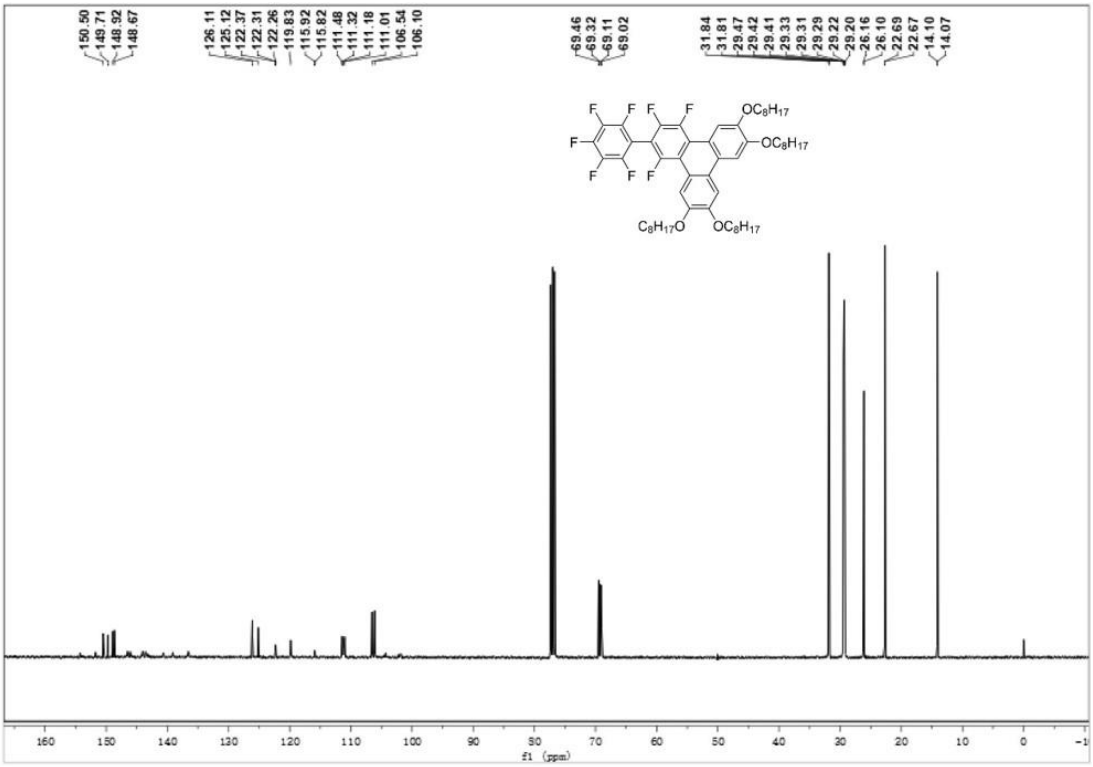

Figure S12. ^{1}H NMR ($CDCl_3$, 400 MHz), ^{19}F NMR ($CDCl_3$, 376 MHz) and ^{13}C NMR ($CDCl_3$, 101 MHz) spectra of **F**8.

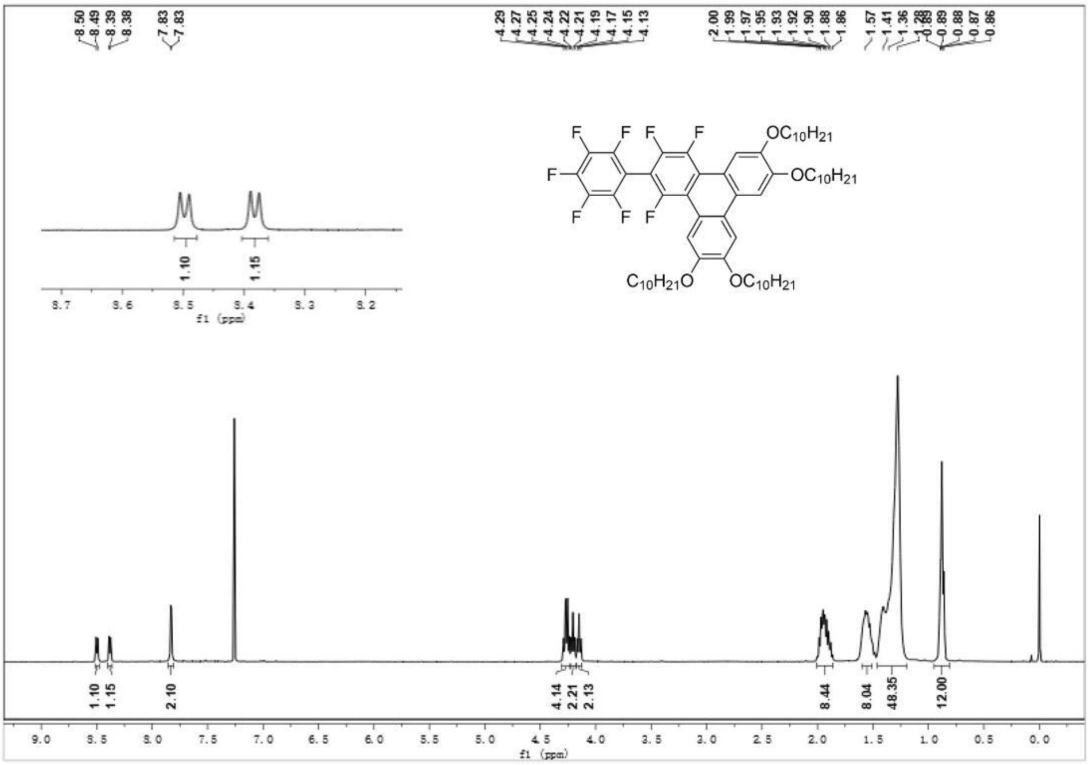

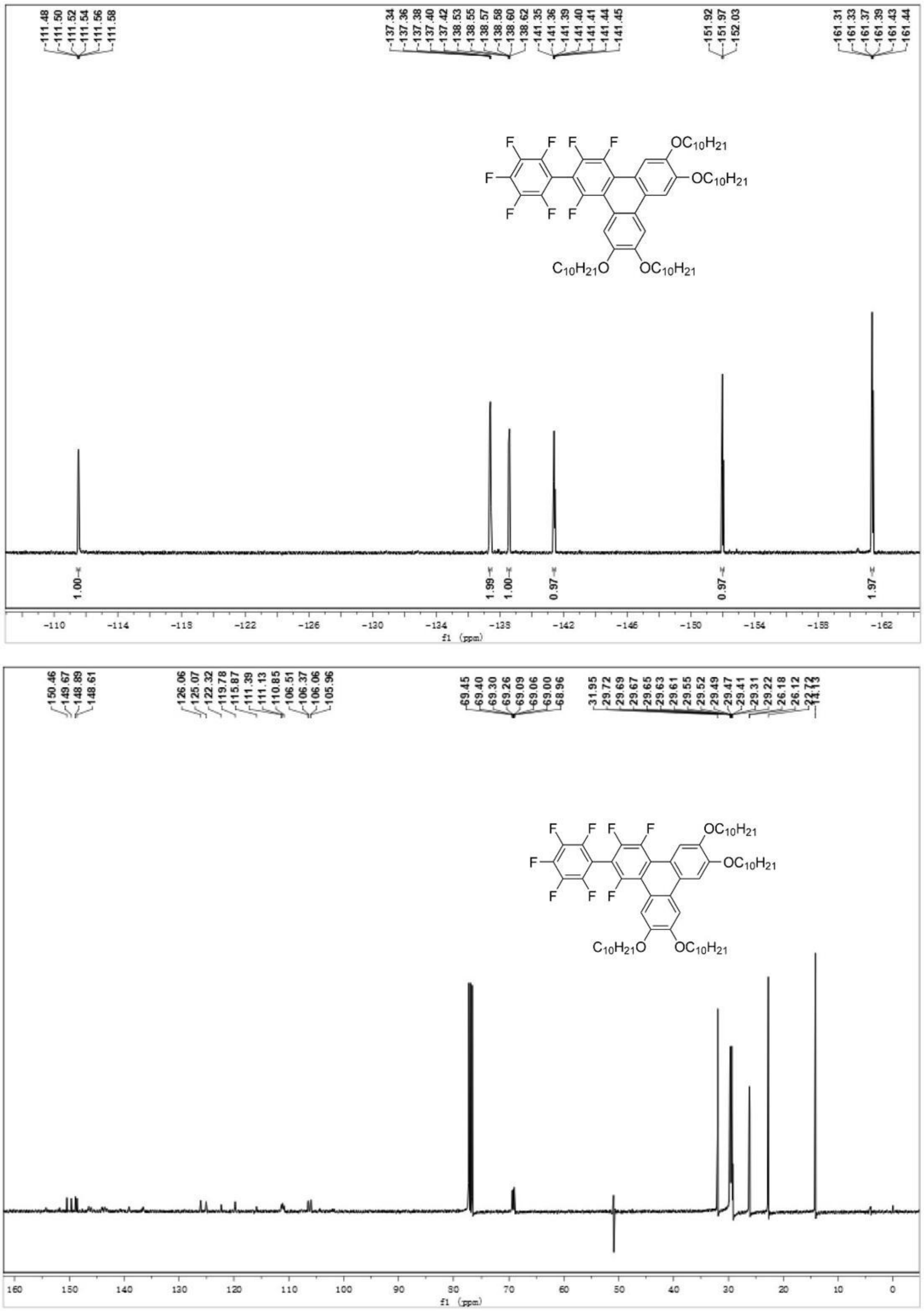

Figure S13. ^{1}H NMR ($CDCl_3$, 400 MHz), ^{19}F NMR ($CDCl_3$, 376 MHz) and ^{13}C NMR ($CDCl_3$, 101 MHz) spectra of **F**10.

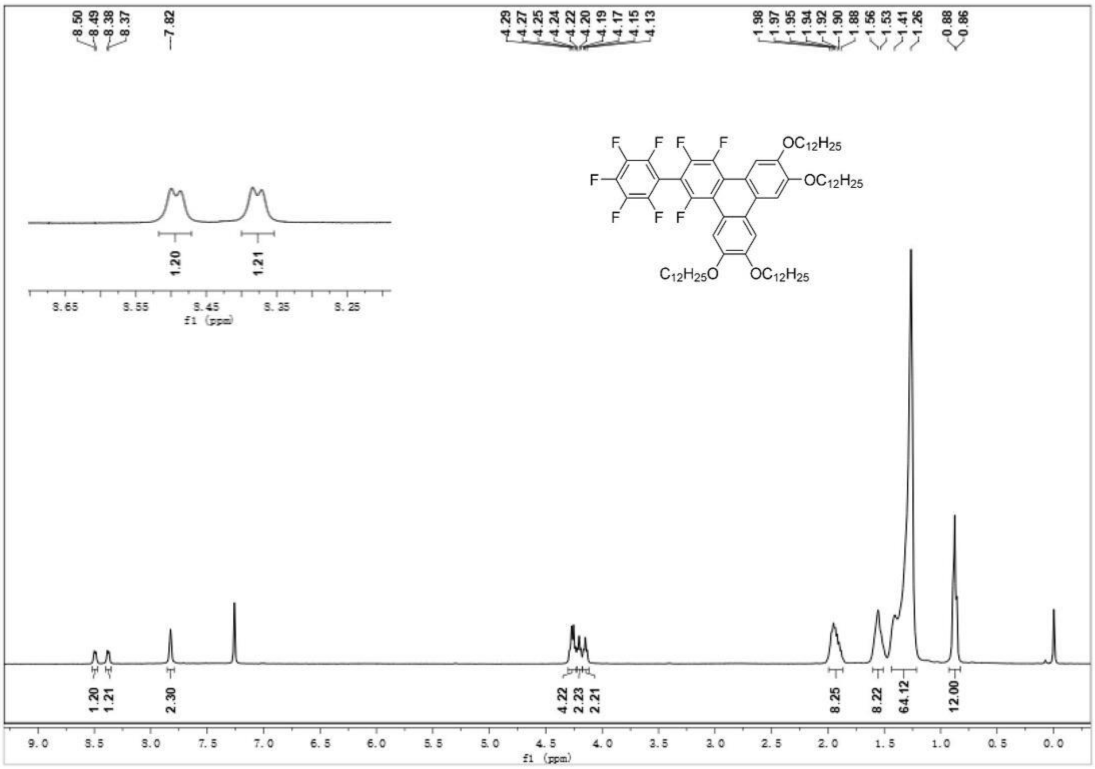

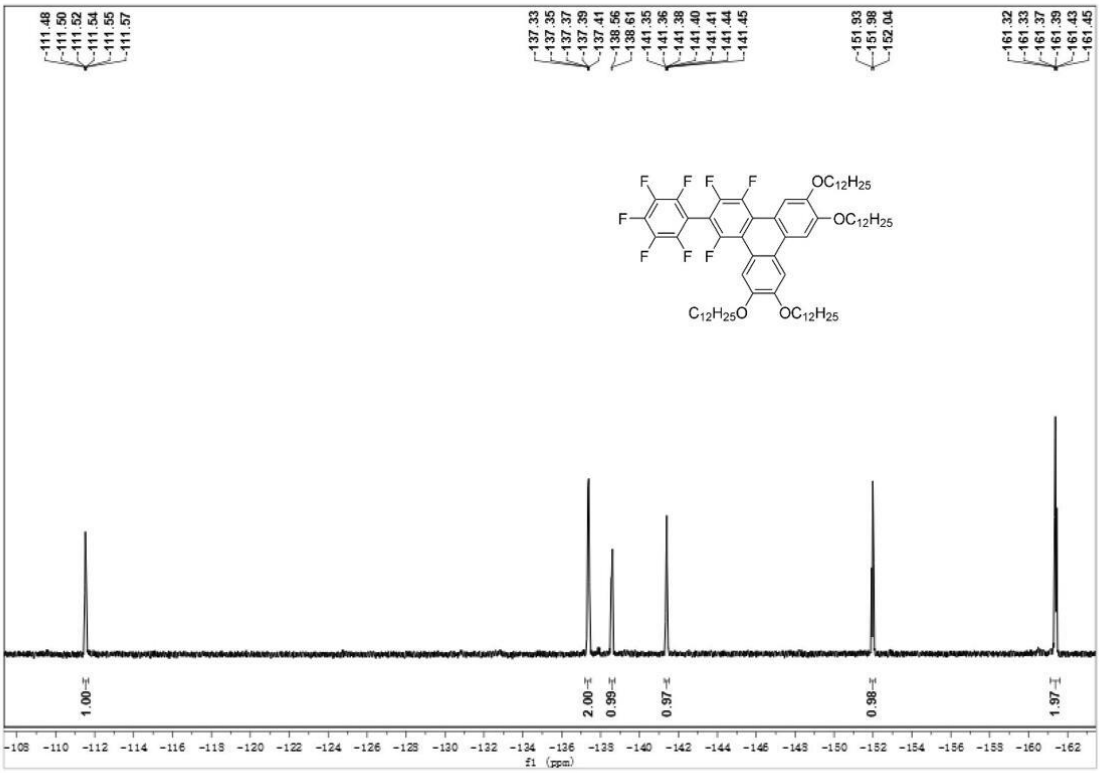

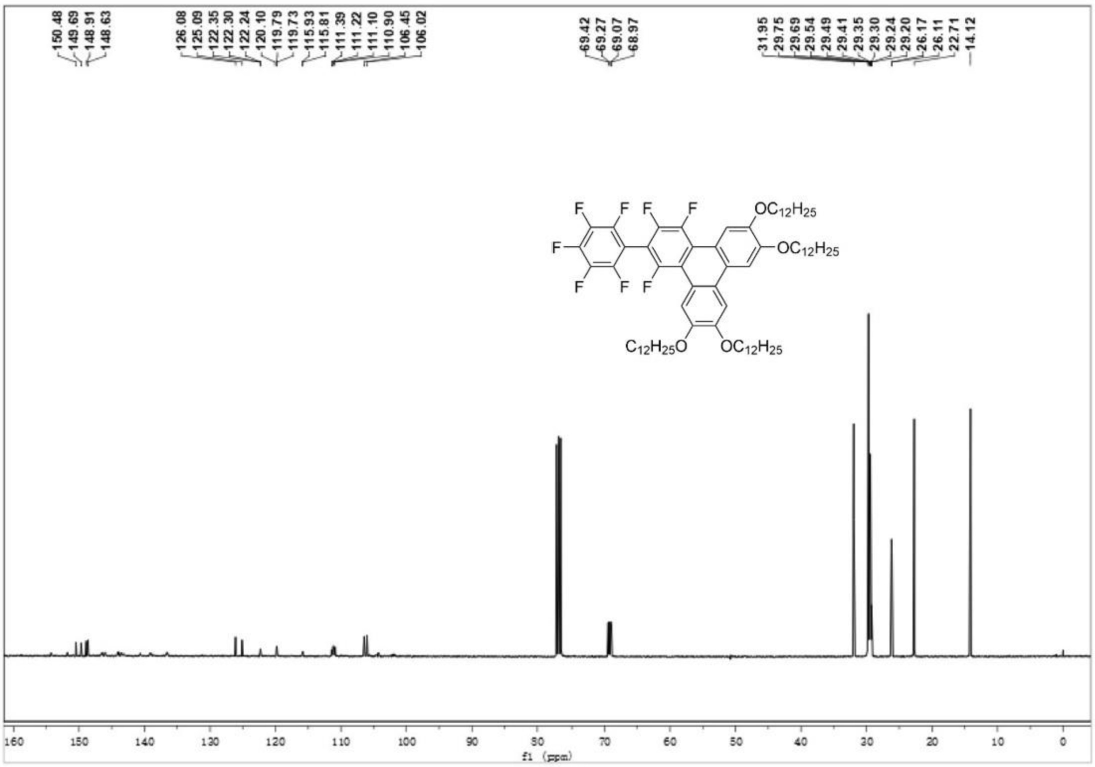

Figure S14. ^{1}H NMR ($CDCl_3$, 400 MHz), ^{19}F NMR ($CDCl_3$, 376 MHz) and ^{13}C NMR ($CDCl_3$, 101 MHz) spectra of **F**12.

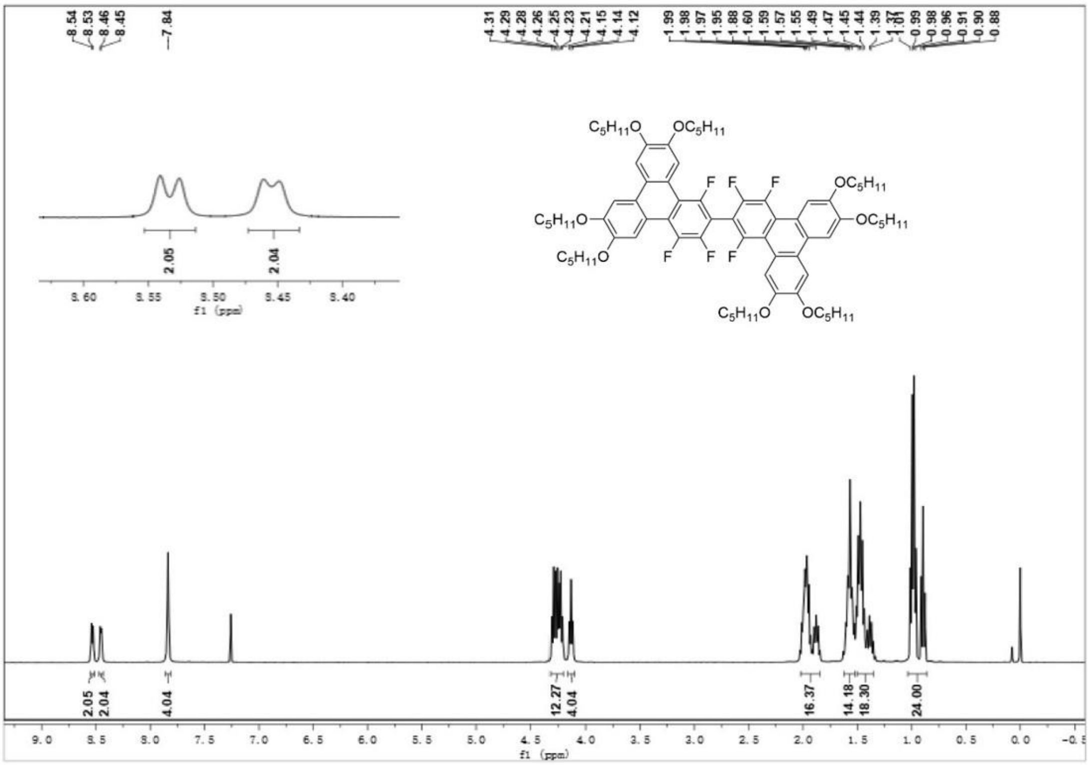

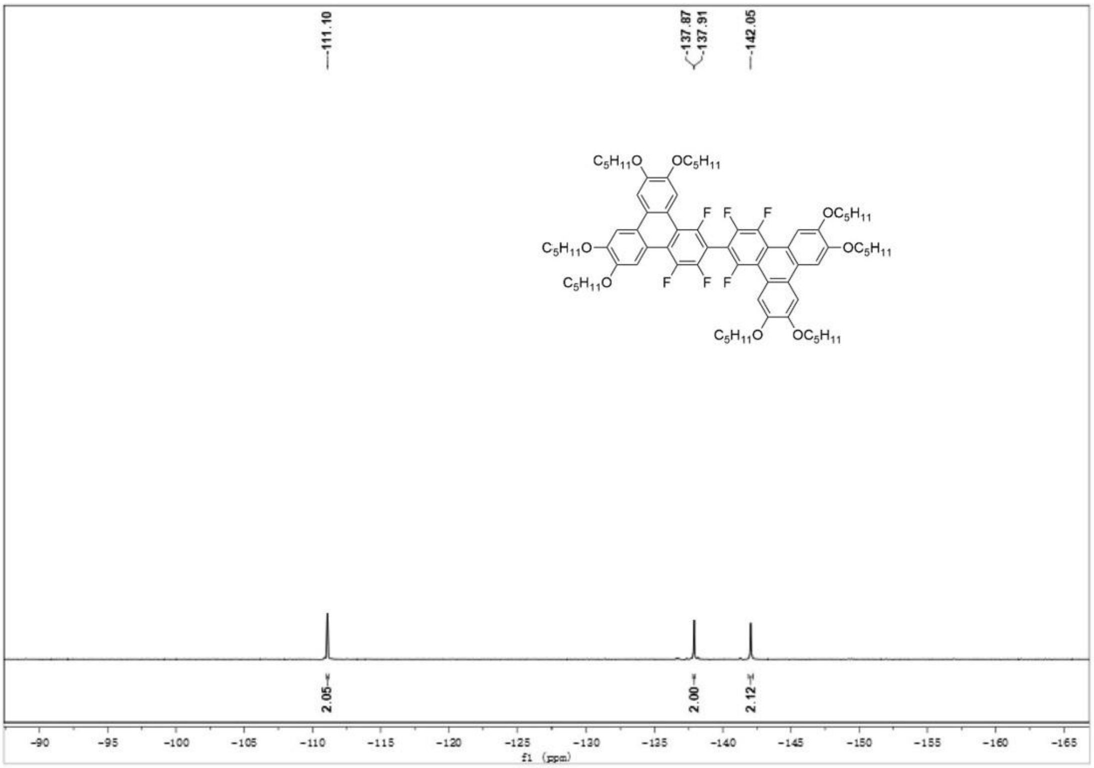

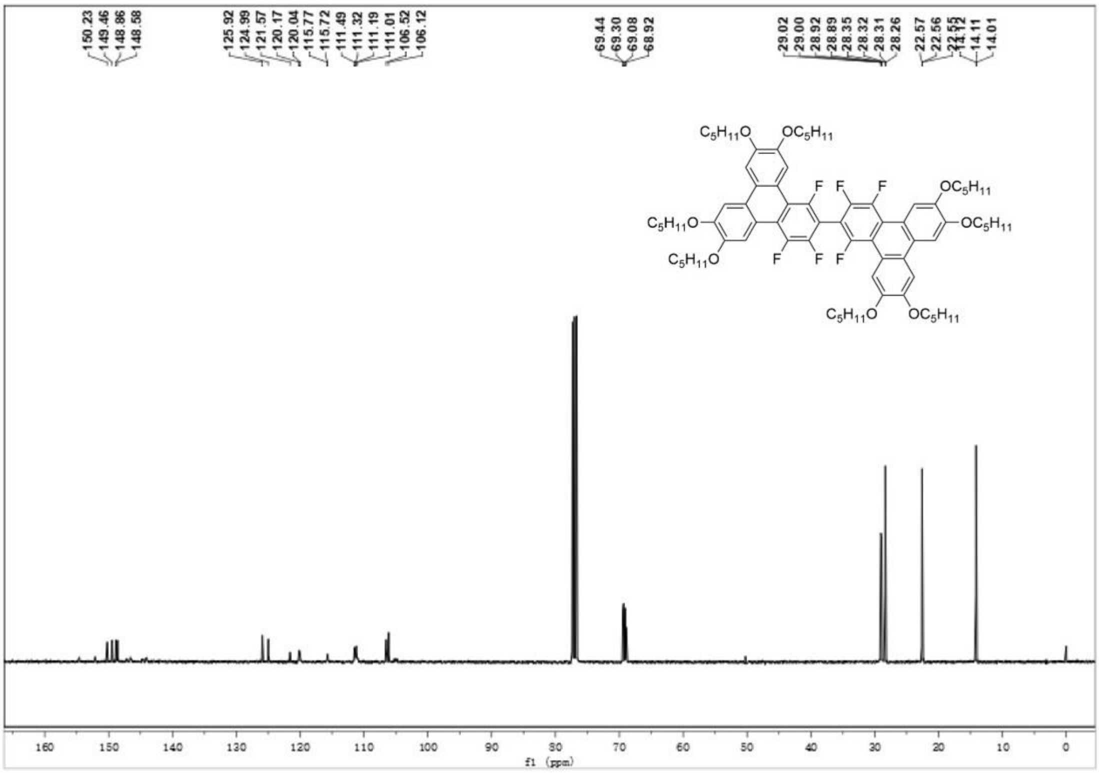

Figure S15. ^{1}H NMR ($CDCl_3$, 400 MHz), ^{19}F NMR ($CDCl_3$, 376 MHz) and ^{13}C NMR ($CDCl_3$, 101 MHz) spectra of **G**55.

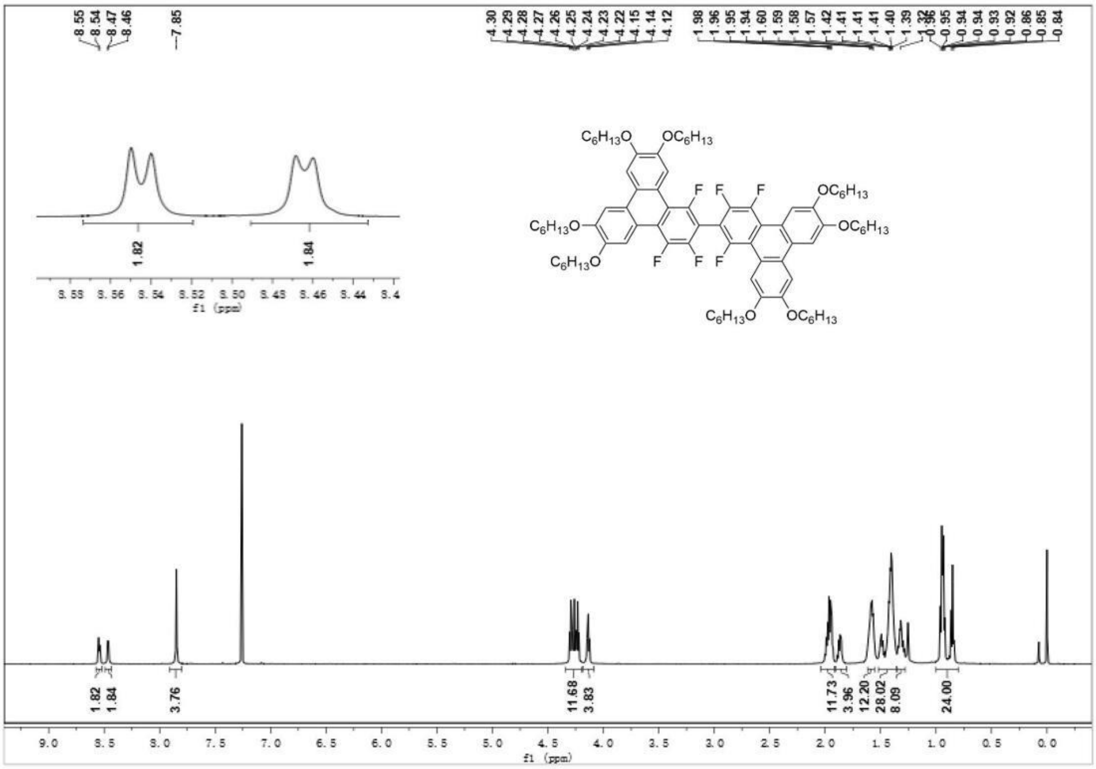

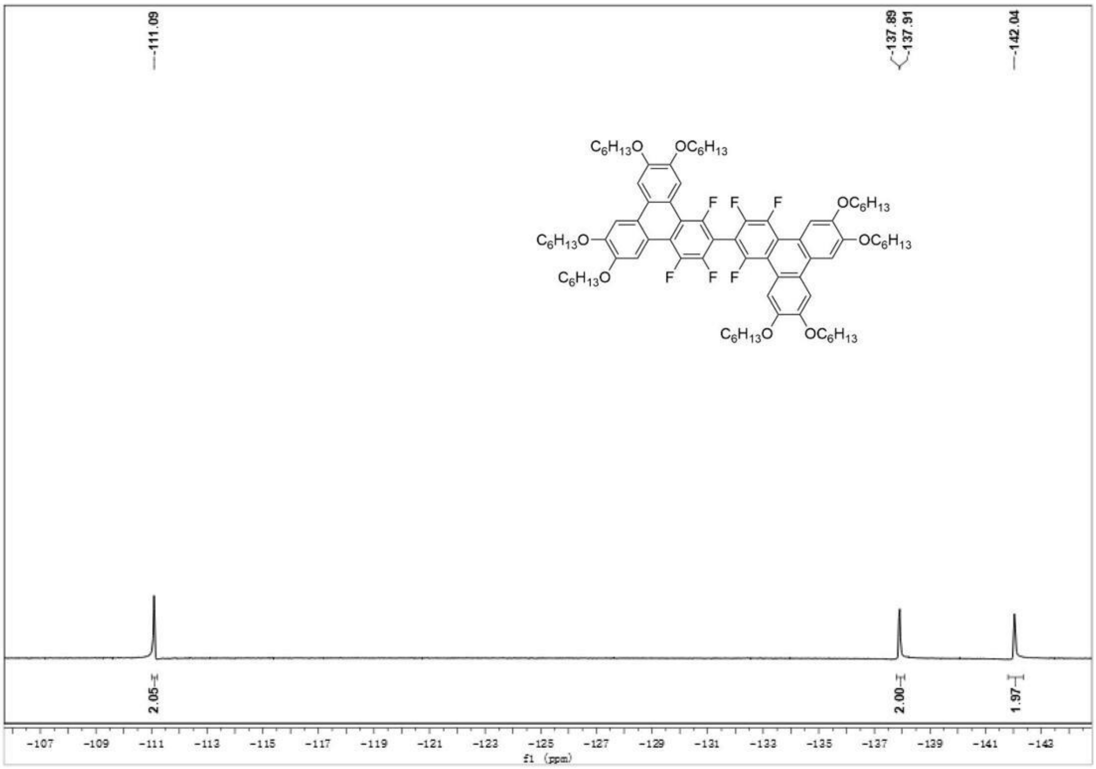

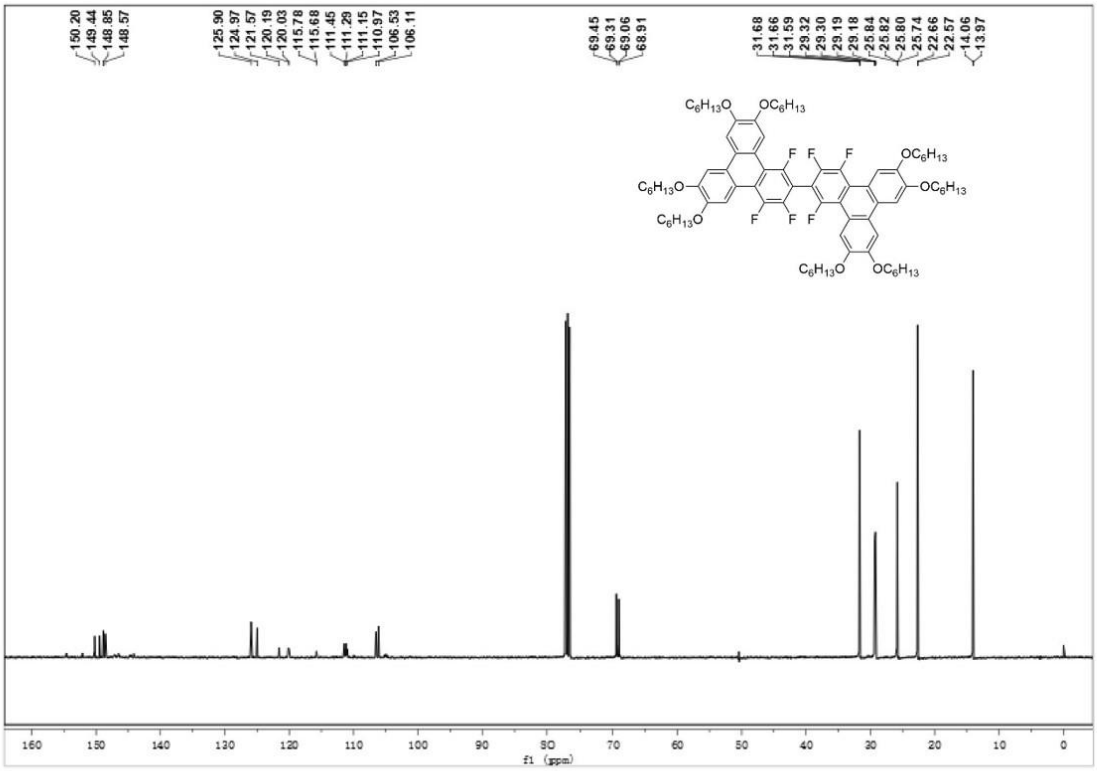

Figure S16. ^{1}H NMR ($CDCl_3$, 400 MHz), ^{19}F NMR ($CDCl_3$, 376 MHz) and ^{13}C NMR ($CDCl_3$, 101 MHz) spectra of **G**66.

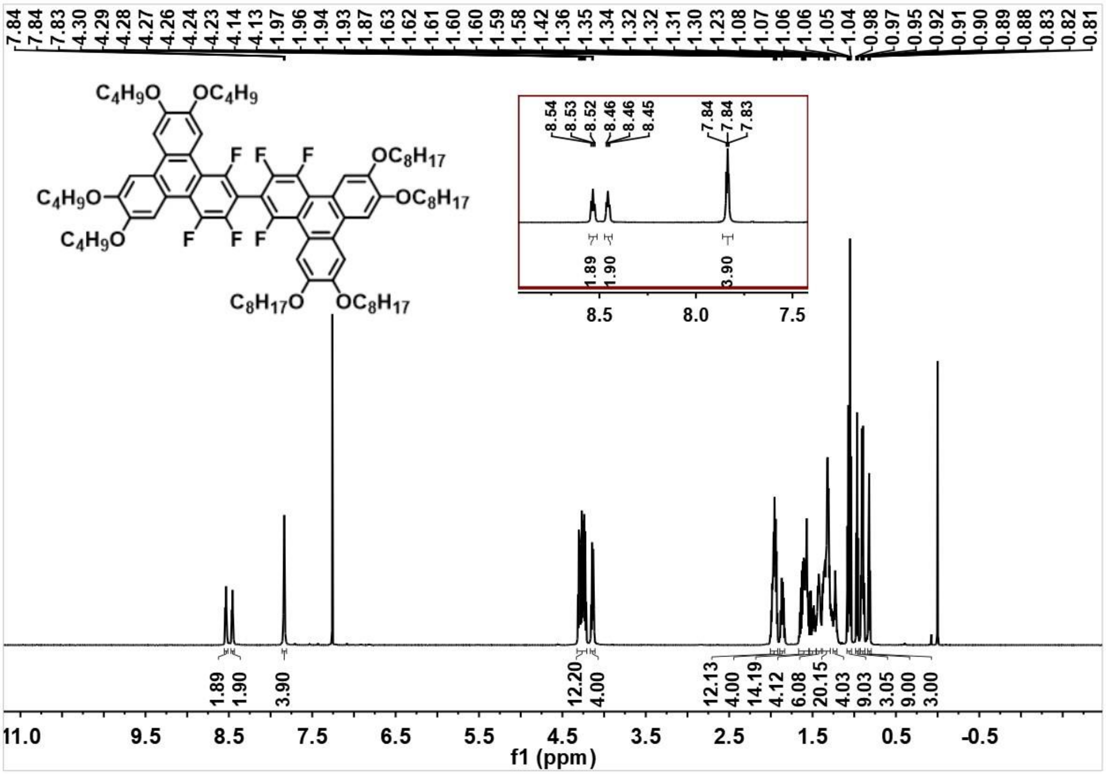

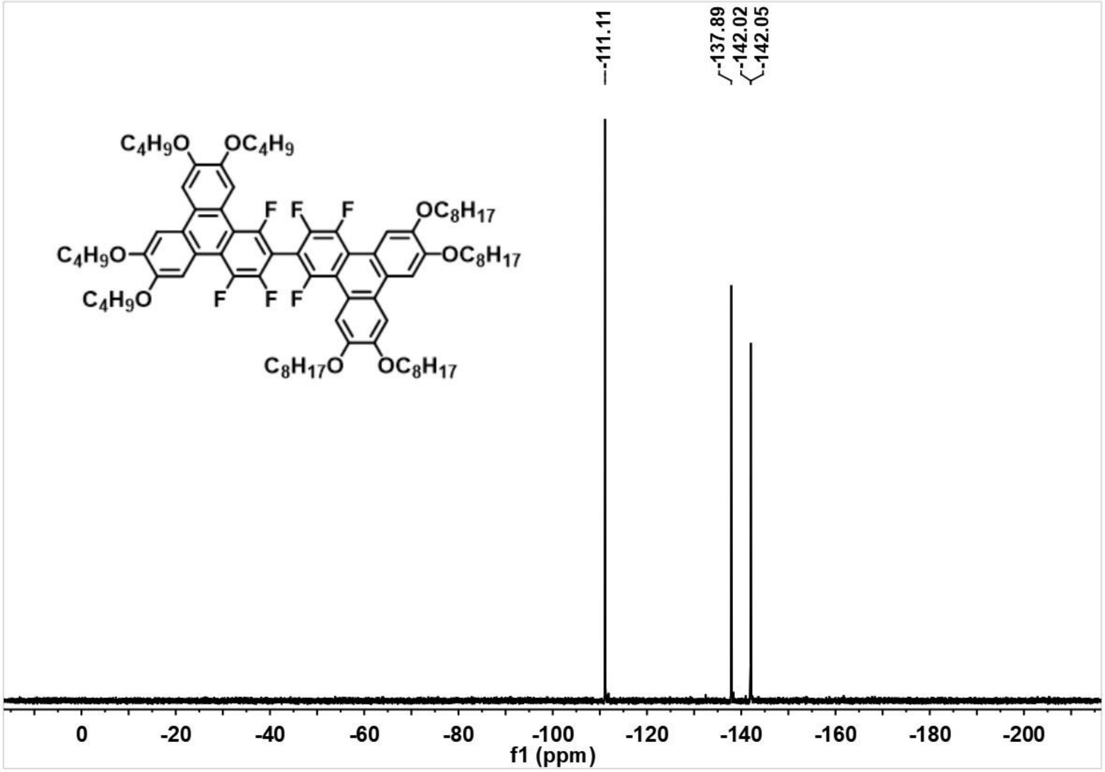

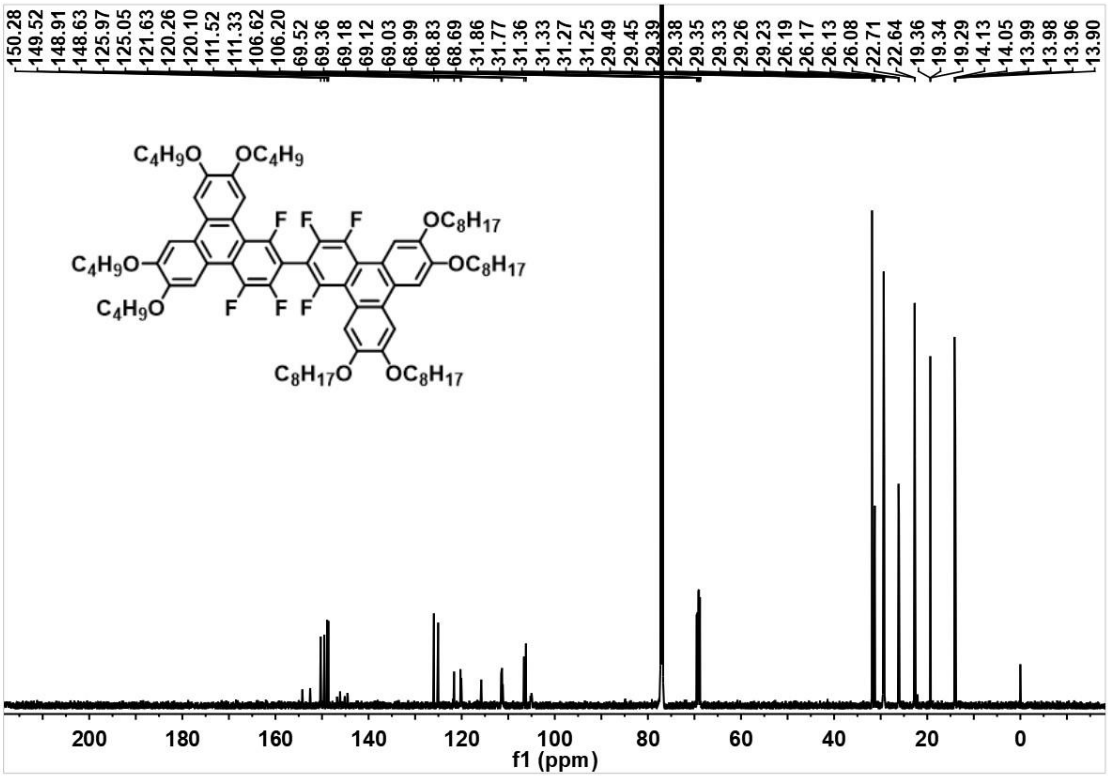

Figure S17. ^{1}H NMR ($CDCl_3$, 400 MHz), ^{19}F NMR ($CDCl_3$, 376 MHz) and ^{13}C NMR ($CDCl_3$, 101 MHz) spectra of **G**48.

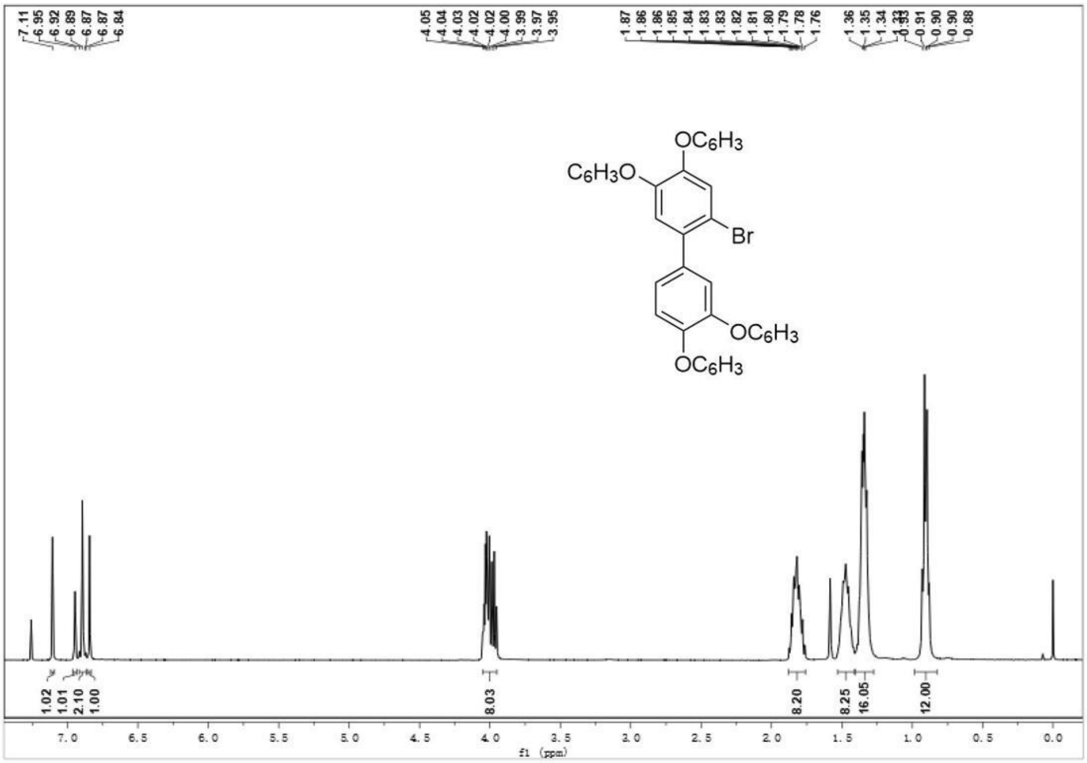

Figure S18. ^{1}H NMR ($CDCl_3$, 400 MHz) of Br-**BP**6.

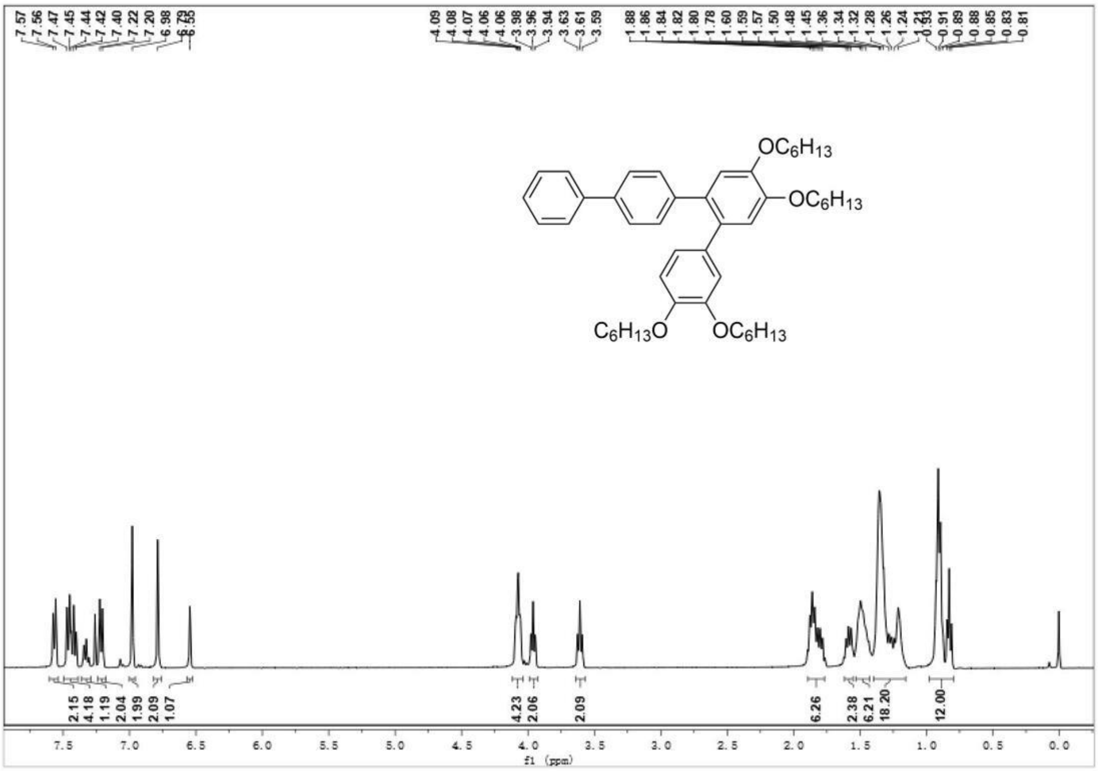

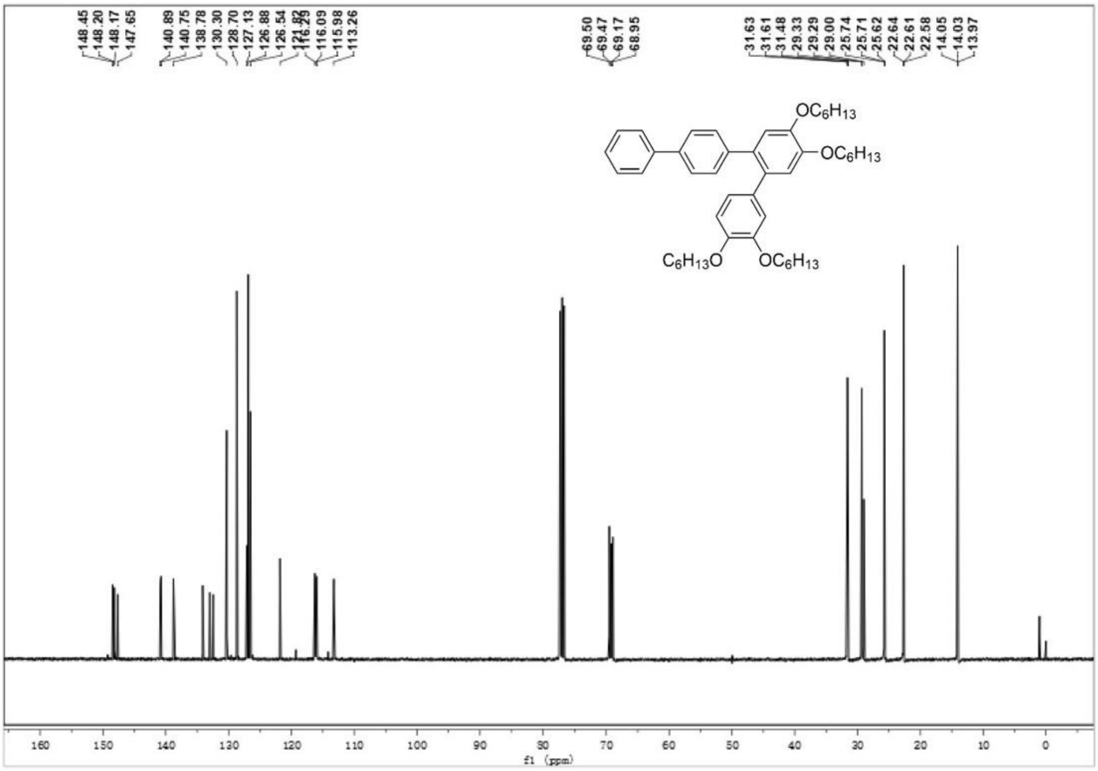

Figure S19. ^{1}H NMR ($CDCl_3$, 400 MHz) and ^{13}C NMR ($CDCl_3$, 101 MHz) of **QP**6.

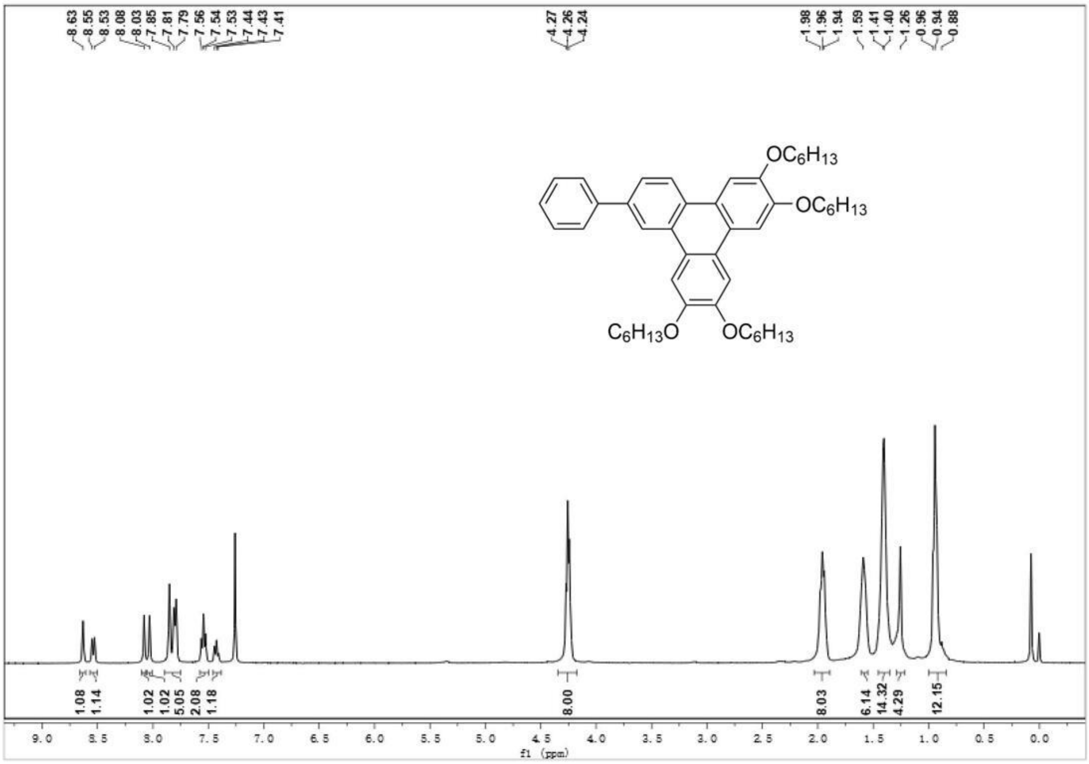

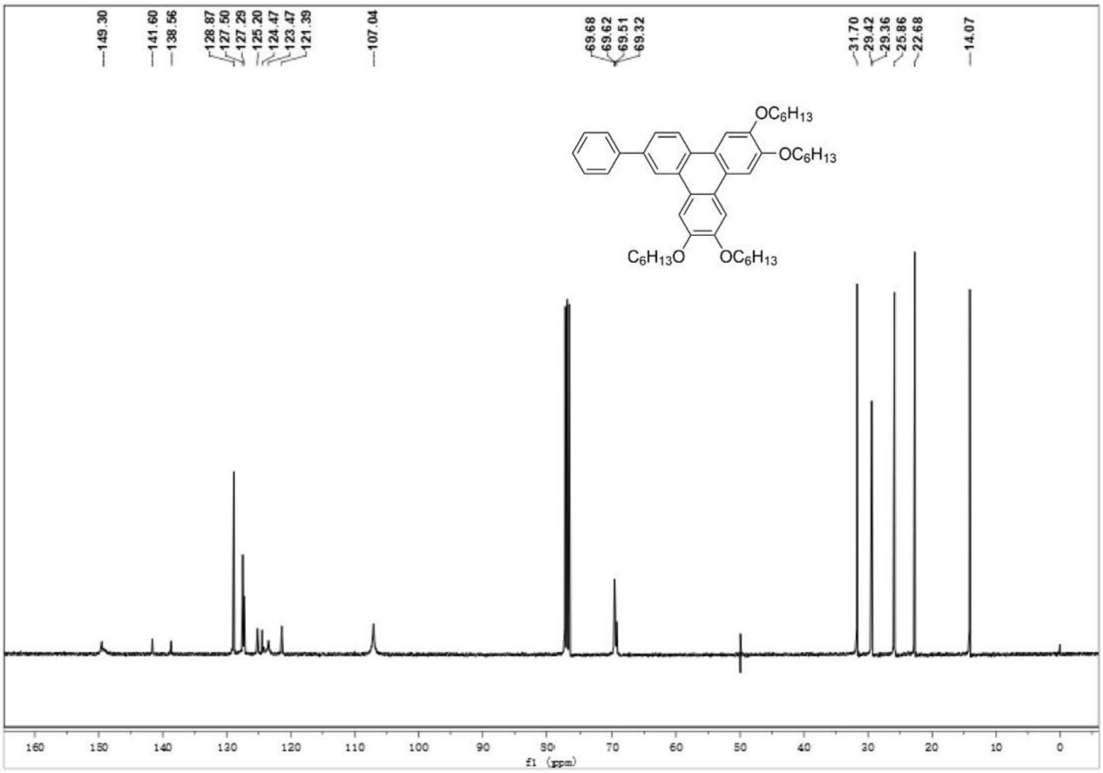

Figure S20. ^{1}H NMR ($CDCl_3$, 400 MHz) and ^{13}C NMR ($CDCl_3$, 101 MHz) of **BTP**6.

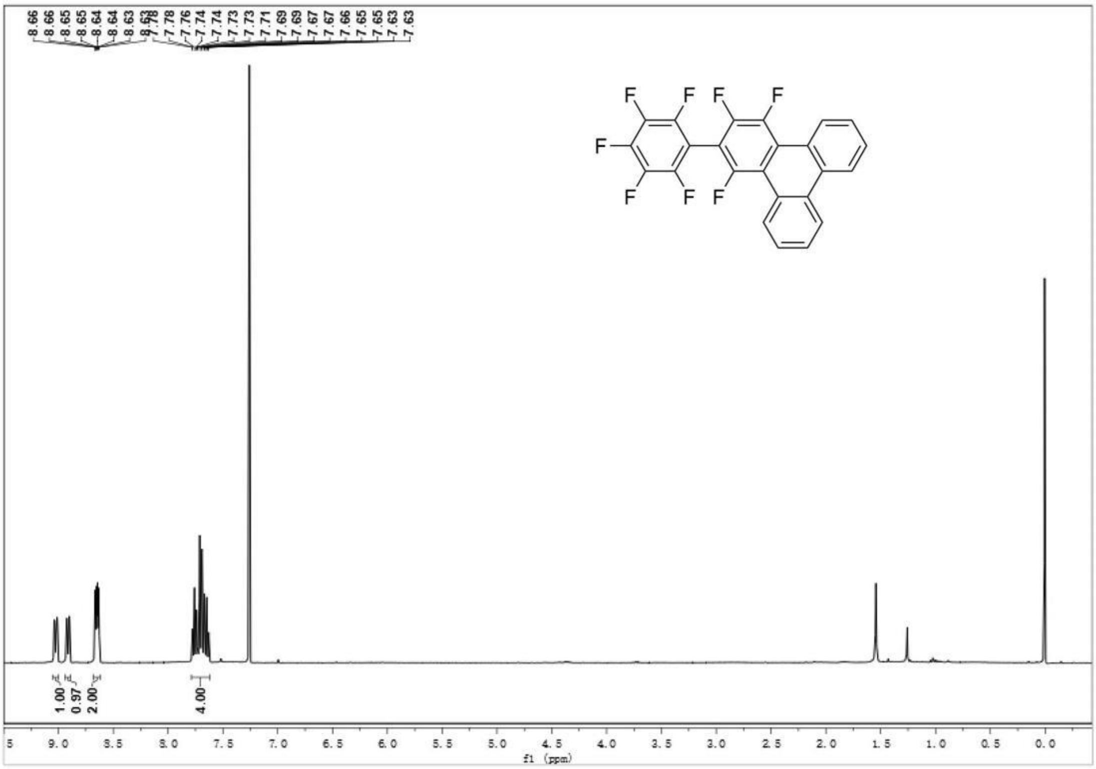

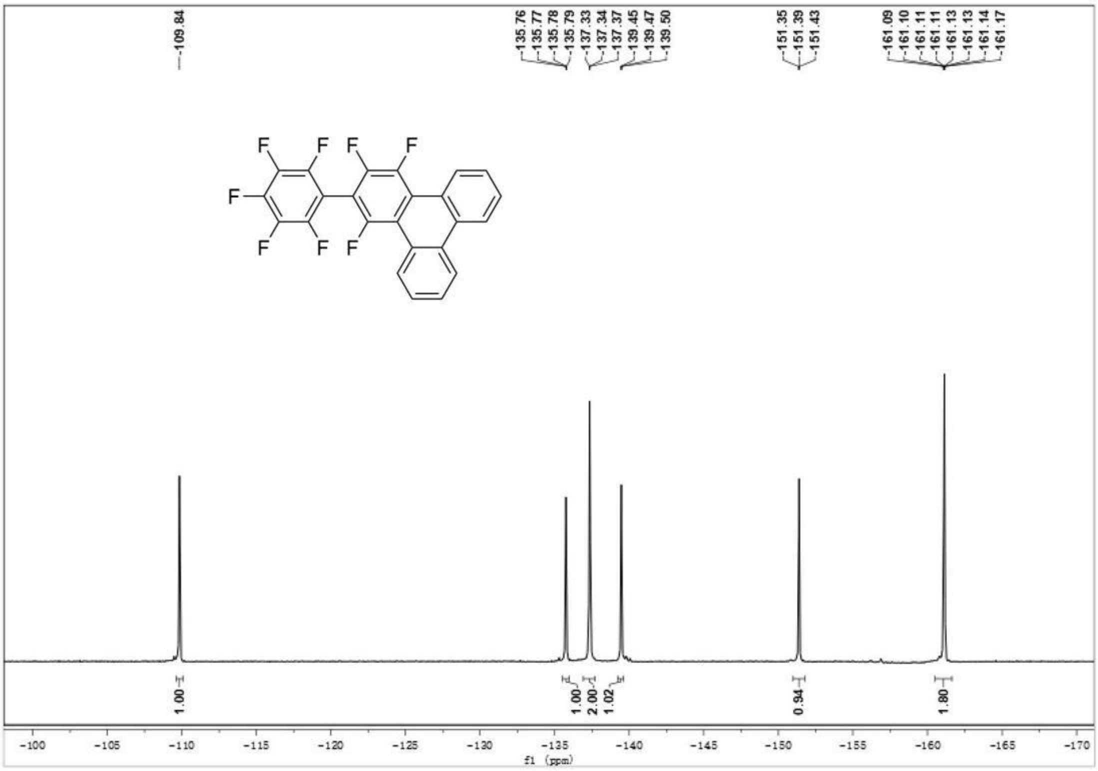

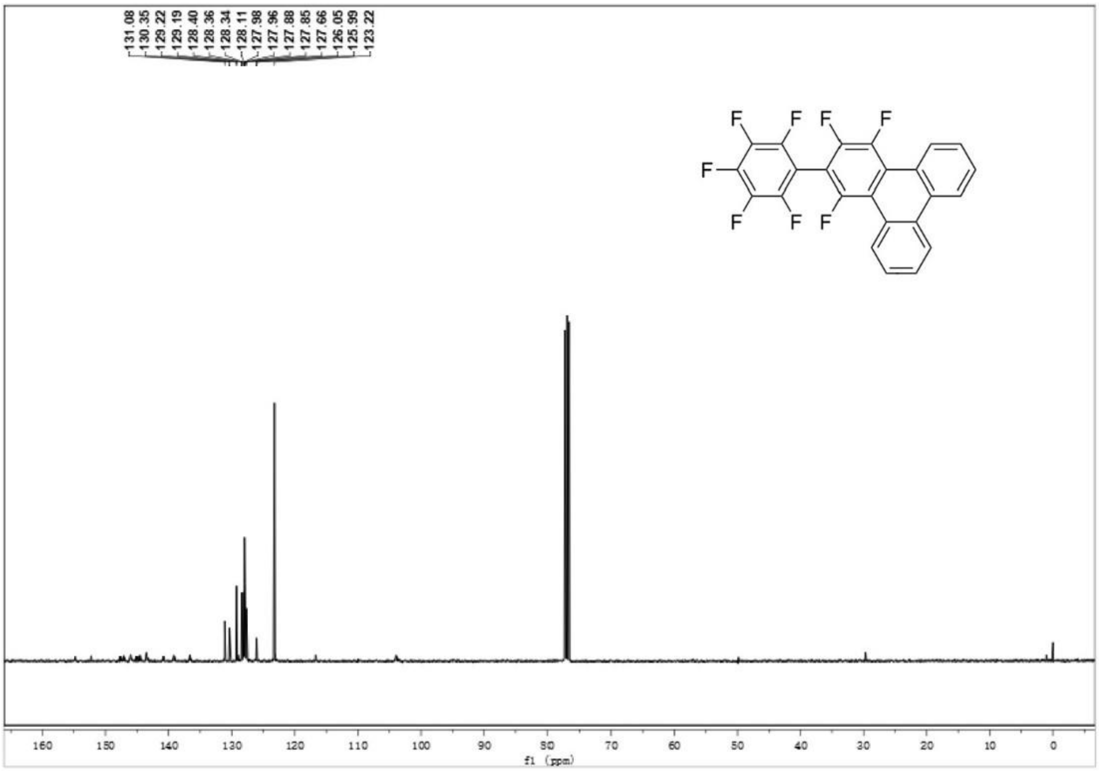

Figure S21. ^1H NMR ($CDCl_3$, 400 MHz), ^{19}F NMR ($CDCl_3$, 565 MHz) and ^{13}C NMR ($CDCl_3$, 101 MHz) of **F**.

4. HRMS

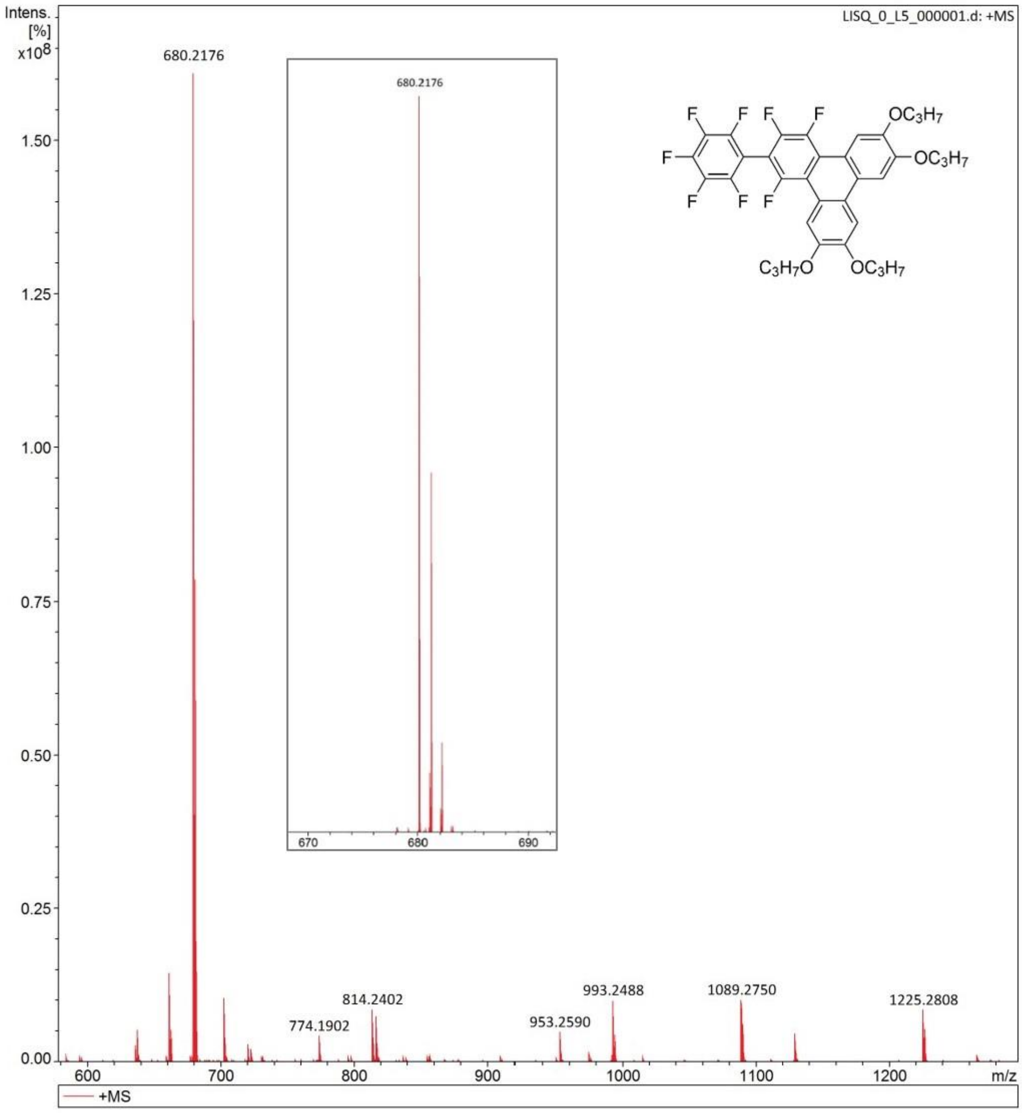

Figure S22. HRMS *m*/*z* (ESI) spectrum of **F**3.

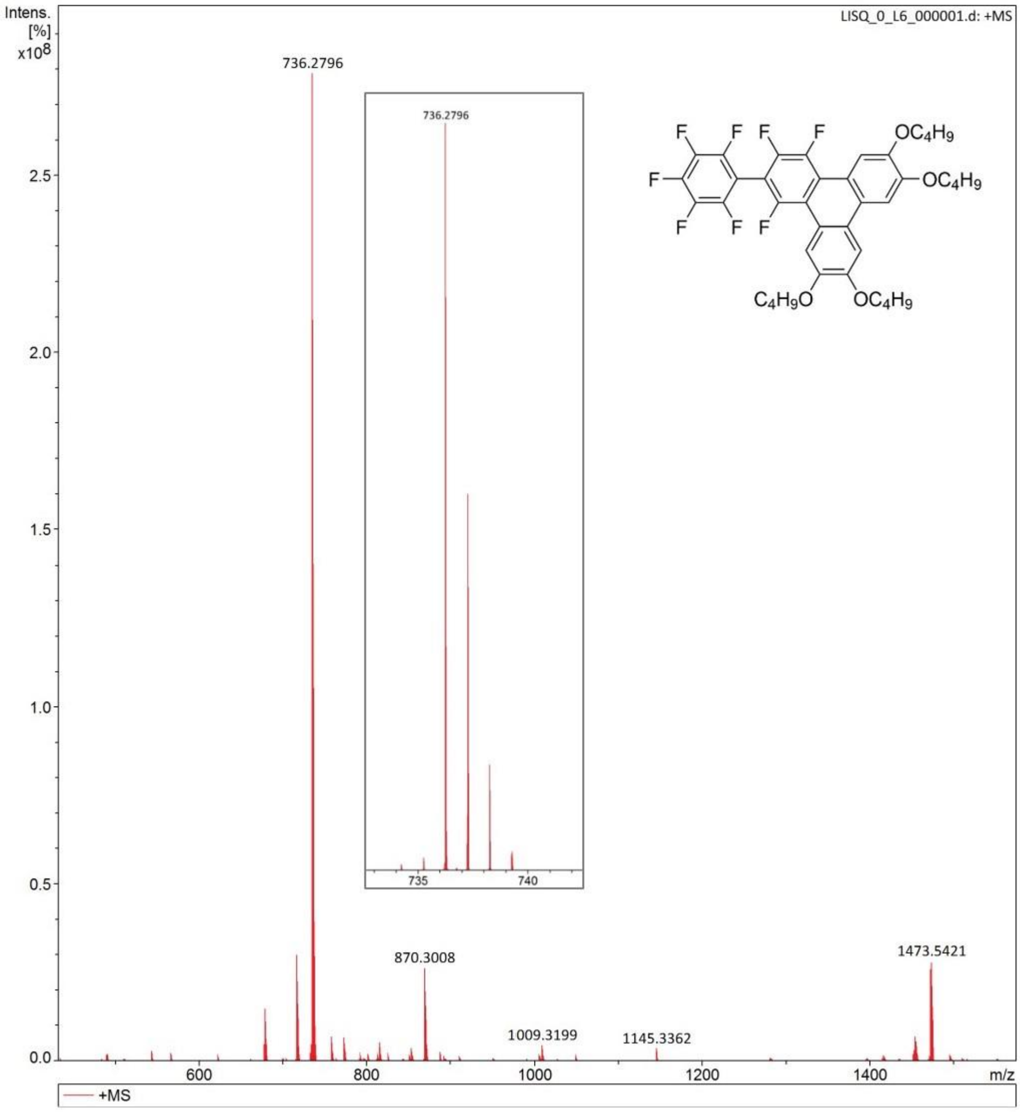

Figure S23. HRMS *m/z* (ESI) spectrum of **F**4.

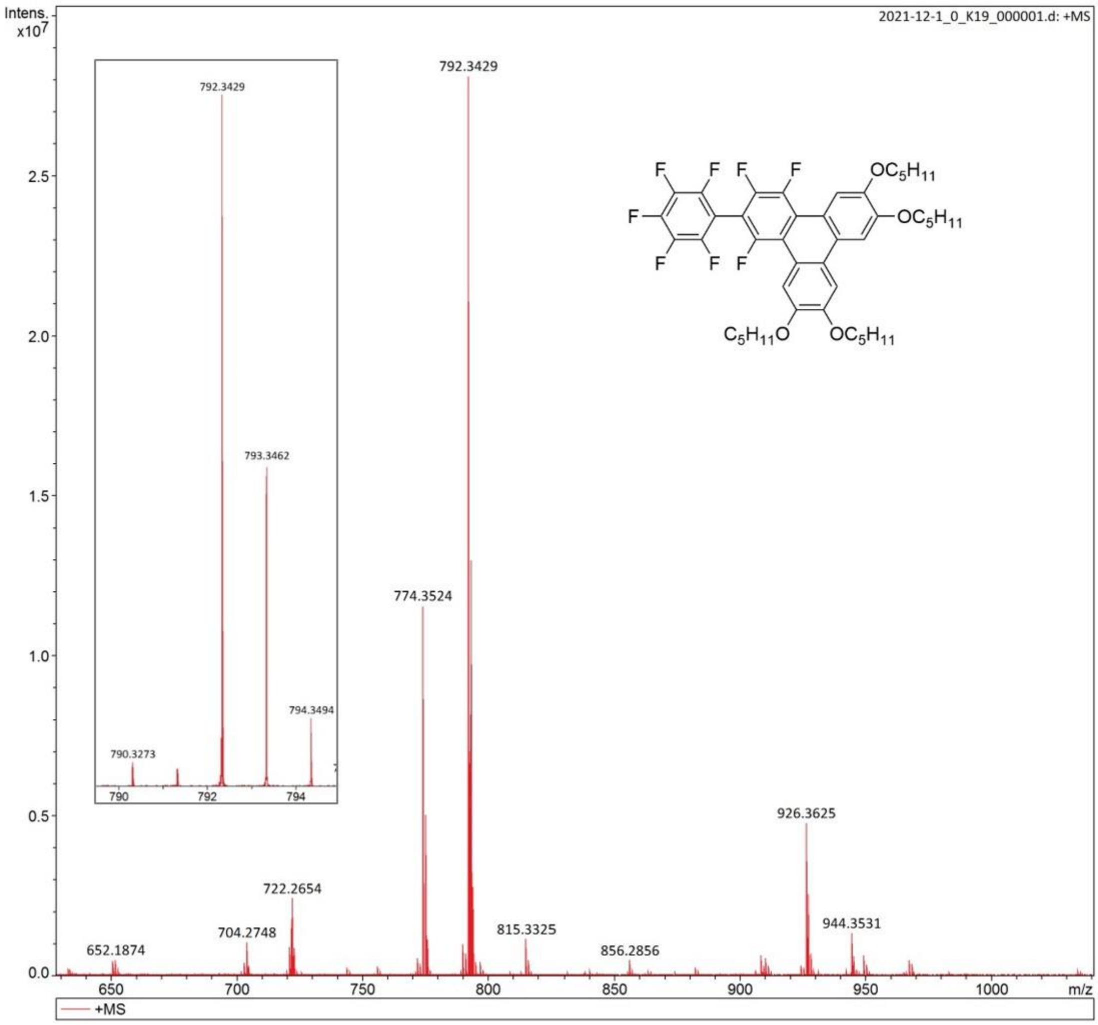

Figure S24. HRMS *m*/*z* (ESI) spectrum of **F**5.

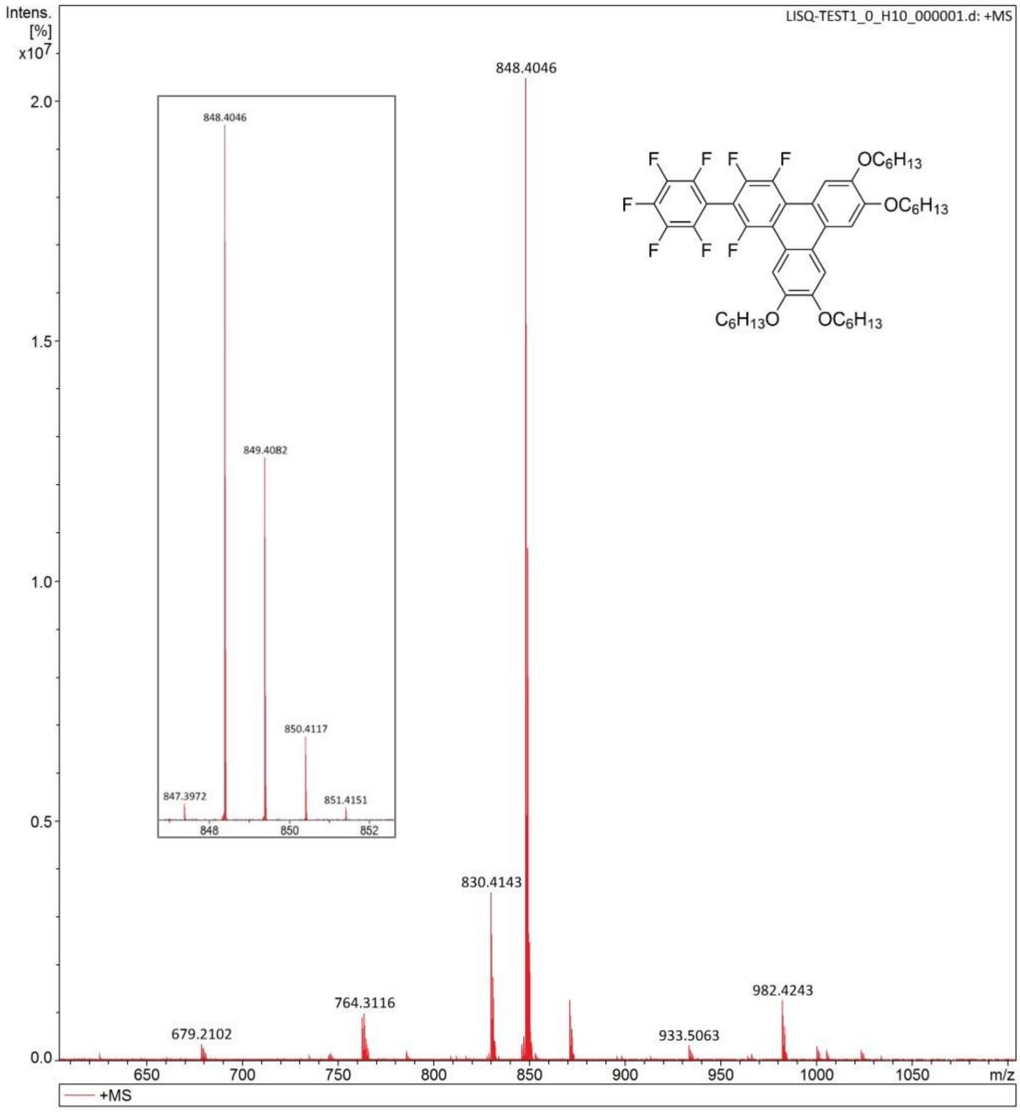

Figure S25. HRMS *m*/*z* (ESI) spectrum of **F**6.

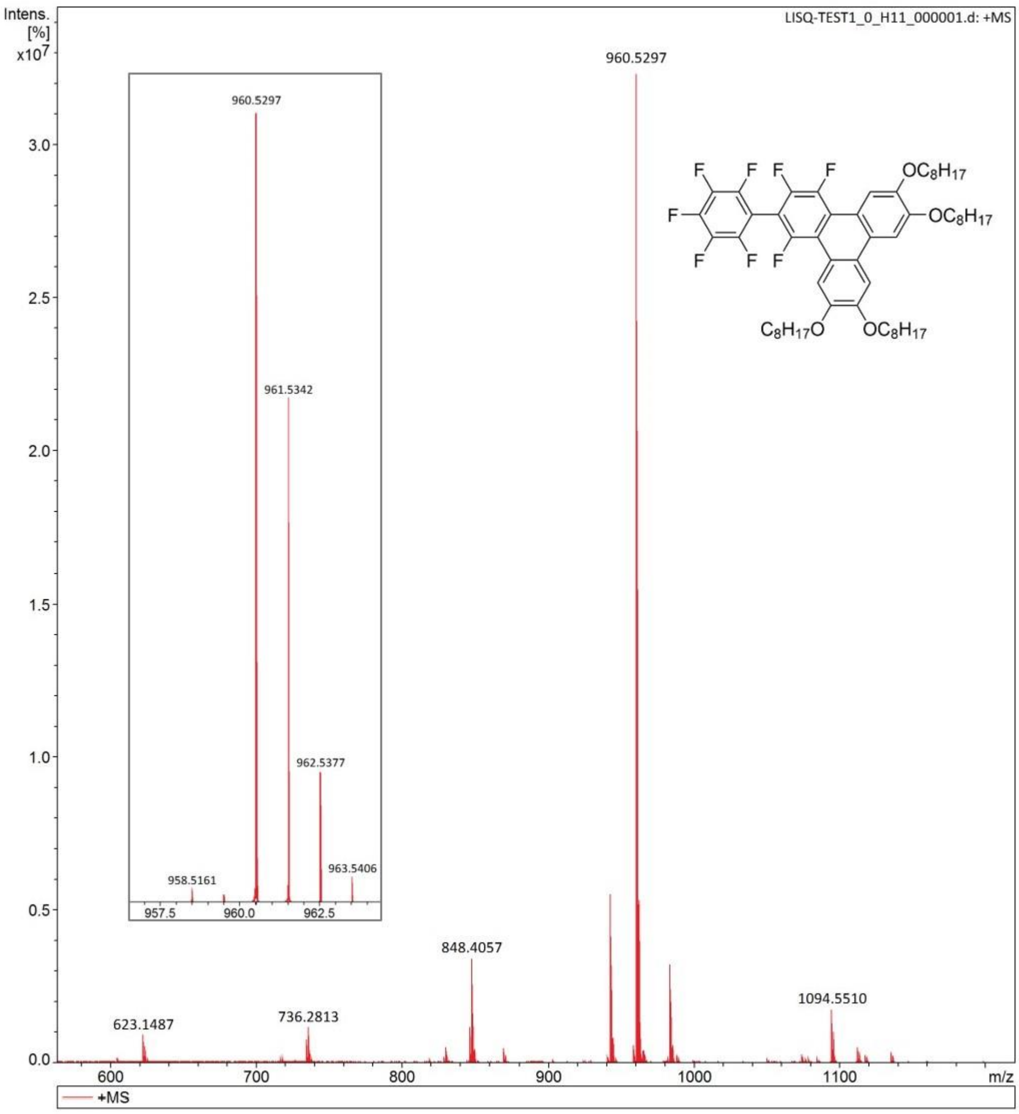

Figure S26. HRMS *m*/*z* (ESI) spectrum of **F**8.

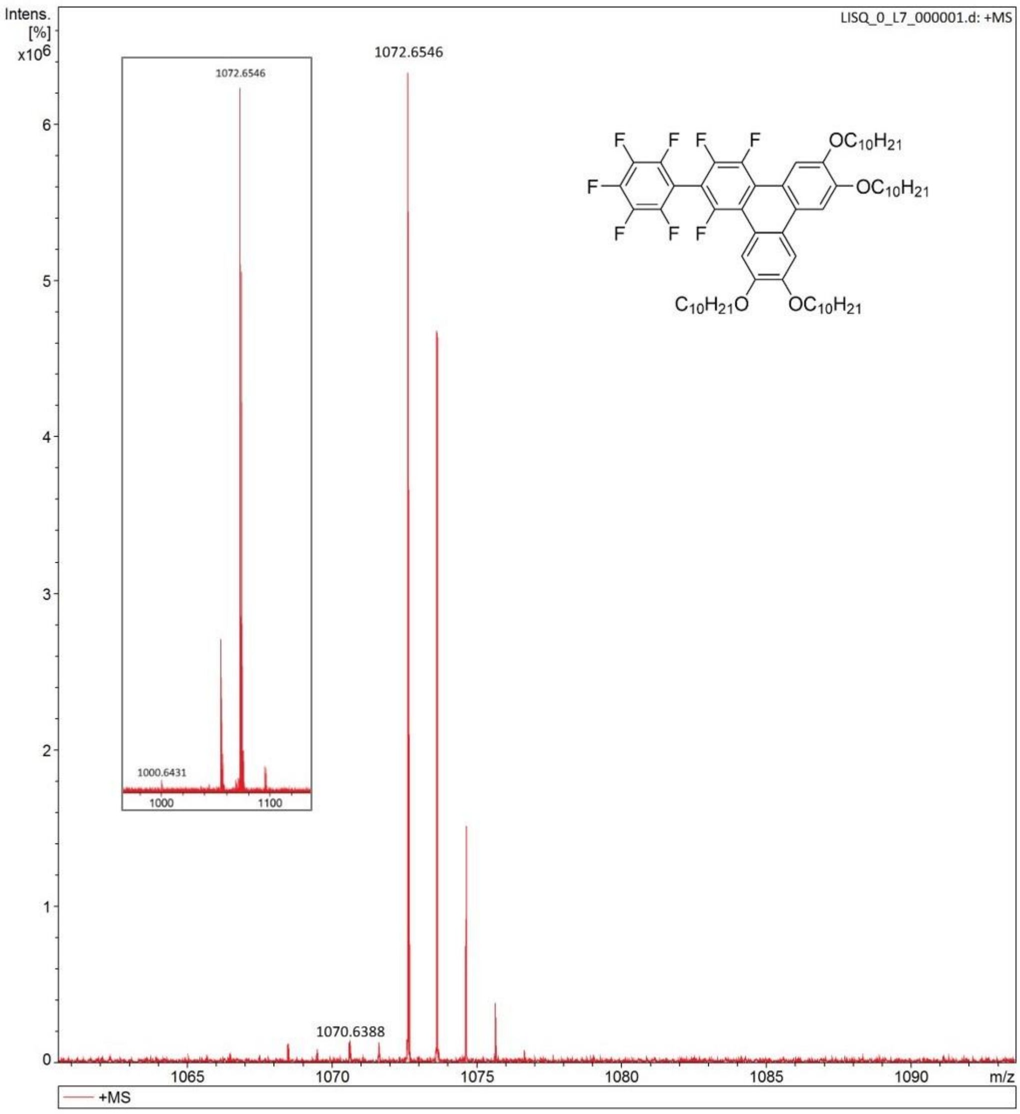

Figure S27. HRMS *m/z* (ESI) spectrum of **F**10.

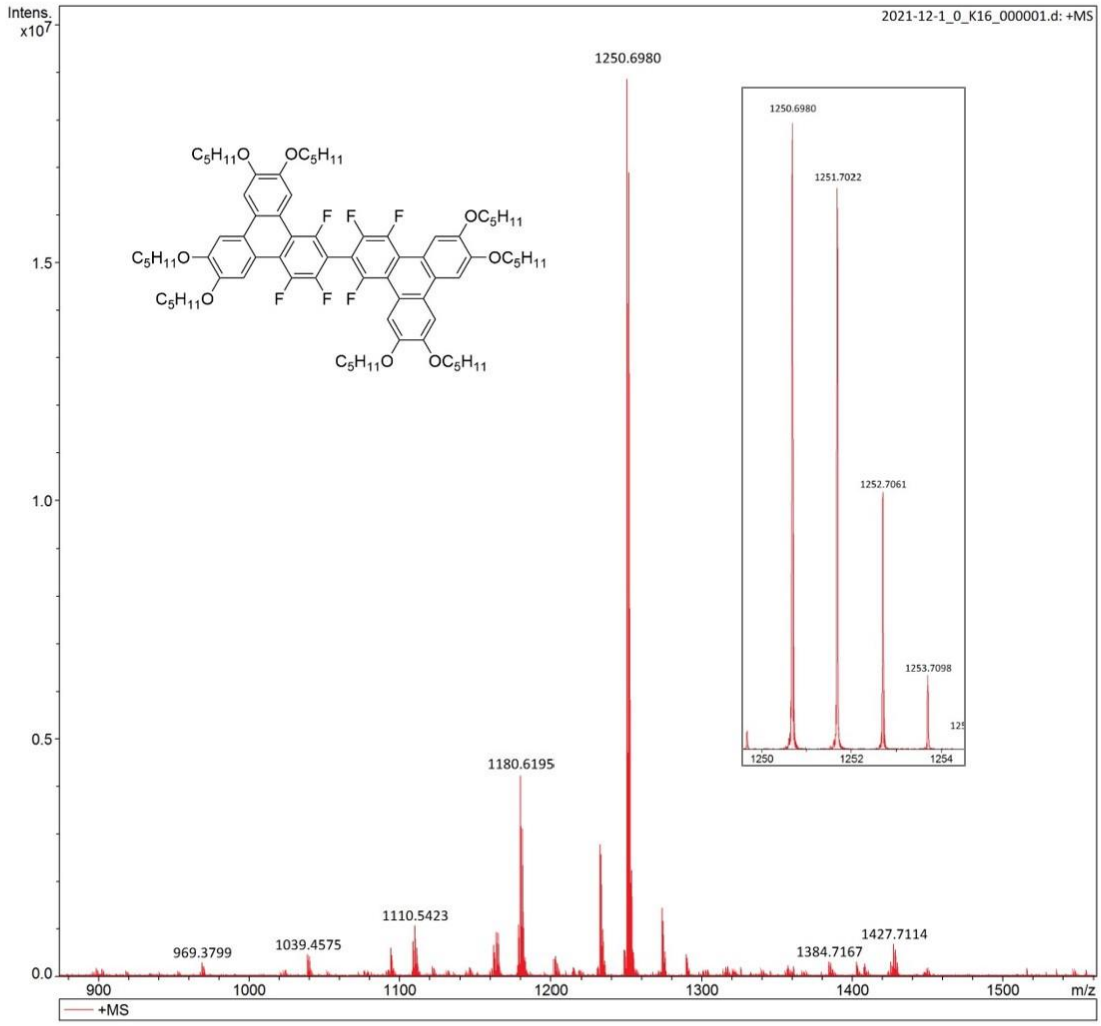

Figure S28. HRMS *m/z* (ESI) spectrum of **G**55.

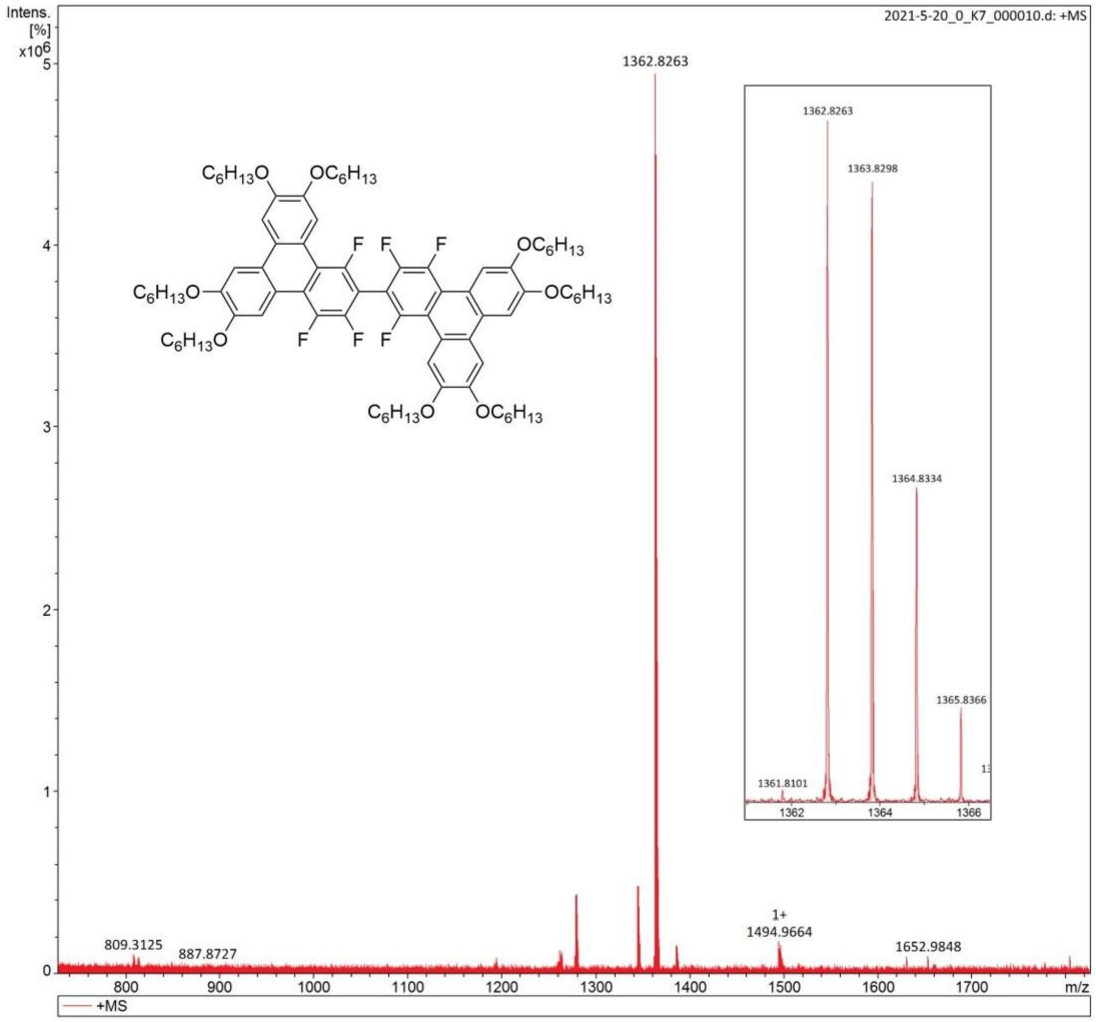

Figure S29. HRMS *m/z* (ESI) spectrum of **G**66.

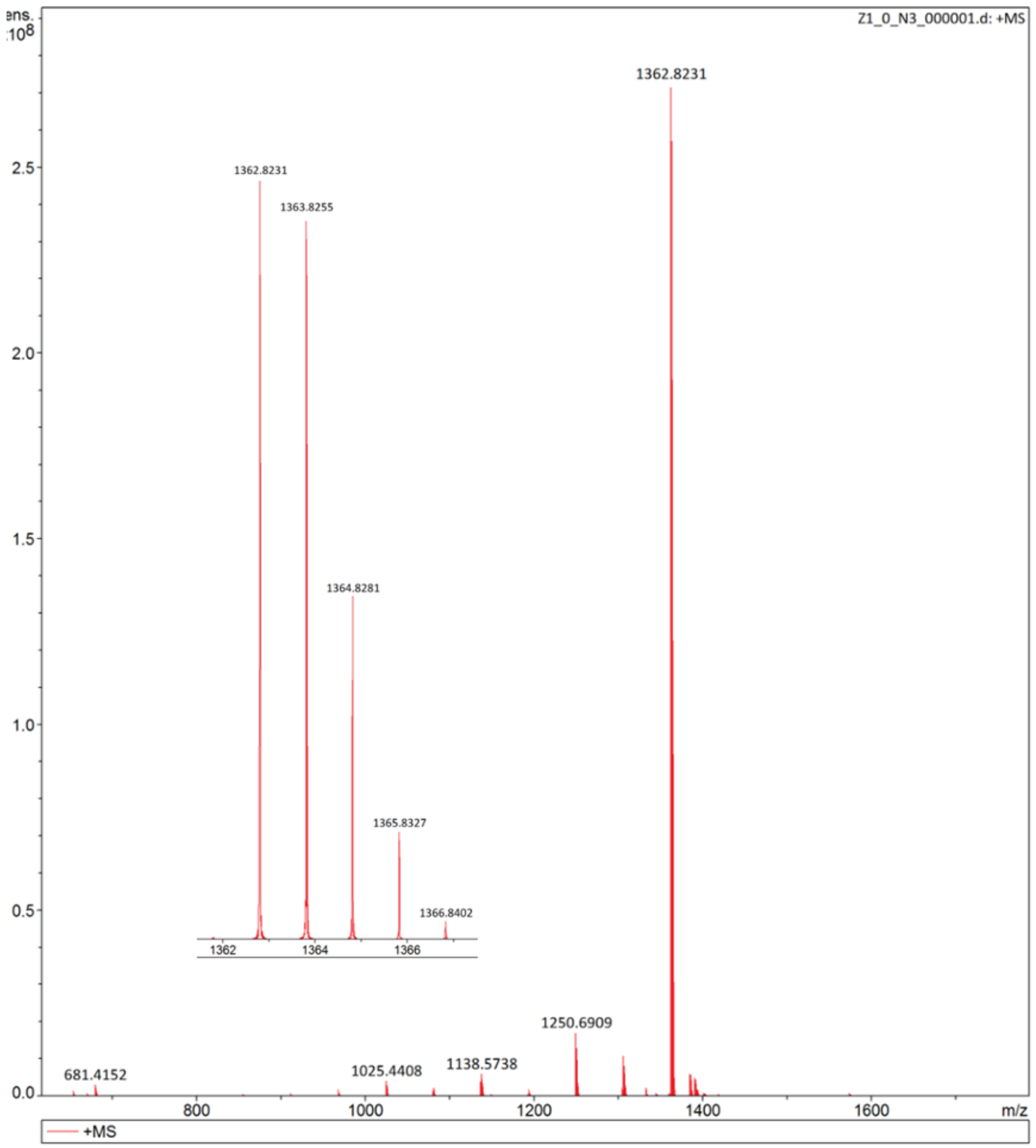

Figure S30. HRMS *m*/*z* (ESI) spectrum of **G**48.

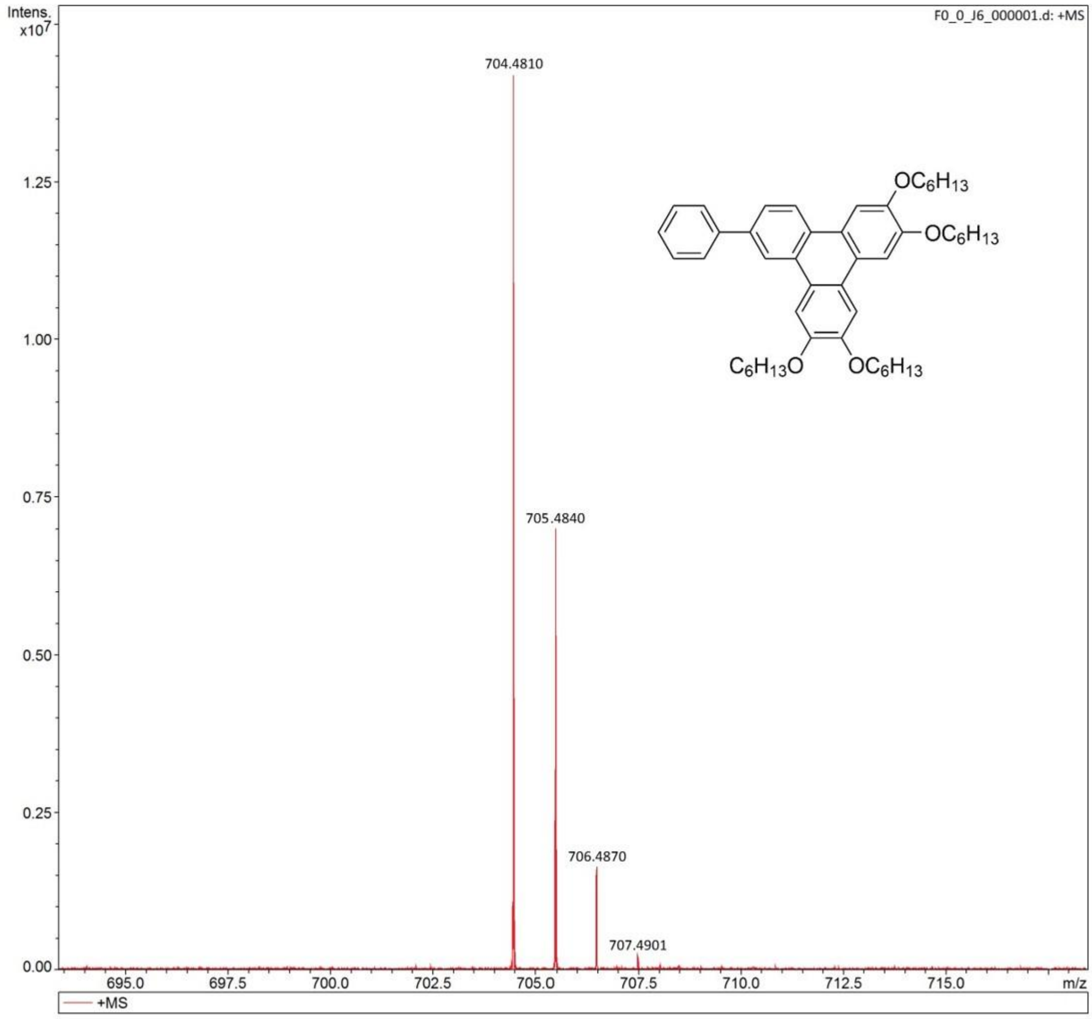

Figure S31. HRMS *m/z* (ESI) spectrum of **BTP**6.

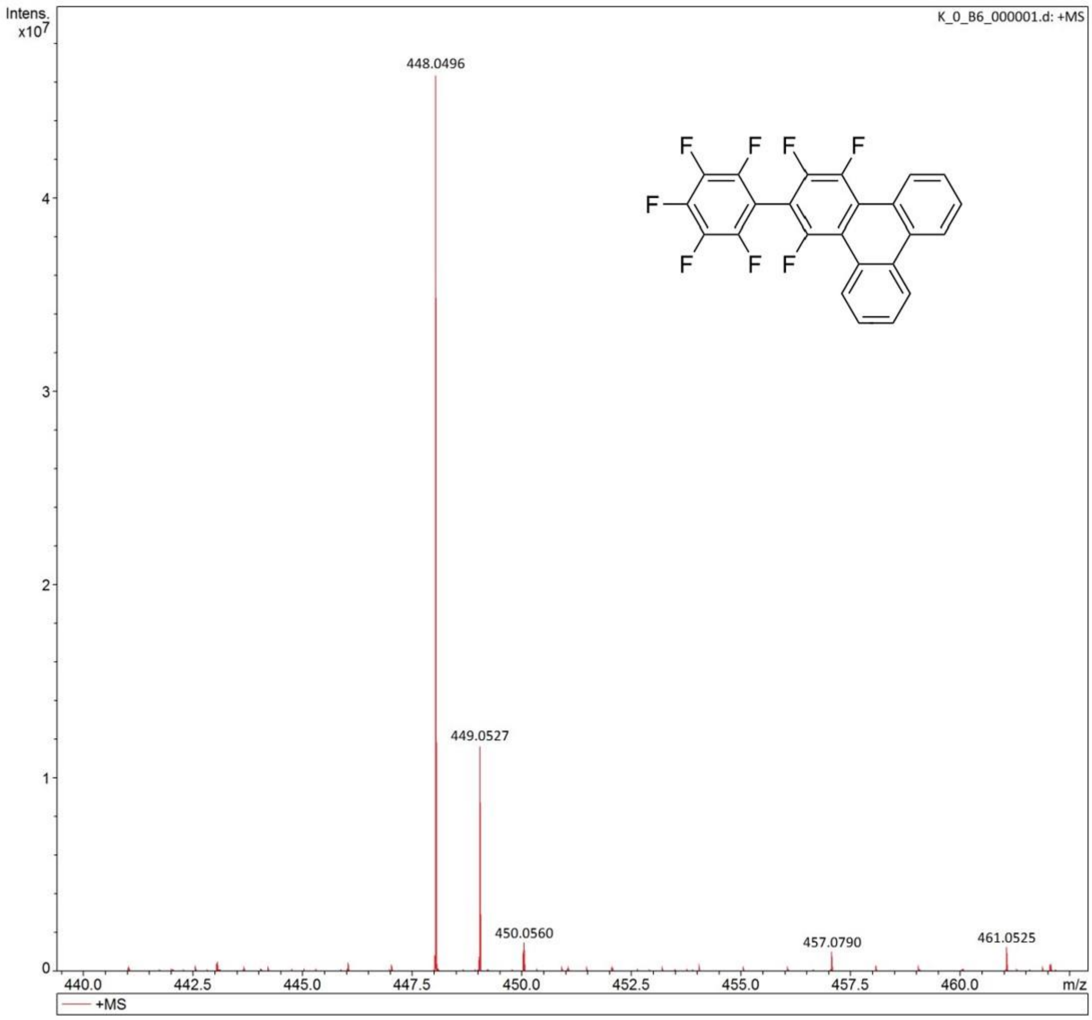

Figure S32. HRMS *m/z* (ESI) spectrum of **F**.

5. Single-crystal structural analysis

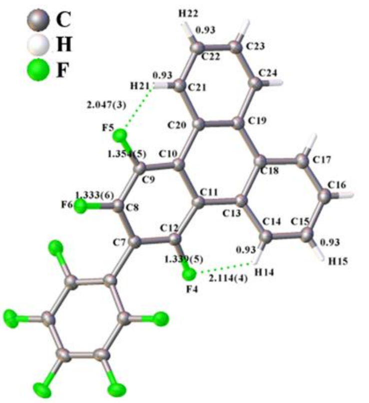

**Bond lengths** (Å)	
F5-C9	1.354(5)
C21-H21	0.93
F5-H21	2.047(3)
F4-C12	1.339(5)
C14-H14	0.93
F4-H14	2.114(4)
**Bond angles**	
C10-C9-C8	123.5(4)°
F5-C9-C8	114.3(4)°
F5-C9-C10	122.2(4)°

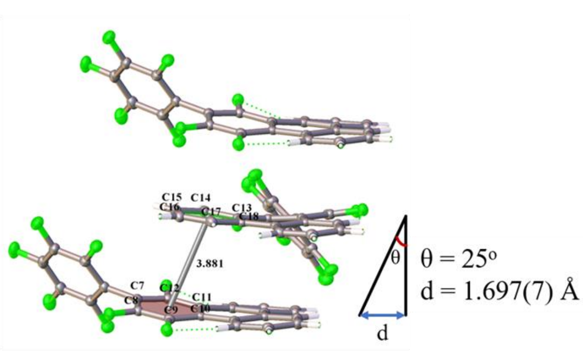

Figure S33. Single crystal structure of **F**.

View along the *a*-axis

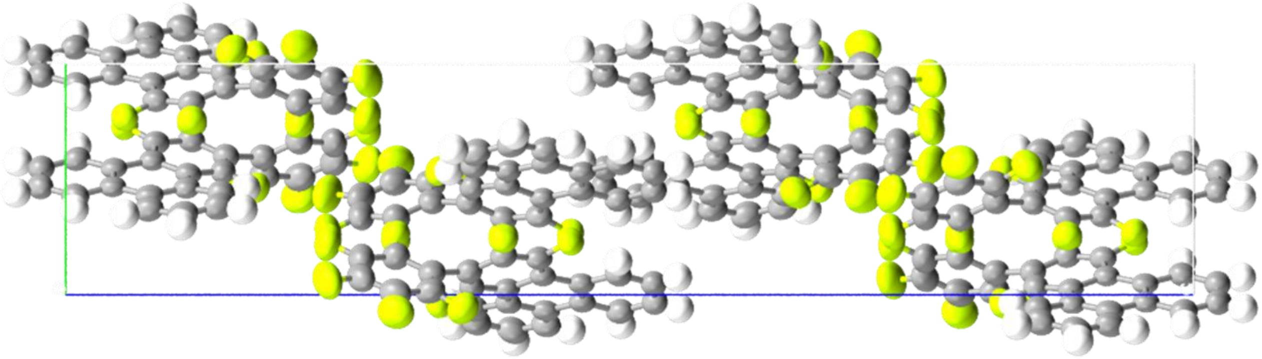

View along the *b*-axis

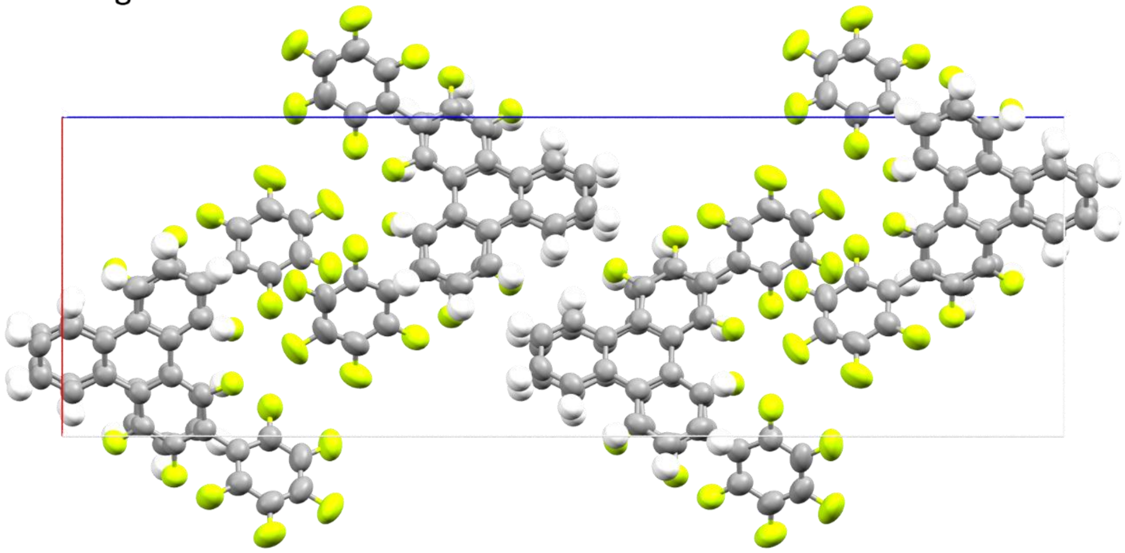

View along the *c*-axis

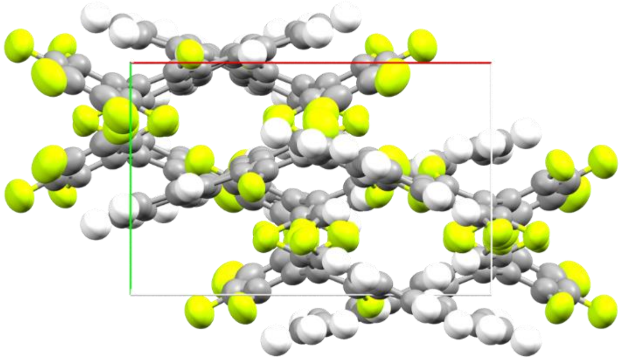

Figure S34. Single crystal structure of **F** along the axes of the lattice.

Table S1. Crystal data and structure refinement for **F**

Compound	
Empirical formula	$C_{24}H_8F_8$
Formula weight	448.30
Temperature	330.2(7)K
Crystal system	orthorhombic
Space group	*Pccn*
Unit cell dimensions	a = 12.0453(9) Å
	b = 7.7424(4) Å
	c = 37.841(2) Å
	α = 90°
	β = 90°
	γ = 90°
Volume	3529.1(4) $Å^3$
Z	8
ρ_{calc}	1.688 g/cm^3
Absorption coefficient	1.393 μ/mm^{-1}
F(000)	1792.0
Crystal size	0.18 × 0.15 × 0.15 mm^3
Radiation	Cu Kα (λ= 1.54184)
2θ rang for data collection	4.67 to 153.75°
Index ranges	-15≤h≤14, -4≤k≤9, -45≤l≤46
Reflections collected	12992
Independent reflections	3497 [R_{int} = 0.0519, R_{sigma} = 0.0348]
Data/restraints/parameters	3497/0/289
Goodness-of-fit on F^2	1.229
Final R indexes [I > = 2σ (I)]	R_1 = 0.1035, wR_2 = 0.3115
Final R indexes [all data]	R_1 = 0.1384, wR_2 = 0.3523
Largest diff. peak and hole	0.35 and -0.41 e.$Å^{-3}$
CCDC Number	2283666

Table S2. Bond lengths for **F.**

Atom	Atom	Length/Å	Atom	Atom	Length/Å
F5	C9	1.354(5)	C18	C17	1.417(8)
F4	C12	1.339(5)	C11	C10	1.437(7)
F6	C8	1.333(6)	C11	C12	1.398(7)
F7	C4	1.341(7)	C7	C12	1.383(8)
F3	C6	1.341(7)	C7	C8	1.379(7)
F2	C1	1.339(8)	C7	C5	1.474(7)
F8	C3	1.354(8)	C22	C21	1.359(7)
F1	C2	1.328(7)	C22	C23	1.358(8)

C19	C20	1.399(7)	C24	C23	1.372(8)
C19	C18	1.450(7)	C17	C16	1.352(8)
C19	C24	1.417(7)	C14	C15	1.381(8)
C20	C10	1.472(6)	C16	C15	1.382(8)
C20	C21	1.419(7)	C5	C4	1.378(8)
C13	C18	1.413(7)	C5	C6	1.403(8)
C13	C11	1.453(7)	C4	C3	1.395(8)
C13	C14	1.410(7)	C2	C1	1.370(11)
C9	C10	1.382(7)	C2	C3	1.351(10)
C9	C8	1.383(7)	C1	C6	1.379(9)

Table S3. Bond angles for **F**.

**Atom**	**Atom**	**Atom**	**Angle/˚**	**Atom**	**Atom**	**Atom**	**Angle/˚**
C20	C19	C18	120.7(4)	F6	C8	C7	120.8(4)
C20	C19	C24	118.1(5)	C7	C8	C9	120.3(5)
C24	C19	C18	121.2(5)	C23	C22	C21	120.1(5)
C19	C20	C10	119.7(4)	C22	C21	C20	122.1(5)
C19	C20	C21	117.9(4)	C23	C24	C19	121.6(5)
C21	C20	C10	122.4(4)	C22	C23	C24	120.2(5)
C18	C13	C11	119.7(4)	C16	C17	C18	122.6(5)
C14	C13	C18	117.5(5)	C15	14	C13	121.9(5)
C14	C13	C11	122.7(4)	C17	C16	C15	119.3(6)
F5	C9	C10	122.2(4)	C4	C5	C7	121.5(5)
F5	C9	C8	114.3(4)	C4	C5	C6	115.8(5)
C10	C9	C8	123.5(4)	C6	C5	C7	122.7(5)
C13	C18	C19	120.2(5)	C14	C15	C16	120.1(5)
C13	C18	C17	118.4(5)	F7	C4	C5	119.9(5)
C17	C18	C19	121.3(5)	F7	C4	C3	118.0(6)
C10	C11	C13	119.4(4)	C5	C4	C3	122.0(6)
C12	C11	C13	124.2(4)	F1	C2	C1	119.8(6)
C12	C11	C10	116.4(5)	F1	C2	C3	120.4(7)
C9	C10	C20	123.5(4)	C3	C2	C1	119.9(6)
C9	C10	C11	117.4(4)	F2	C1	C2	120.7(6)

C11	C10	C20	119.1(4)	F2	C1	C6	119.5(7)
C12	C7	C5	122.7(5)	C2	C1	C6	119.8(6)
C8	C7	C12	116.6(4)	F8	C3	C4	118.8(6)
C8	C7	C5	120.7(5)	C2	C3	F8	120.9(6)
F4	C12	C11	119.6(5)	C2	C3	C4	120.3(6)
F4	C12	C7	114.7(4)	F3	C6	C5	119.2(5)
C7	C12	C11	125.4(4)	F3	C6	C1	118.7(6)
F6	C8	C9	118.9(4)	C1	C6	C5	122.2(6)

6. TGA

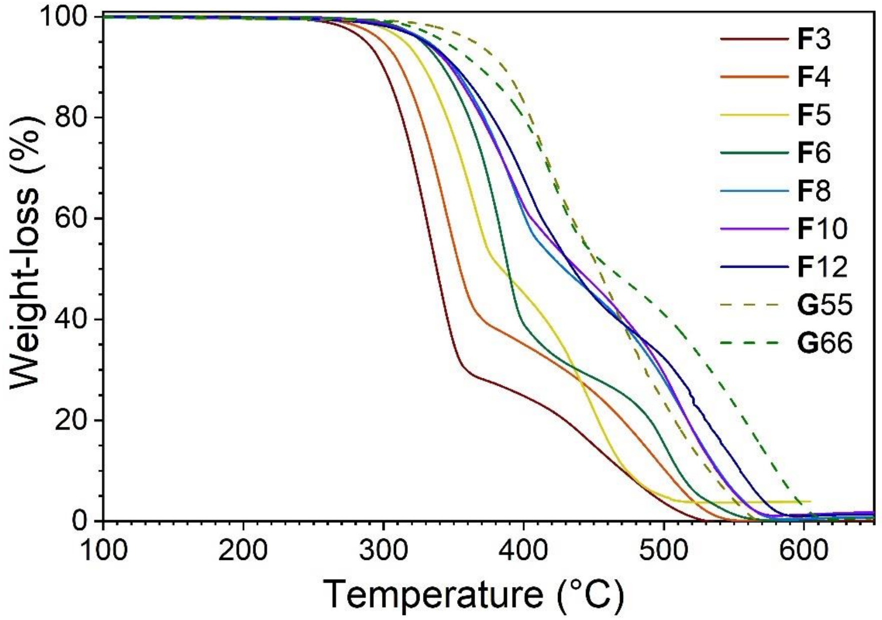

Figure S35. TGA curves of **F***n* and **G***nm*.

Table S4. Decomposition temperatures at various percentage losses of **F***n* and **G***n*.

Compounds	T_{dec}/°C (1% loss)	T_{dec}/°C (2% loss)	T_{dec}/°C (5% loss)
**F**3	257	270	283
**F**4	270	282	299
**F**5	284	297	315
**F**6	296	310	327
**F**8	282	306	332
**F**10	290	307	329
**F**12	280	303	330
**G**55	314	337	365
**G**66	304	320	344

T_{dec}. (temperature with 1%, 2%, and 5% weight-loss) were obtained by TGA measurements with a heating rate of 10 °C/min in nitrogen.

7. POM

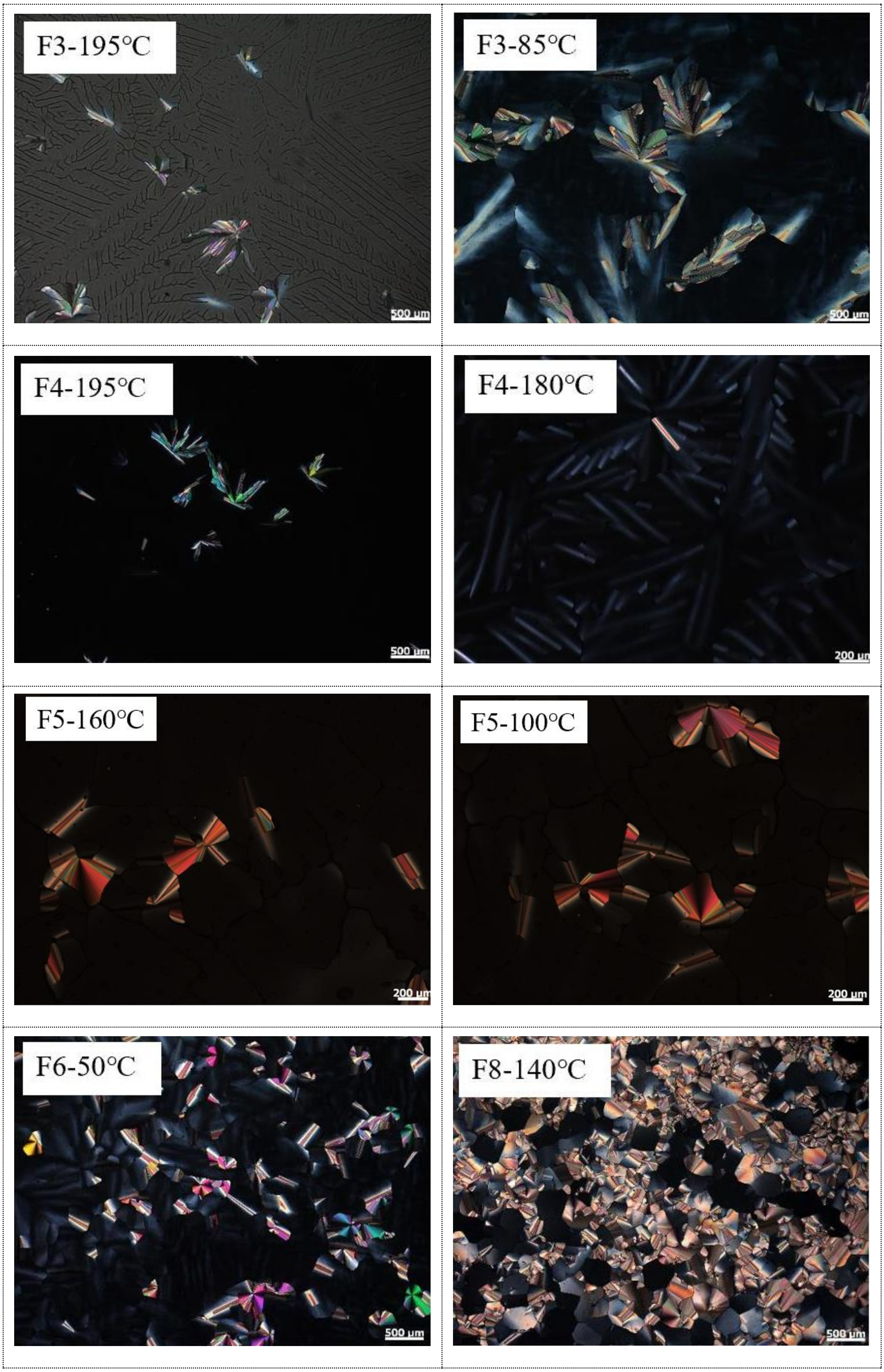

F3-195°C
500 μm
F3-85°C
500 μm
F4-195°C
500 μm
F4-180°C
200 μm
F5-160°C
200 μm
F5-100°C
200 μm
F6-50°C
500 μm
F8-140°C
500 μm

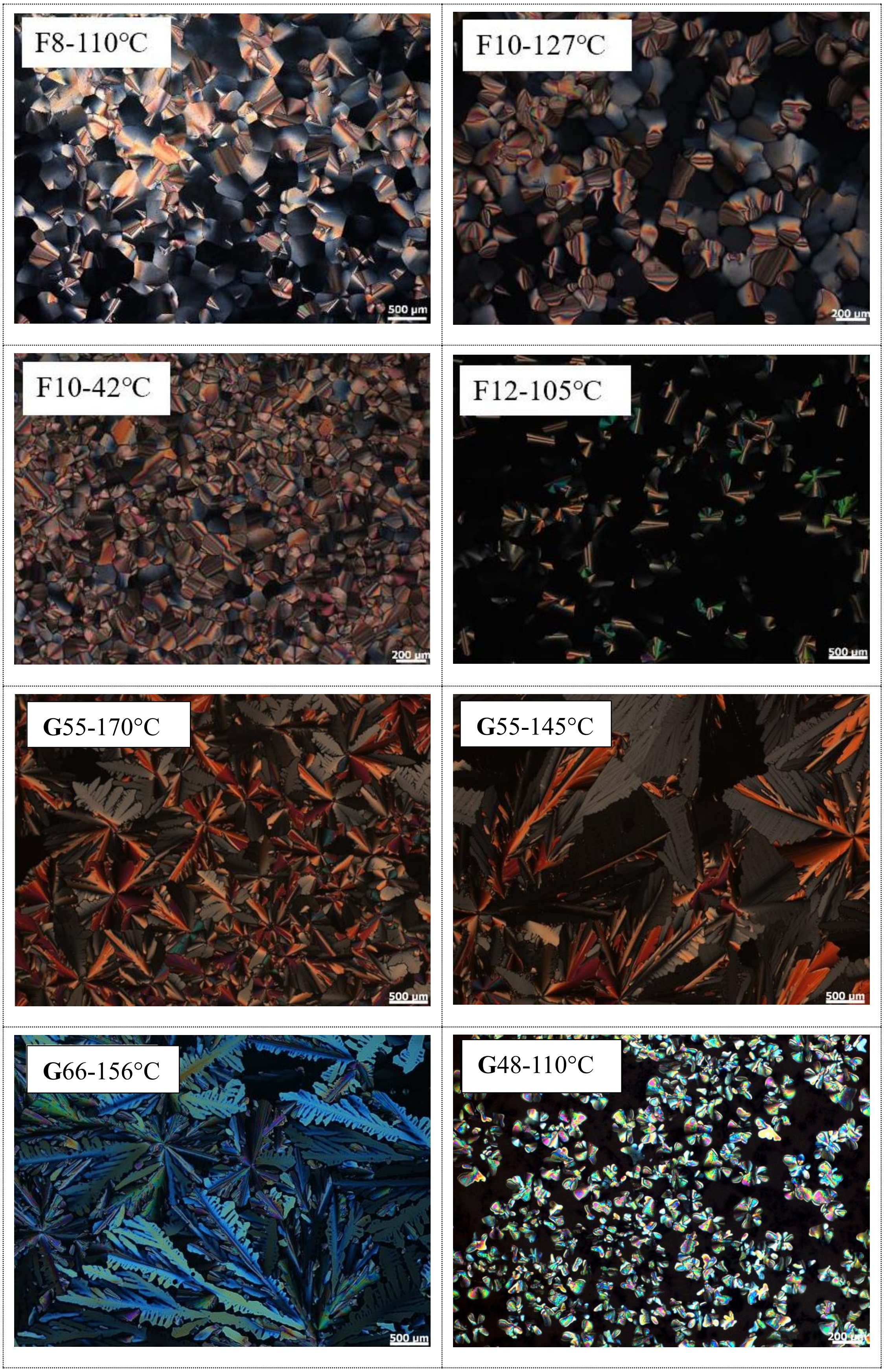

Figure S36. POM textures observed between crossed polarizers of **F***n* and **G***nm* on slowly cooling from the isotropic liquid.

8. DSC

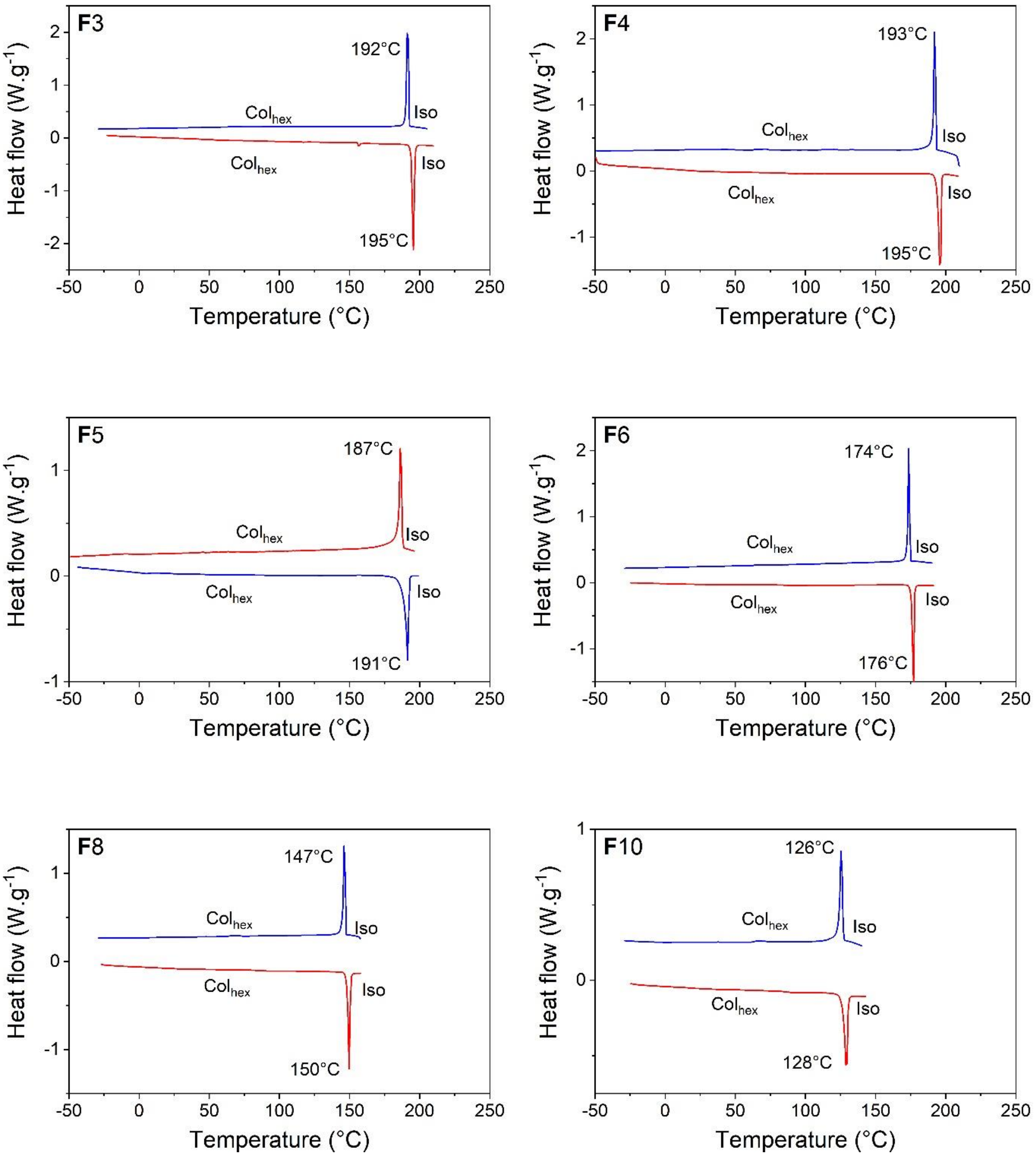
F3
192°C
195°C
Col_{hex}
Iso
F4
193°C
195°C
F5
187°C
191°C
F6
174°C
176°C
F8
147°C
150°C
F10
126°C
128°C
Heat flow (W.g^{-1})
Temperature (°C)

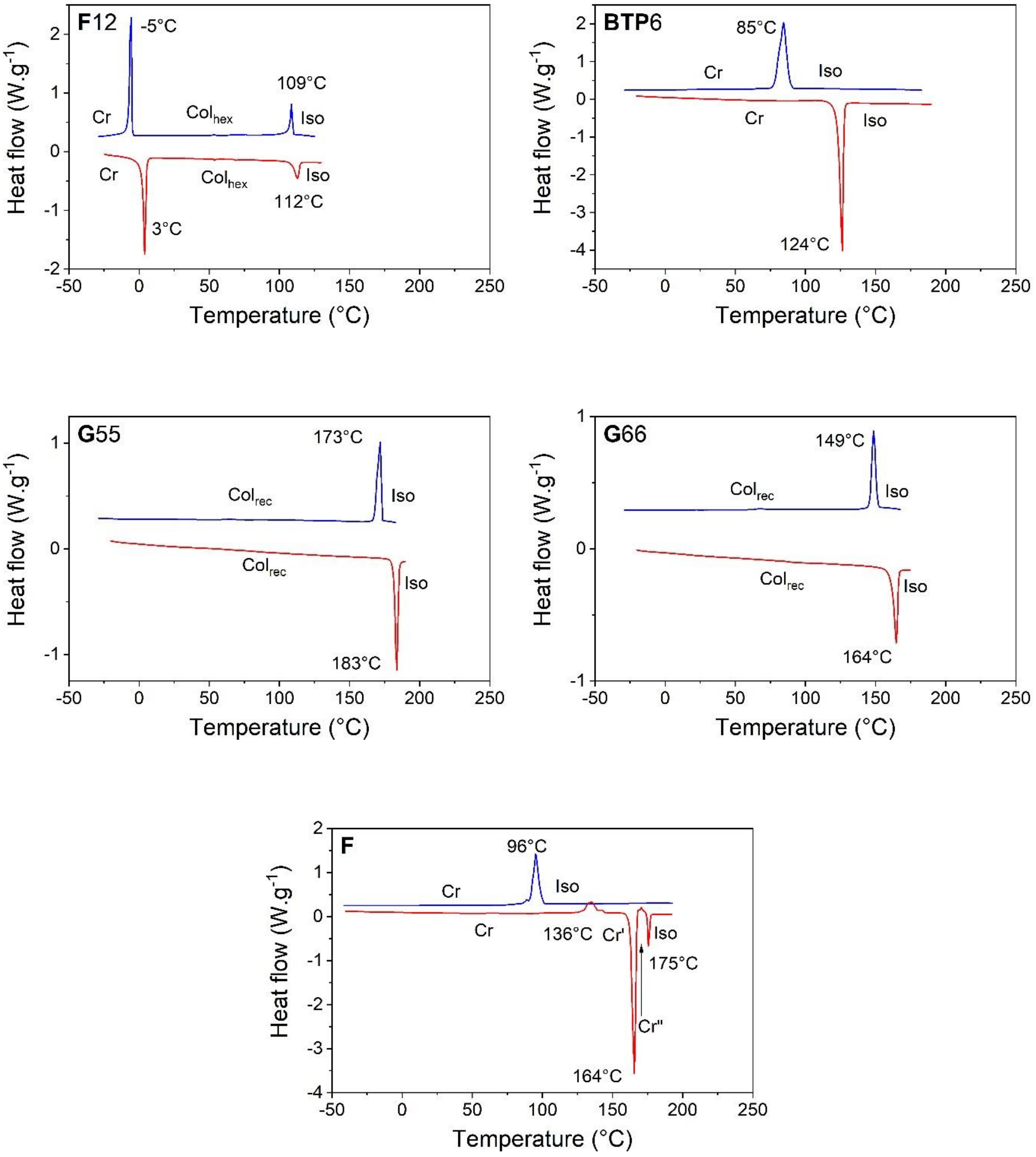

Figure S37. DSC curves of **F***n*, **F**, **BTP**6 and **G***nm* (2nd heating, red curve; 1st cooling, blue curve; rate 10 °C/min).

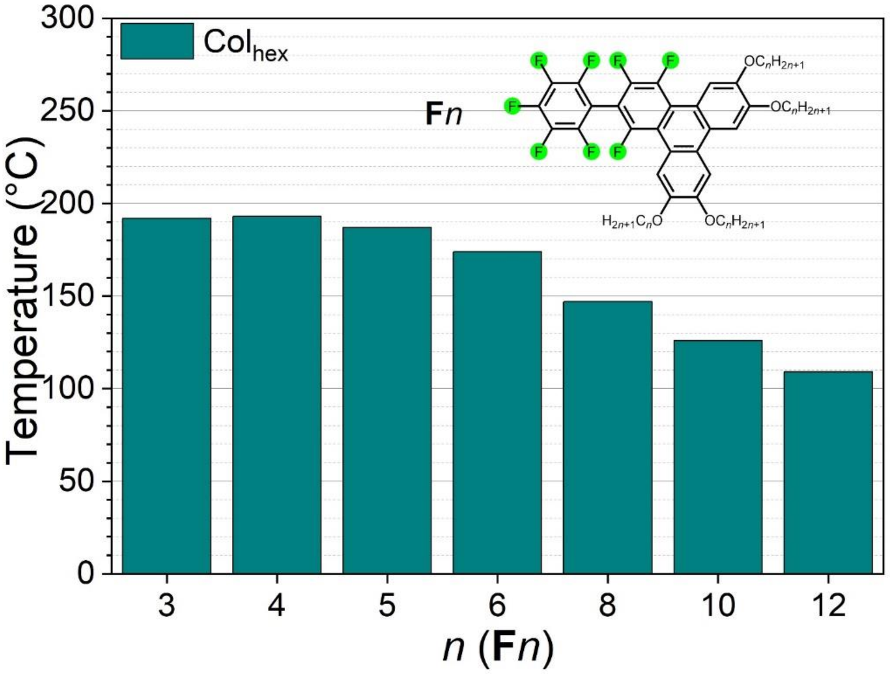

Figure S38. Bar graph of the first cooling of **F***n*.

Table S5. Mesophases, transition temperatures and enthalpy changes for **F***n*, **J** and **G***nm* (heating and cooling rate 10 °C/min).

**Cpds**	**Mesophases, transition temperature and enthalpy changes**	
	2nd heating/°C (Δ*H*, kJ/mol)	1st cooling/°C (Δ*H*, kJ/mol)
**F**3	Col$_{hex}$ 195 (14.7) I	I 192 (-14.9) Col$_{hex}$
**F**4	Col$_{hex}$ 195 (15.2) I	I 193 (-14.7) Col$_{hex}$
**F**5	Col$_{hex}$ 191 (11.8) I	I 187 (-12.8) Col$_{hex}$
**F**6	Col$_{hex}$ 176 (12.6) I	I 174 (-12.0) Col$_{hex}$
**F**8	Col$_{hex}$ 150 (12.0) I	I 147 (-11.0) Col$_{hex}$
**F**10	Col$_{hex}$ 128 (9.8) I	I 126 (-9.5) Col$_{hex}$
**F**12	Cr 3 (29.8) Col$_{hex}$ 112 (8.2) I	I 109 (-8.2) Col$_{hex}$ -5 (-28.5) Cr
**F**	Cr 136 (-) Cr’ 164 (-) Cr” 175 I	I 96 () Cr
**BTP**6	Cr 124 (52.8) I	I 85 (-44.4) Cr
**G**55	Col$_{rec}$ 183 (19.3) I	I 173 (-18.4) Col$_{rec}$
**G**66	Col$_{rec}$ 164 (16.3) I	I 149 (-16.1) Col$_{rec}$
**G**48*	Colrec 120 I	-

Abbreviations: Cr: crystalline phases; Col$_{hex}$: columnar hexagonal mesophase; Col$_{rec}$: columnar rectangular phase; I: isotropic liquid. * Determined by POM only.

9. S/WAXS

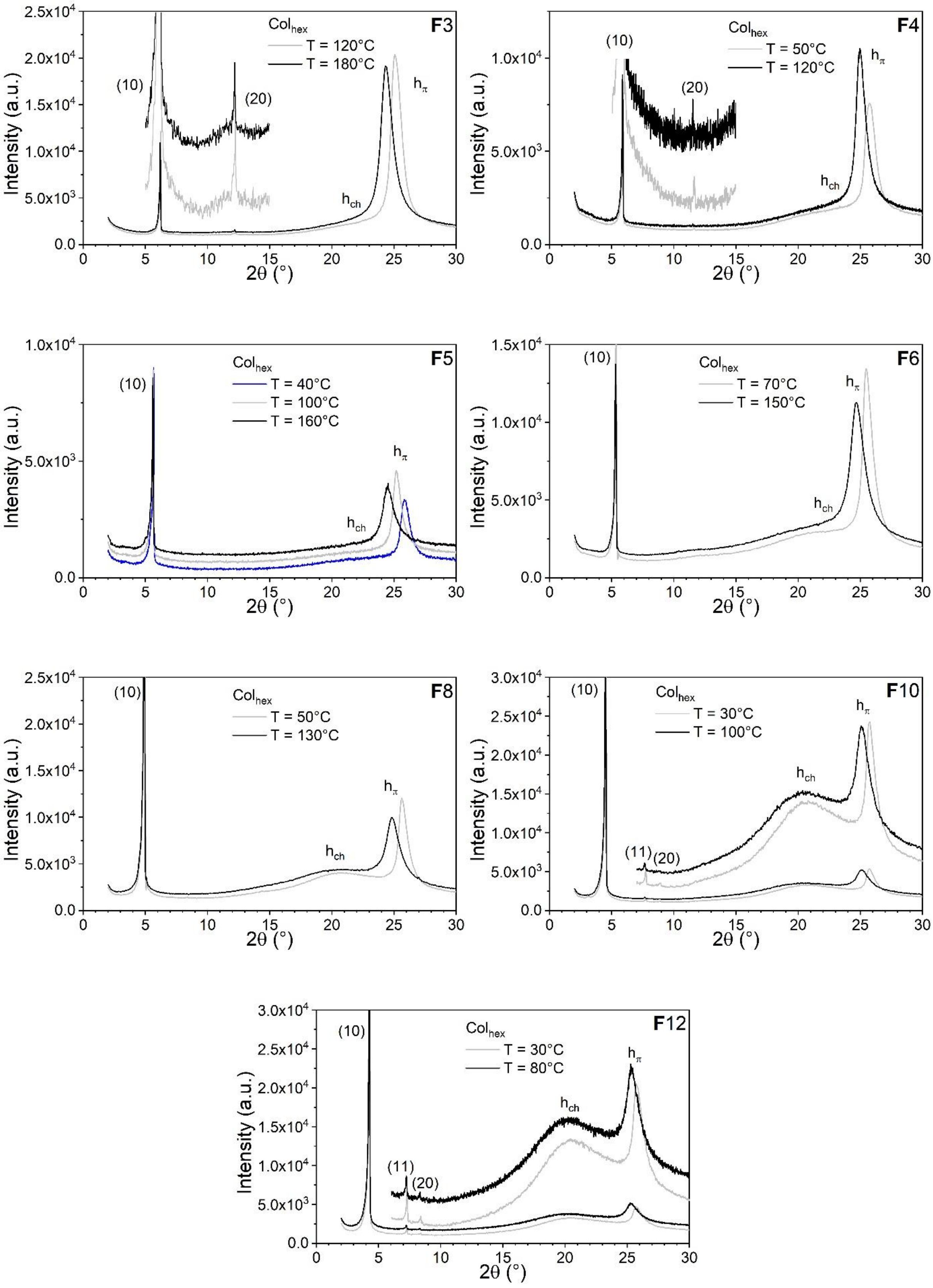

Figure S39. S/WAXS patterns of the mesophases of **F***n* (recorded on cooling).

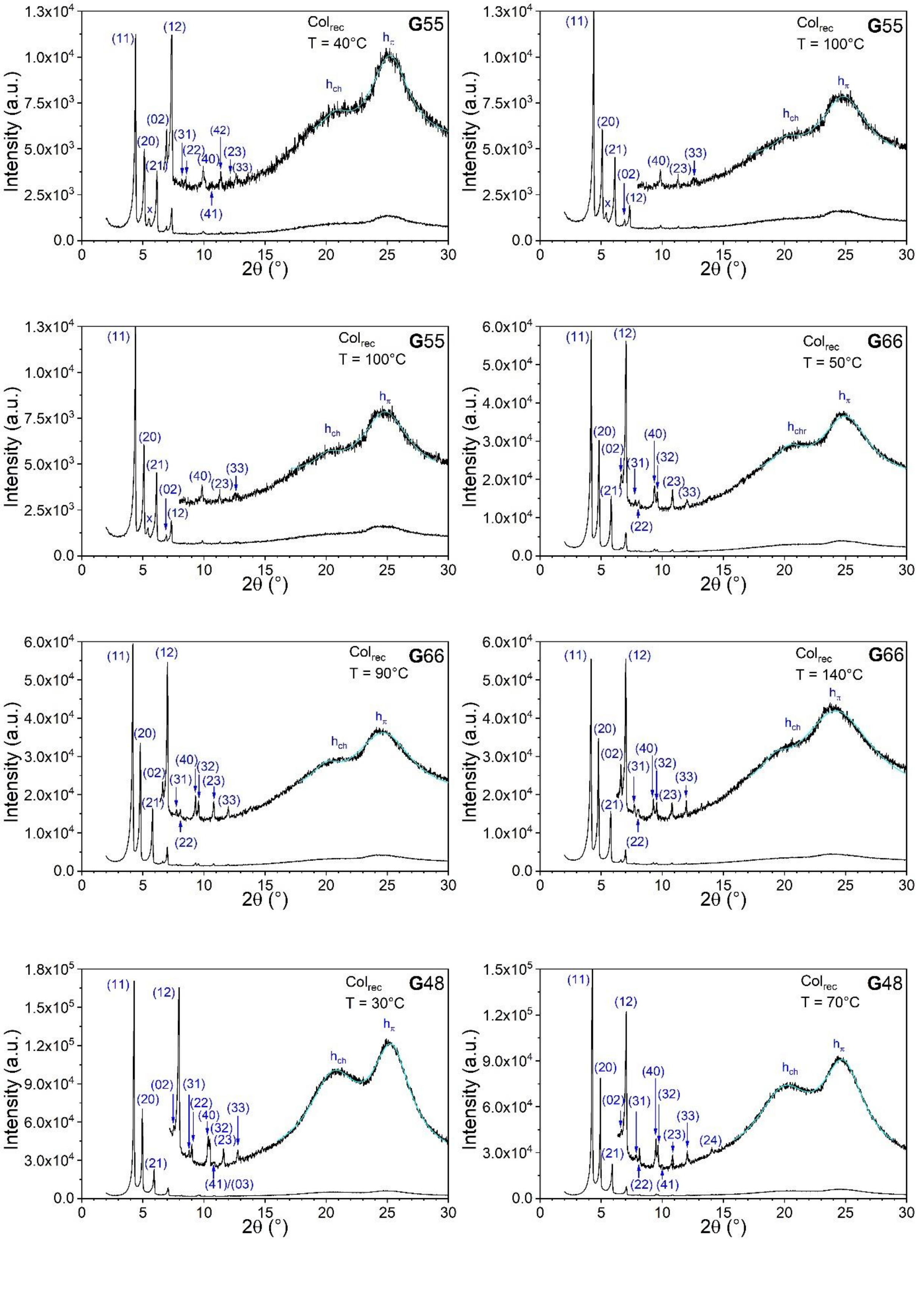

Col$_{rec}$
G55
T = 40°C
(11)
(12)
(02)
(20)
(21)
x
(31)
(22)
(40)
(42)
(23)
(33)
(41)
h$_{ch}$
h$_{\pi}$
Col$_{rec}$
G55
T = 100°C
Col$_{rec}$
G66
T = 50°C
h$_{chr}$
(32)
Col$_{rec}$
G66
T = 90°C
Col$_{rec}$
G66
T = 140°C
Col$_{rec}$
G48
T = 30°C
(41)/(03)
Col$_{rec}$
G48
T = 70°C
(24)
Intensity (a.u.)
2θ (°)

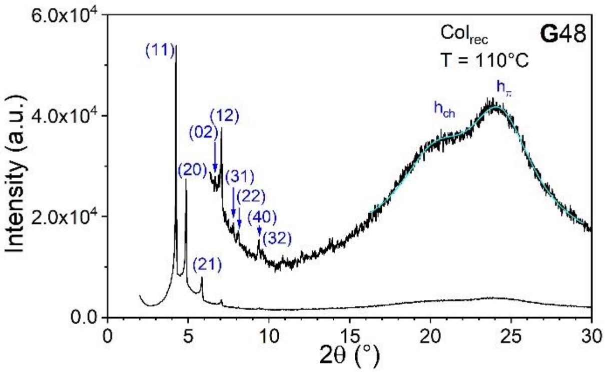

Figure S40. S/WAXS patterns of the mesophases of **G***n* (recorded on cooling).

Table S6. Indexation of the SAXS patterns of compounds **F***n* and **G***n*.

Compd	$2\theta_{exp}$	d_{obs} (Å)	I (%)	hk	d_{calc} (Å)	Lattice parameters
**F3** (120 °C)	6.099<br>12.198<br>20.11<br>25.17	14.48<br>7.25<br>4.41<br>3.53	VS<br>VW<br>M<br>VS	10<br>20<br>h_{ch}<br>h_π (67)	14.49<br>7.25<br>-<br>-	Col$_{hex}$<br>a = 16.73 Å<br>A = 242.44 Å^2
**F3** (180 °C)	6.109<br>12.19<br>20.16<br>24.40	14.45<br>7.25<br>4.40<br>3.64	VS<br>VW<br>M<br>VS	10<br>20<br>h_{ch}<br>h_π (56)	14.47<br>7.24<br>-<br>-	Col$_{hex}$<br>a = 16.71 Å<br>A = 241.77 Å^2
**F4** (50 °C)	5.804<br>11.62<br>20.06<br>25.79	15.21<br>7.61<br>4.42<br>3.45	VS<br>VW<br>M<br>VS	10<br>20<br>h_{ch}<br>h_π (64)	15.22<br>7.61<br>-<br>-	Col$_{hex}$<br>a = 17.57 Å<br>A = 267.38 Å^2
**F4** (120 °C)	5.731<br>11.51<br>20.60<br>25.03	15.41<br>7.66<br>4.31<br>3.55	VS<br>VW<br>M<br>VS	10<br>20<br>h_{ch}<br>h_π (65)	15.37<br>7.68<br>-<br>-	Col$_{hex}$<br>a = 17.74 Å<br>A = 272.60 Å^2
**F5** (40 °C)	5.523<br>19.96<br>25.90	15.99<br>4.44<br>3.44	VS<br>W<br>VS	10<br>h_{ch}<br>h_π (79)	15.99<br>-<br>-	Col$_{hex}$<br>a = 18.46 Å<br>A = 295.14 Å^2
**F5** (100 °C)	5.505<br>20.25<br>25.24	16.04<br>4.38<br>3.52	VS<br>W<br>VS	10<br>h_{ch}<br>h_π (83)	16.04<br>-<br>-	Col$_{hex}$<br>a = 18.52 Å<br>A = 297.08 Å^2
**F5** (160 °C)	5.491<br>20.32<br>24.54	16.08<br>4.37<br>3.62	VS<br>W<br>VS	10<br>h_{ch}<br>h_π (59)	16.08<br>-<br>-	Col$_{hex}$<br>a = 18.57 Å<br>A = 298.57 Å^2
**F6** (70 °C)	5.281<br>19.84<br>25.56	16.72<br>4.47<br>3.48	VS<br>W<br>VS	10<br>h_{ch}<br>h_π (72)	16.72<br>-<br>-	Col$_{hex}$<br>a = 19.31 Å<br>A = 322.78 Å^2

**F**6 (150 °C)	5.213	16.94	VS	10	16.94	Col_{hex}
	19.34	4.58	W	h_{ch}	-	a = 19.56 Å
	24.74	3.60	VS	h_{π} (47)	-	A = 331.25 Å^2
**F**8 (50 °C)	4.758	18.56	VS	10	18.56	Col_{hex}
	20.63	4.30	M	h_{ch}	-	a = 21.43 Å
	25.70	3.46	VS	h_{π} (79)	-	A = 397.59 Å^2
**F**8 (130 °C)	4.751	18.58	VS	10	18.58	Col_{hex}
	20.14	4.40	M	h_{ch}	-	a = 21.46 Å
	24.85	3.58	VS	h_{π} (42)	-	A = 398.76 Å^2
**F**10 (30 °C)	4.472	19.74	VS	10	19.74	
	7.749	11.40	W	11	11.40	Col_{hex}
	8.952	9.87	VW	20	9.87	a = 22.79 Å
	20.32	4.37	S	h_{ch}	-	A = 450.03 Å^2
	25.78	3.45	VS	h_{π} (78)	-	
**F**10 (100 °C)	4.415	20.00	VS	10	20.00	Col_{hex}
	7.645	11.55	W	11	11.55	a = 23.09 Å
	20.35	4.36	S	h_{ch}	-	A = 461.88 Å^2
	25.11	3.54	VS	h_{π} (42)	-	
**F**12 (30 °C)	4.194	21.05	VS	10	21.04	
	7.268	12.15	W	11	12.15	Col_{hex}
	8.394	10.52	W	20	10.52	a = 24.30 Å
	20.25	4.38	S	h_{ch}	-	A = 511.40 Å^2
	25.77	3.45	VS	h_{π} (75)	-	
**F**12 (80 °C)	4.166	21.19	VS	10	21.19	
	7.222	12.23	W	11	12.23	Col_{hex}
	8.338	10.60	W	20	10.59	a = 24.47 Å
	20.14	4.40	S	h_{ch}	-	A = 518.53 Å^2
	25.32	3.51	VS	h_{π} (47)	-	
**G**55 (40 °C)	4.279	20.63	VS	11	20.63	Col_{rec}
	5.017	17.60	VS	20	17.60	a = 35.20 Å
	5.56	15.88	VW	xx	xx	b = 25.46 Å
	6.088	14.50	VS	21	14.48	A = 896.19 Å^2
	6.911	12.78	M	02	12.73	
	7.349	12.02	VS	12	11.97	
	8.25	10.71	VW	31	10.66	
	8.56	10.32	VW	22	10.31	
	10.05	8.79	W	40	8.80	
	10.66	8.29	VW	41	8.32	
	11.46	7.71	W	42	7.64	
	12.15	7.28	VW	23	7.28	
	12.67	6.98	VW	33	6.88	
	20.71	4.28	S	h_{ch}	-	
	25.21	3.53	S	h_{π}	-	

**G**55 (100 ^oC)	4.266	20.69	VS	11	20.69	Col_{rec} a = 35.26 Å b = 25.55 Å A = 900.89 $Å^2$
	5.008	17.63	VS	20	17.63	
	5.41	16.32	VW	xx	xx	
	6.083	14.52	VS	21	14.51	
	6.91	12.78	VW	02	12.77	
	7.33	12.05	M	12	12.01	
	9.92	8.91	W	40	8.82	
	11.37	7.77	W	23	7.69	
	12.67	6.98	W	33	6.90	
	19.93	4.45	S	h_{ch}	-	
	24.18	3.68	S	h_{π}	-	
**G**55 (160 ^oC)	4.244	20.80	VS	11	20.80	Col_{rec} a = 35.32 Å b = 25.74 Å A = 909.00 $Å^2$
	4.999	17.66	VS	20	17.66	
	5.300	16.66	VW	xx	xx	
	6.083	14.52	VS	21	14.56	
	6.838	12.91	VW	02	12.87	
	7.33	12.05	M	12	12.09	
	8.19	10.79	VW	31	10.71	
	8.47	10.43	VW	22	10.40	
	9.90	8.93	V	40	8.83	
	11.31	7.82	VW	23	7.72	
	12.66	6.99	VW	33	6.94	
	19.68	4.51	S	h_{ch}	--	
	24.35	3.65	S	h_{π}	--	
**G**48 (30 ^oC)	4.095	21.56	VS	11	21.56	Col_{rec} a = 36.78 Å b = 26.61 Å A = 978.71 $Å^2$
	4.801	18.39	VS	20	18.39	
	5.875	15.03	M	21	15.13	
	6.65	13.28	VW	02	13.30	
	7.077	12.48	M	12	12.51	
	7.94	11.12	VW	31	11.13	
	8.17	10.81	VW	22	10.78	
	9.55	9.25	W	40	9.19	
	9.71	9.09	W	32	9.02	
	10.07	8.78	VW	41/03	8.67/8.87	
	10.83	8.16	VW	23	7.99	
	12.09	7.32	VW	33	7.19	
	20.19	4.39	S	h_{ch}		
	25.15	3.54	S	h_{π}		

**G**48 (70 °C)	4.095	21.56	VS	11	21.56	Col_{rec} a = 36.98 Å b = 26.54 Å A = 981.45 $Å^2$
	4.774	18.50	VS	20	18.50	
	5.830	15.15	M	21	15.17	
	6.66	13.27	VW	02	13.27	
	7.035	12.55	M	12	12.49	
	7.90	11.18	VW	31	11.18	
	8.18	10.80	VW	22	10.78	
	9.52	9.28	W	40	9.24	
	9.70	9.11	W	32	9.03	
	10.06	8.78	VW	41	8.73	
	10.94	8.07	VW	23	7.98	
	12.11	7.30	VW	33	7.19	
	14.12	6.27	VW	24	6.24	
	19.92	4.45	S	h_{ch}	-	
	24.80	3.59	S	h_{π}	-	
**G**48 (110 °C)	4.112	21.47	VS	11	-	Col_{rec} a = 37.14 Å b = 26.31 Å A = 977.15 $Å^2$
	4.754	18.57	VS	20	-	
	5.818	15.18	S	21	15.17	
	6.71	13.17	VW	02	13.15	
	7.115	12.42	M	12	12.40	
	7.81	11.32	VW	31	11.20	
	8.15	10.84	VW	22	10.73	
	9.43	9.37	VW	40	9.28	
	9.70	9.11	VW	32	9.01	
	20.21	4.39	S	h_{ch}	-	
	24.30	3.66	S	h_{π}	-	
**G**66 (50 °C)	4.061	21.74	VS	11	21.74	Col_{rec} a = 37.18 Å b = 26.80 Å A = 996.42 $Å^2$
	4.749	18.59	VS	20	18.59	
	5.780	15.28	S	21	15.27	
	6.62	13.34	W	02	13.40	
	7.021	12.58	M	12	12.61	
	7.83	11.28	VW	31	11.25	
	8.10	10.90	VW	22	10.87	
	9.41	9.39	W	40	9.30	
	9.63	9.18	W	32	9.10	
	10.88	8.12	W	23	8.05	
	12.10	7.31	W	33	7.25	
	20.36	4.36	S	h_{ch}	-	
	24.97	3.56	S	h_{π}	-	

**G**66 (90 °C)	4.070	21.69	VS	11	21.69	Col_{rec} a = 37.32 Å b = 26.65 Å A = 994.58 Å^2
	4.73	18.66	VS	20	18.66	
	5.795	15.24	S	21	15.28	
	6.66	13.26	W	02	13.32	
	7.043	12.54	M	12	12.55	
	7.79	11.34	VW	31	11.27	
	8.09	10.91	VW	22	10.84	
	9.35	9.45	W	40	9.33	
	9.60	9.20	W	32	9.09	
	10.85	8.15	W	23	8.02	
	12.04	7.34	W	33	7.23	
	19.80	4.48	S	h_{ch}	-	
	24.32	3.66	S	h_{π}	-	
**G**66 (140 °C)	4.078	21.65	VS	11	21.65	Col_{rec} a = 37.46 Å b = 26.53 Å A = 993.81 Å^2
	4.713	18.73	VS	20	18.73	
	5.775	15.29	S	21	15.30	
	6.64	13.30	W	02	13.26	
	7.033	12.56	M	12	12.50	
	7.75	11.40	VW	31	11.30	
	8.08	10.93	VW	22	10.82	
	9.32	9.48	W	40	9.36	
	9.58	9.22	W	32	9.09	
	10.84	8.15	W	23	8.00	
	12.03	7.35	W	33	7.22	
	19.70	4.50	S	h_{ch}	-	
	23.74	3.75	S	h_{π}	-	

Indexation: $2\theta_{exp}$: measured diffraction angle (peak position); d_{exp} and d_{cal}: experimental and calculated spacings; I: relative intensity of diffracted peak in %; *hk*: Miller indices; h_{ch}: average distance (Å) between alkyl chains; h_{π}: average distance (Å) of π–π stacking of molecules; ξ: correlation length (Å), determined by Debye–Scherrer formula.

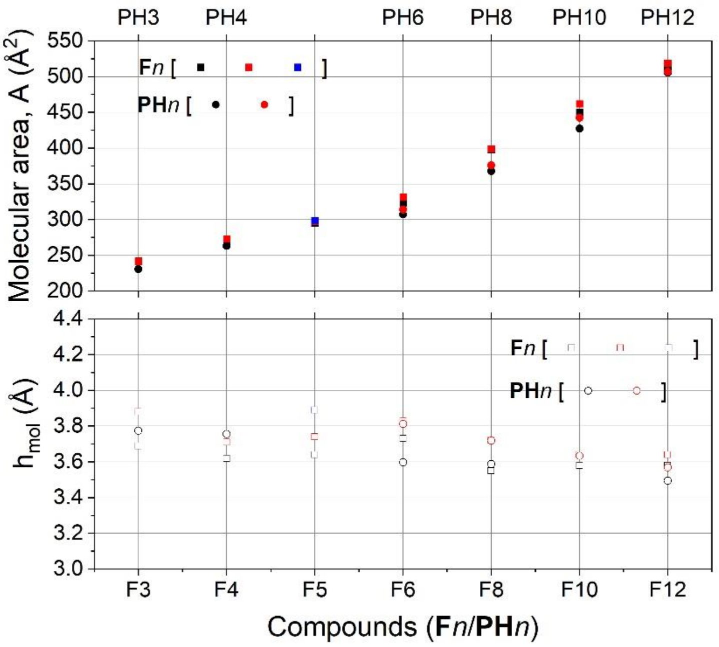

Figure S41. Variations of the cross-sectional area and h_{mol} of **F***n*, compared with **PH***n* compounds, at different temperatures.

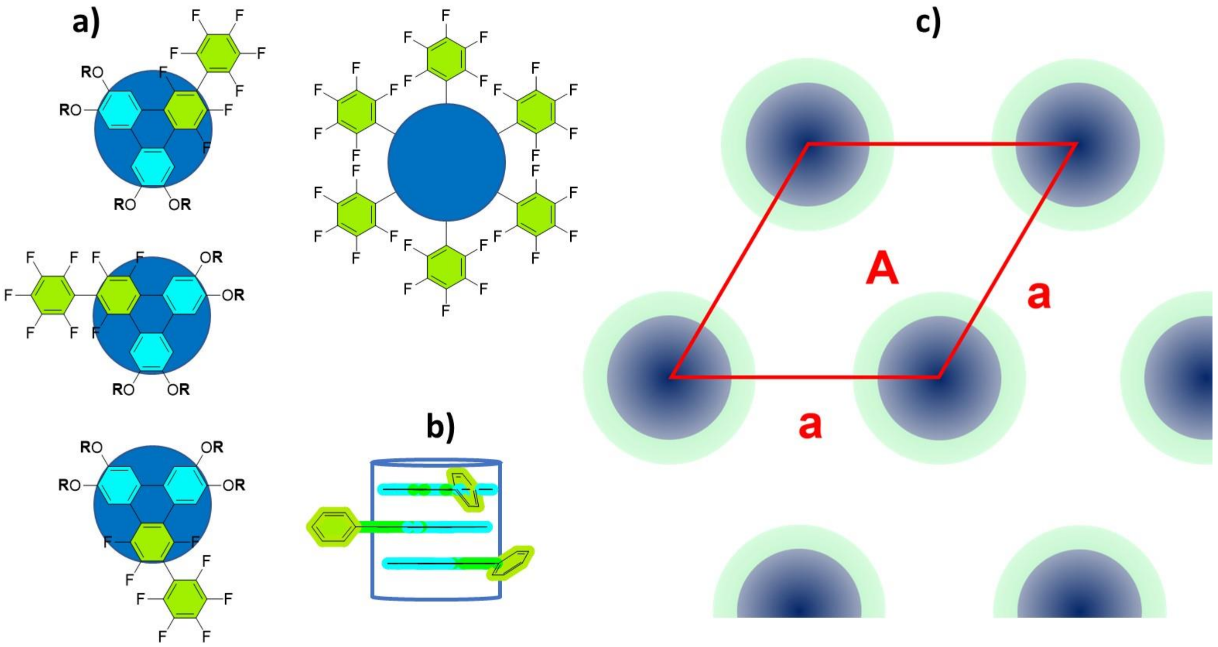

Figure S42. Schematic representation of the supramolecular organization of **F***n* compounds in the Col_{hex} phase; a) different orientations of the compounds and top view of the average molecular stacking; b) side view of the stacking of the triphenylene segments (in blue circle) with maximized arene-perfluoroarene intermolecular interactions; c) hexagonal lattice with diffuse aliphatic-fluorinated and aromatic-fluorinated interfaces; aliphatic chains not shown.

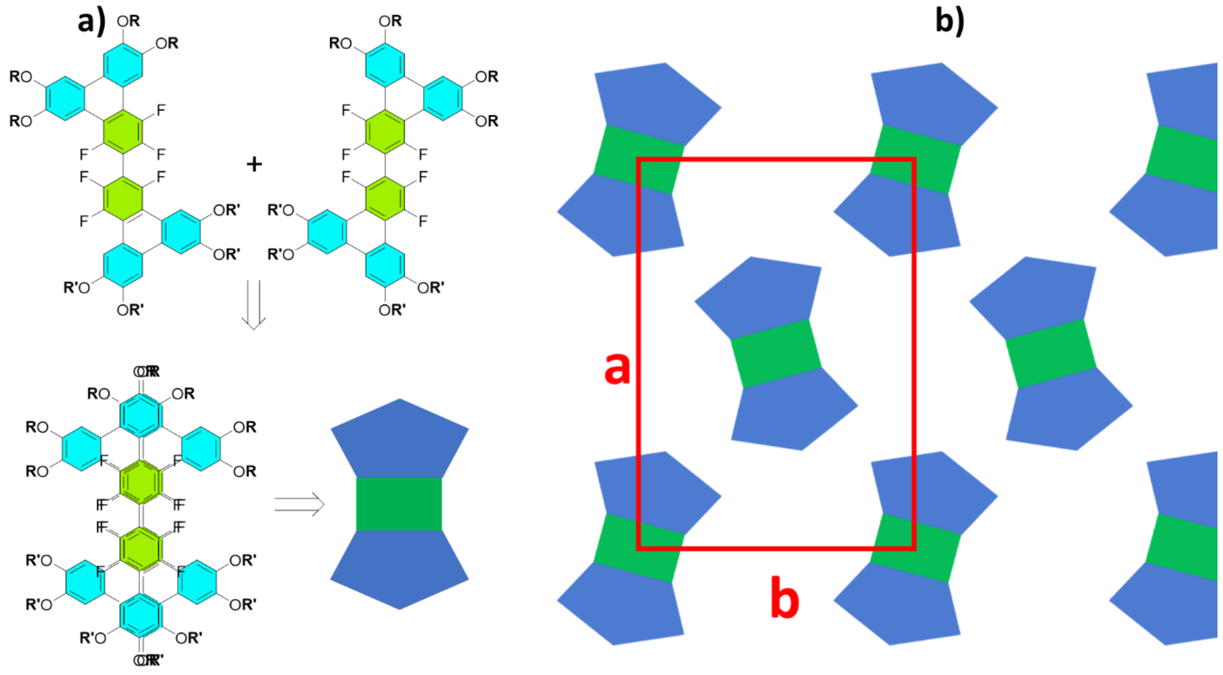

Figure S43. Schematic representation of the supramolecular organization of **G***nm* compounds in the Col_{rec} phase; a) top view of the alternated average molecular stacking; b) rectangular *p*2*gg* lattice with well segregated aromatic, fluorinated and aliphatic zones; aliphatic chains not shown.

10. Photophysical properties

Table S7. Spectroscopic parameters of **F**6 and **G**6 in solution and thin-films.

Compound	Solvents	λ_{abs} (nm)	ε (× 10^4, L·mol^{-1}·cm^{-1})	λ_{em} (nm) solution	λ_{em} (nm) film	QY [%] solution
	CHX	380	4.50	408		30.28
	TOL	380	6.22	420		24.00
**F**6	DCM	378	7.03	425	450	25.46
	TCM	380	6.86	417		22.56
	THF	380	6.39	427		27.03
	CHX	378	9.99	472		29.20
	TOL	380	11.34	488		32.57
**G**66	DCM	378	7.15	497	610	29.91
	TCM	378	10.50	489		27.52
	THF	380	12.74	502		28.53

11. DFT

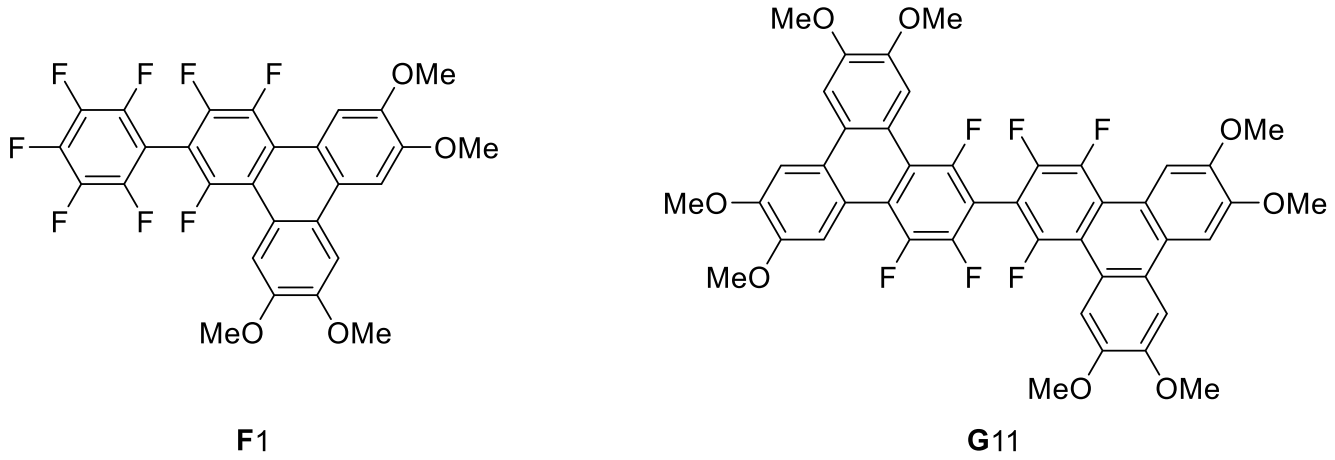

Figure S44. Molecular structure of **F**1 and **G**11

Table S8. DFT calculated FMO energy levels for **F**1 and **G**1[1]

	**F**1 (eV)	**G**11 (eV)
**HOMO**	-5.80	-5.60
**LUMO**	-1.88	-1.78
**HOMO-1**	-6.14	-5.80
**LUMO+1**	-1.43	-1.31
**HOMO-2**	-6.64	-5.95
**LUMO+2**	-1.10	-1.24
**HOMO-3**	-7.17	-6.12
**LUMO+3**	-0.89	-1.17

[1] M. J. Frisch, G. W. Trucks, H. B. Schlegel, G. E. Scuseria, M. A. Robb, J. R. Cheeseman, G. Scalmani, V. Barone, B. Mennucci, G. A. Petersson, H. Nakatsuji, M. Caricato, X. Li, H. P. Hratchian, A. F. Izmaylov, J. Bloino, G. Zheng, J. L. Sonnenberg, M. Hada, M. Ehara, K. Toyota, R. Fukuda, J. Hasegawa, M. Ishida, T. Nakajima, Y. Honda, O. Kitao, H. Nakai, T. Vreven, J. A. Montgomery, Jr., J. E. Peralta, F. Ogliaro, M. Bearpark, J. J. Heyd, E. Brothers, K. N. Kudin, V. N. Staroverov, T. Keith, R. Kobayashi, J. Normand, K. Raghavachari, A. Rendell, J. C. Burant, S. S. Iyengar, J. Tomasi, M. Cossi, N. Rega, J. M. Millam, M. Klene, J. E. Knox, J. B. Cross, V. Bakken, C. Adamo, J. Jaramillo, R. Gomperts, R. E. Stratmann, O. Yazyev, A. J. Austin, R. Cammi, C. Pomelli, J. W. Ochterski, R. L. Martin, K. Morokuma, V. G. Zakrzewski, G. A. Voth, P. Salvador, J. J. Dannenberg, S. Dapprich, A. D. Daniels, O. Farkas, J. B. Foresman, J. V. Ortiz, J. Cioslowski, and D. J. Fox, Gaussian, Inc., Wallingford CT, 2013. Gaussian 09: ES64L-G09RevD.01 24-Apr-2013, 26-Jun-2023.
